# Deprotonation-mediated vitrification of organic-inorganic hybrid perovskites

**DOI:** 10.1038/s41467-026-76712-z

**Published:** 2026-08-17

**Authors:** Peng Wang, Guocan Chen, Yuchen Zhu, Zejiang Huang, Zhanpeng Wei, Da Liu, Sihan Zeng, Qing Li, Yuhao Zhao, Haiyang Yuan, Xue Lu Wang, Yu Hou, Hua Gui Yang, Shuang Yang

**Affiliations:** 1 Key Laboratory for Ultrafine Materials of Ministry of Education, Shanghai Engineering Research Center of Hierarchical Nanomaterials, School of Materials Science and Engineering, East China University of Science and Technology, 130 Meilong Road, Shanghai, China; 2 Physics Department & Shanghai Key Laboratory of Magnetic Resonance, School of Physics and Electronic Science, East China Normal University, North Zhongshan Road 3663, Shanghai, China

**Keywords:** Electronic devices, Glasses

## Abstract

Glasses made from organic-inorganic hybrid perovskites are emerging non-crystalline semiconducting materials whose versatile composition and exceptional processability allow for manifold applications. The current melt-quenching approaches are compatible to only few perovskite crystals, as most undergo irreversible decomposition prior to melting. Herein, we report a general flux-mediated approach to produce a library of high-quality perovskite glasses with different organic molecules, metal centers, and framework dimensionality. We show that the partial deprotonation of organic moiety by alkali metal hydroxide flux modulates the intermolecular interactions in perovskites, and activates the flow unit, which depresses the fusion temperature ranging 21–77 °C with flux ratio <1.1 wt%. More critically, the utilization of hydroxide flux creates a large melt window, and enables the thermal vitrification of many inherently non-meltable systems, such as (BA)_2_PbI_4_ (BA = butylamine) and (DGA)PbI_4_ (DGA = 1,1-dimethylbiguanide). Using these semiconductive glasses, we realize high-performance X-ray detection devices showing a sensitivity of 35674.1 μC Gy_air_^−1^ cm^−2^ under 500 V mm^−1^ electric field, and a high-resolution X-ray imaging system via its seamless integration on commercial thin-film transistor backplanes.

## Abstract (web summary)

The discovery of hybrid perovskite glasses opens new opportunities for tailoring the properties of semiconducting materials. Wang *et al*. report a flux-mediated approach for synthesizing a family of perovskite glasses with varied organic constituents while lowering fusion temperatures through deprotonation.

## Introduction

Glass is defined as a non-equilibrium, amorphous substance, which is usually regarded as a frozen liquid and undergoes reversible transformation near the glass transition temperature (*T*_g_). Glassy semiconductors, characterized by the absence of long-range atomic order^1–6^, are indispensable building blocks in modern electronics and photonics^7–10^. Recently, the discovery of organic-inorganic hybrid perovskite glass unlocked unprecedented potential for systematically engineering the chemical composition, electronic structure and functionality of semiconductive glasses^11–14^. These perovskite glasses properly inherit the physical properties from the crystalline analogues, while gaining extended functionality, like melt-processing and integration versatility for potential applications in radiation detection, rewritable optical storage, phase change memory and display technologies^15–23^.

Although perovskite structure offers unlimited chemical and structural diversity^24^, only a few semiconductive perovskite materials thus far have presented the capability to undergo the crystal-to-glass transformation via the common melt-quenching procedure^11,14^, and the resulting glasses often suffer from serious material quality issues, like bubble entrapment, microcracking, and partial decomposition. The fundamental difficulty is that the thermal decomposition at the decomposition temperature (*T*_d_) inevitably occurs below the fusion temperature (*T*_m_) for most perovskites upon the thermal vitrification process^4^. Recent development of perovskite glass materials has mostly followed the design principles of organic-inorganic constituents and spatial arrangements established in crystalline materials^25^, such as the employment of bulky and flexible cations^11,25^, and substitution of electronegative halogen ions and metal centers^4,25^, which have substantially altered the entropic/enthalpic thermodynamics for lowering the crystal-liquid transition temperature^26–30^. However, the glass states are still confined within only few kinds of 1D and 2D hybrid perovskites and 0D metal halides without continuous inorganic skeletons^31–33^. Limited perovskite glass libraries would restrict the systematic exploration of composition-structure-property correlations as well as their technological deployments.

Here, we report a flux-mediated approach to fabricate perovskite glasses that can universally reduce the fusion temperatures of eight types of metal halides by 21–77 °C with trace doping level <1.1 wt%. We find that a deprotonation reaction of hydroxide flux to the ammonium cations in perovskites weakens the intermolecular interactions and reduces the fusion enthalpy (Δ*H*_fus_), resulting in the exceptional *T*_m_ depression. The resulting (DGA)PbBr_4_ (DGA = 1,1-dimethylbiguanide) glass maintains excellent semiconducting property with a sensitivity of 35674.1 μC Gy_air_^−1^ cm^−2^ under 500 V mm^−1^ electric field, and can be conformally melt-deposited onto commercial thin-film transistor (TFT) arrays for high-resolution X-ray imaging application. Furthermore, we also show that some inherently infusible perovskite, like (BA)_2_PbI_4_ (BA = butylamine), whose *T*_d_-*T*_m_ is −26 °C can be safely melt-processed by using only 1.1 wt% LiOH flux. The synthetic strategy in this work sparks a paradigm shift for expanding the family and diversity of perovskite glasses regardless of the inherent thermal decomposition behavior of the crystalline phase.

## Results

### Flux-mediated fabrication of perovskite glass

**Fig. 1:**
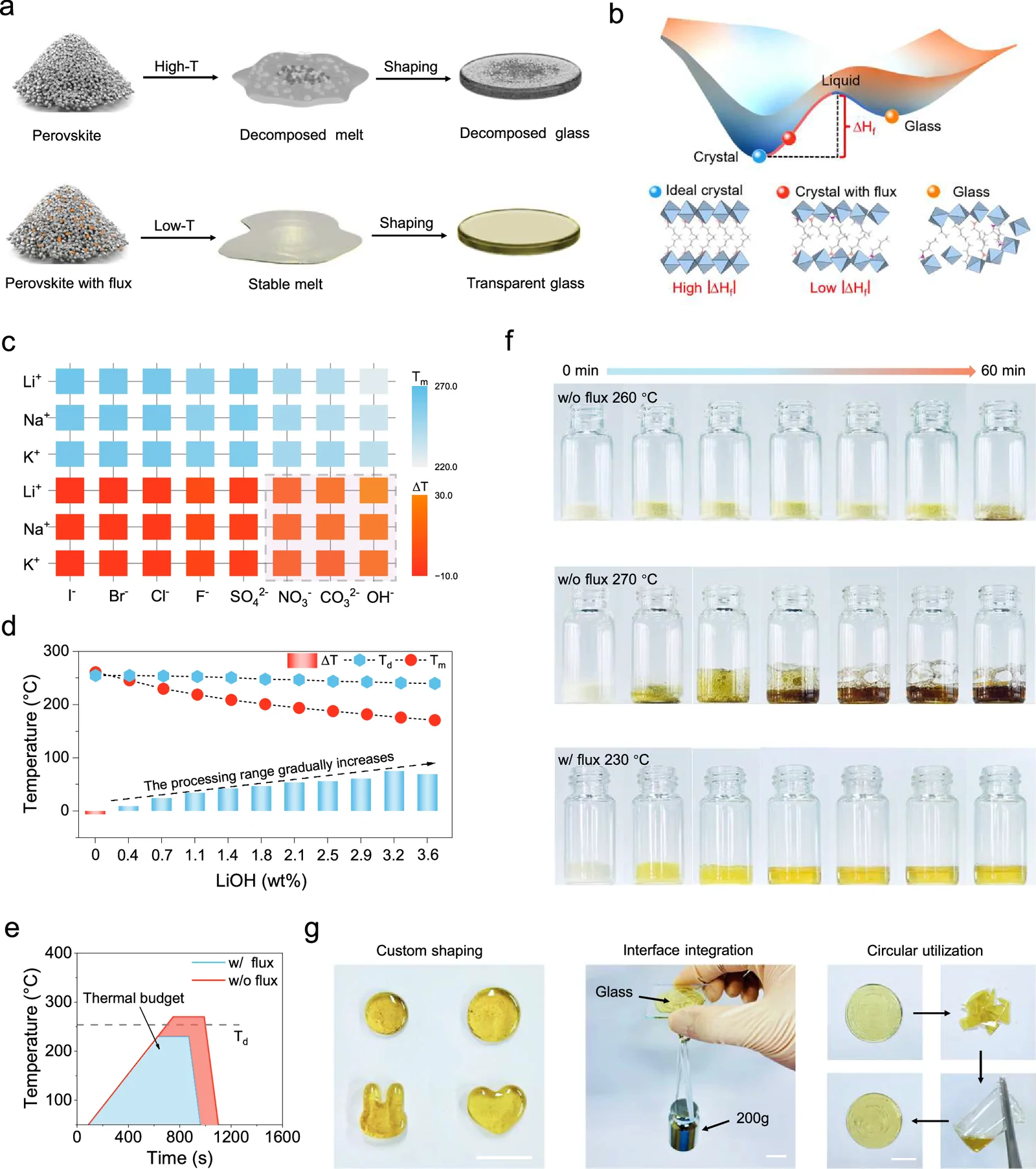
Flux-mediated synthesis of perovskite glass. **a** Schemic illustration of two fabrication procedures of perovskite glass. The traditional method that needs high temperature treatment often produces amorphous phase with many decomposed species. The flux-assisted method enables a stable perovskite melt at a low temperature and high-quality glass. **b** Potential energy diagram showing the Gibbs free energy of the amorphous and the crystalline phases. The increase in formation energy caused by deprotonation makes it easier to cross the energy barrier to reach the molten state. **c** The *T*_m_ and Δ*T* (defined as *T*_d_ - *T*_m_) diagrams of DPB perovskite with different types of fluxes. A large Δ*T* corresponds to wide temperature window for the melt-processing of perovskite glass. **d** Plots of *T*_m_, *T*_d_, and Δ*T* for DPB perovskite with as a function of LiOH ratios. **e** Thermal budget control for perovskite glass processing by using flux. **f** Time-lapse images of perovskite powders at their respective melting points over distinct time intervals. The sample with LiOH can be melt at 230 °C without observable decomposition. **g** Advantages of perovskite glasses prepared by the flux-mediated approach (scale bars: 2 cm). The flux-assisted fabrication of DPB glass allows multiple functionalities, including cast molding, substrate integration and cyclic utilization.

The synthesis of perovskite glass typically involves the initial thermal melting of crystalline phase and the subsequent quenching to form a supercooled liquid state (Fig. 1a). However, this fabrication protocol has been challenged by thermal decomposition of molten phase that happens in most established perovskites^25^. Drawing on the wisdom of traditional glass chemistry, we implemented various fluxes into perovskite materials, which would modify the network structure and potentially reduce their melting temperature (see details in Experimental Section). As a consequence, the free energy of the flux-containing crystalline state will be elevated, meaning that less thermal energy is required to cross the energy barrier for melting (Fig. 1b). To explore the *T*_m_ depression effect of different fluxes on perovskites, we selected (DGA)PbBr_4_ perovskite (DPB, see crystal structure in Supplementary Fig. 1) developed by our group as a model system, whose *T*_m_ and *T*_d_, as determined by differential scanning calorimetry (DSC) and thermogravimetric analysis (TGA), are 258.5 °C and 254 °C, respectively (Supplementary Figs. 2 and 3), delivering a narrow melt-processing window of only −4.5 °C. We systematically examined the evolution of *T*_m_ and Δ*T* (defined as *T*_d_-*T*_m_) values of DPB by using 24 alkali metal salts with different cationic (K⁺, Na⁺, Li⁺) and anionic (F⁻, Cl⁻, Br⁻, I⁻, SO_4_^2^⁻, NO_3_⁻, CO_3_^2^⁻, OH⁻) components, and the results are summarized in Fig. 1c. Molar ratio of fluxes was kept constant (30%) in these experiments, and the corresponding mass ratios are listed in Supplementary Table 1. The *T*_m_ of DPB is almost independent of the types of alkali metal cations, with *T*_m_ variation <3 °C, since many hybrid perovskites can accommodate these species without changing the host structure (Supplementary Fig. 1 and Table 2). Similar trend can be observed in DPB with metal halides fluxes, in which the Cl⁻, Br⁻, or I⁻ are also basic units of perovskite structure. In striking contrast, fluxes with oxoacid and hydroxide anions dramatically contribute to the *T*_m_ depression in perovskites: the *T*_m_ of DPB with LiOH, Li_2_CO_3_, LiNO_3_, and Li_2_SO_4_ are 225 °C, 240 °C, 245 °C, and 262 °C, respectively. In principle, oxyanions (e.g., CO_3_^2^⁻, SO_4_^2^⁻ and NO_3_⁻) can react with organic ammonium through proton transfer reaction to form oxyacid, followed by decomposition into gaseous products at elevated temperatures (Supplementary Note 1). Because the decomposition temperature of oxyacid follows the consequence of H_2_SO_4_ > HNO_3_ > H_2_CO_3_, the highly reactive CO_3_^2^⁻ should be more effective as a network modifier and lead to larger *T*_m_ variation, fully consistent with experimental observations. As a strong proton acceptor, OH⁻ exhibits the highest reactivity to organic ammoniums, and yields volatile water molecules as a byproduct, which is responsible for the largest Δ*T* of +29 °C. Afterward, we elaborated on the impact of LiOH flux ratio on the melting thermodynamics of DPB perovskites. Thermal analysis results in Fig. 1d reveal the gradual *T*_m_ reduction from 258.5 °C without LiOH to 170 °C with 3.65 wt% LiOH, along with the Δ*T* increase from −4.5 °C to +69 °C, while the *T*_d_ remains almost stationary. However, the presence of excess flux may reversely activate the mobility of glass constituents that behaves as reduced glass transition temperatures (*T*_g_), for instance, the *T*_g_ declined from 54 °C to 52.5 °C by using 1.1 wt% LiOH (Supplementary Fig. 4). Therefore, we adopted a relatively low LiOH ratio of 1.1 wt% to leverage the *T*_m_ and *T*_g_ relationship for DPB glass in the following study.

The effective *T*_m_ reduction by the flux-mediated approach opens the possibilities for harvesting stable perovskite melt under a wide temperature regime before degradation, which is anticipated to reduce the thermal budget for glass preparation (Fig. 1e). Pure DPB perovskites experienced solid-liquid phase transition along with obvious bubble formation and discoloration at 270 °C, yet it would sustain the original solid state under 260 °C and 230 °C (Fig. 1f and Supplementary Fig. 5). The incorporation of LiOH flux permitted wide temperature and time windows for DPB glass fabrication: the yellowish liquid product is transparent, flowable and stable at 230 °C during 60 min (Fig. 1f and Supplementary Fig. 6). After the melt quenching procedure, we successfully fabricated high quality, homogeneous DPB glass samples (a-DPB-flux) with precisely engineered dimensions and shapes (Fig. 1g and Supplementary Fig. 7).

### Universal fabrication of hybrid metal halide glasses

**Fig. 2:**
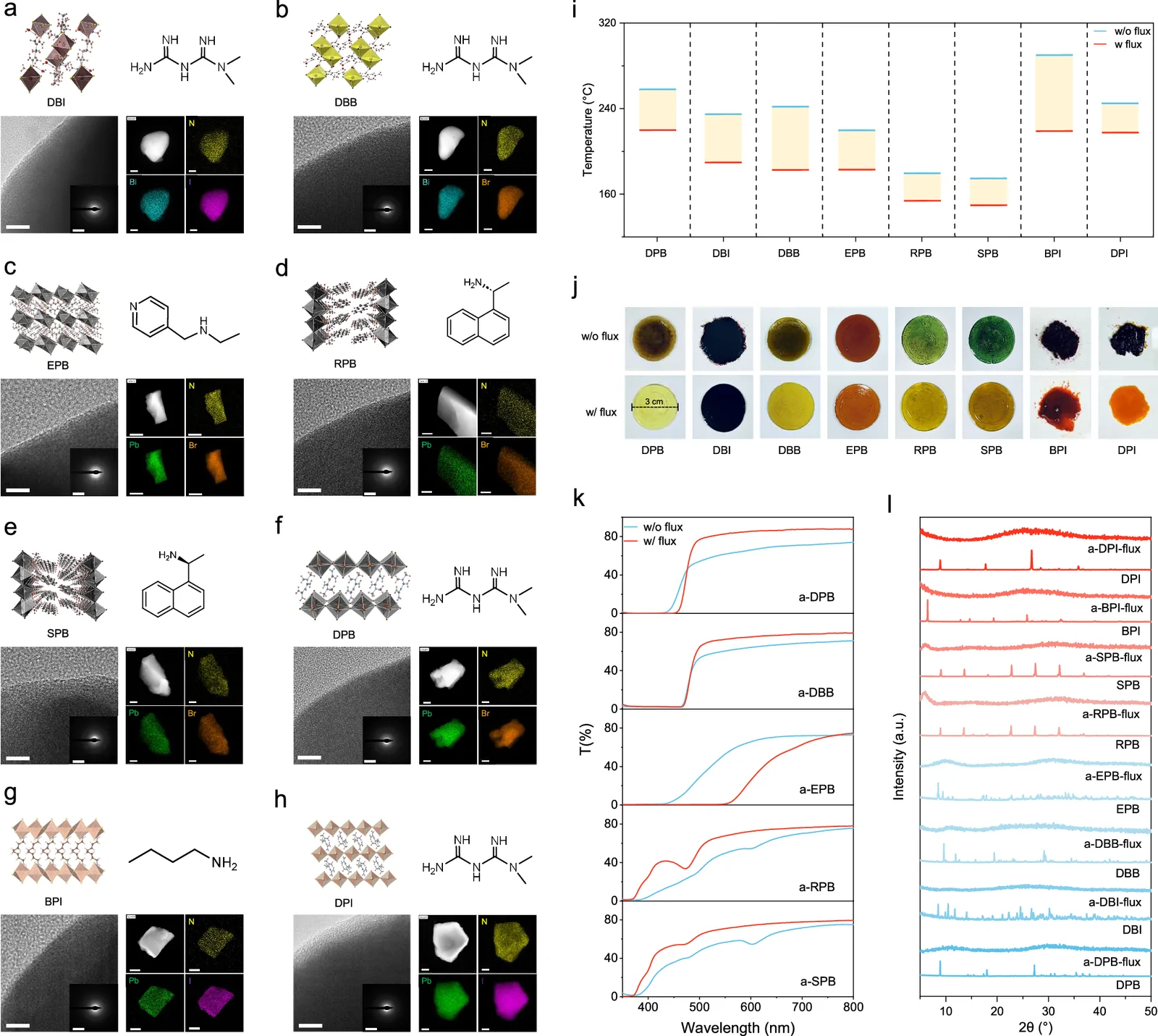
Application of flux-mediated strategy in different metal halide materials. **a**–**h** Atomic crystal structure of the hybrid metal halide materials, along with HR-TEM images (Fig. 2a–f, scale bars: 10 nm; Fig. 2g, h, scale bars: 5 nm), SAED patterns (scale bars: 5 nm^−1^), and EDS mapping (Fig. 2a, scale bar: 100 nm; Fig. 2c, h, scale bars: 500 nm; Fig. 2b, d–g, scale bars: 200 nm) of the corresponding glasses. **i** *T*_m_ values of various perovskites without and with LiOH fluxes. The reduction of *T*_m_ values by LiOH fluxes ranges from 21 to 77 °C. **j** Photographs of 3 cm diameter glass sheets processed without and with LiOH fluxes. Glass sheets with LiOH fluxes demonstrated superior formability and high transmittance. **k** Transmittance spectra of doped and undoped glass. (thickness: 0.5 mm). **l** XRD patterns of the perovskite crystals and corresponding glass fabricated via the flux-mediated approach.

We next extended our synthetic strategy to five more hybrid metal halide materials with glass-forming potential, i.e., (DGA)_2_Bi_2_I_10_·2H_2_O (DBI), (DGA)_2_Bi_2_Br_10_·4H_2_O (DBB), (4-EAMP)_2_Pb_3_Br_10_ (EPB, 4-EAMP = 4-(ethylaminomethyl)pyridine), (R-NEA)_2_PbBr_4_ (RPB, R-NEA = (R)-(+)−1-(1-naphthyl)ethylamine) and (S-NEA)_2_PbBr_4_ (SPB, S-NEA = (S)-(-)−1-(1-naphthyl)ethylamine). The first three compounds (DBI, DBB, and EPB) synthesized by our research group are crystallized in the monoclinic space group *P2*_*1*_*/n* for DBI, triclinic space group *P-1* for DBB and monoclinic space group *C2/c* for EPB, respectively (Fig. 2a–c and Supplementary Table 3, 4)^34^. The latter two compounds (RPB and SPB), initially reported by Mitzi et al., have a typical Ruddlesden-Popper (RP) type perovskite structure with reversible crystal-glass transition behavior (Fig. 2d, e)^11^. The diverse crystalline structures result in significantly distinct melting behaviors among these compounds: the *T*_m_ are 258.5 °C for DPB, 242 °C for DBI, 235 °C for DBB, 220 °C for EPB, 180 °C for RPB, and 175 °C for SPB, yielding Δ*T* values of −4.5 °C, −8 °C, +52 °C, +76 °C, +24 °C, and +29 °C, respectively (Supplementary Figs. 8–13)^34^. Despite the large difference in thermal properties of these hybrid materials, we found that our flux-mediated strategy functioned effectively in lowering the *T*_m_ values and promoting the melting process for all investigated systems: the utilization of LiOH flux depressed the *T*_m_ ranging from 25 °C to 59 °C for these materials, and thus created widened temperature thresholds of 31–100 °C for melt-fabrication (Fig. 2i and Supplementary Figs. 14–16). In Fig. 2j, we show many compounds possessed without flux suffered from severe bubble generation and color change upon processing at the original *T*_m_, wherein the a-DBI cannot even be thermoformed into regular sheets due to the poor fluidity and rapid degradation. When flux was utilized, all those materials were successfully melt-pressed into 3 cm diameter glass sheets with superior transparency. The quality of the metal halide glasses can be quantitatively rationalized in terms of the optical transmittance (Supplementary Fig. 17). The occurrence of darkened decomposition product in the hybrid glass was embodied as the onset redshift and low transmittance in the visible region (Fig. 2k). Critically, the usage of LiOH flux enhanced the transmittance of all glasses (approximately 500 μm thick, except a-DBI with narrow bandgap) to approximately 73–87% at the wavelength region of 500–800 nm. Subsequent inspections by optical microscopy and SEM unveils the smooth, compact surface and bubble-free interior of the flux-containing glasses (Supplementary Figs. 18 and 19). High resolution transmission electron microscopy (HR-TEM) images, selected-area electron diffraction (SAED) patterns and corresponding energy-dispersive X-ray spectroscopy (EDS) mapping further validate the amorphous nature with outstanding compositional homogeneity in the scanned region of the flux-containing glasses (Fig. 2a–f), whereas the X-ray diffraction (XRD) characterizations evidence the non-crystalline structure of these glass powders (Fig. 2l). Apart from the high-quality of the resulting glasses, the use of flux-mediated methods has also enhanced the glass-forming ability (GFA), as evaluated according to the Turnbull criterion. Thermal analysis data confirm that the *T*_g_/*T*_m_ values of the glass prepared using this strategy meet the Turnbull standard for glass-forming ability (*T*_g_/*T*_m_ > 0.67), indicating the structural stability of the amorphous phase (Supplementary Fig. 20).

The success of the flux-mediated approach raises the question of whether it can be extended to inherently infusible perovskites that are fundamentally unable to form stable melts or glasses. Two typical 2D perovskites, (BA)_2_PbI_4_ (BPI) and (DGA)PbI_4_ (DPI), were adopted with layered RP and Dion-Jacobson (DJ) type structures, respectively (Fig. 2g, h)^25,35^. Both materials suffer from lower *T*_d_ values (270 °C for BPI and 243 °C for DPI) than *T*_m_ (296 °C for BPI and 246 °C for DPI) (Supplementary Figs. 21 and 22), yielding temperature deficits of −26 °C for BPI and −3 °C for DPI, respectively. By using 1.1 wt% LiOH flux, the *T*_m_ of BPI progressively depressed to 219 °C, and the melt-processing window transformed from a negative to a positive region with a large Δ*T* = +46 °C. A similar thermal phenomenon was observed in the circumstance of DPI where the addition of 0.8 wt% LiOH flux enabled a positive Δ*T* = +21 °C (Fig. 2i). Encouragingly, we obtained stable BPI and DPI melts without observable decomposition, and successfully fabricated respective glasses for the first time (Fig. 2j). After vitrification, the *T*_g_ values of a-BPI-flux and a-DPI-flux were measured to be 79 °C and 98 °C, respectively (Supplementary Figs. 23 and 24). HR-TEM images, SAED patterns and EDS mapping results of both samples confirm the amorphous structure as well as homogeneous distributions of nitrogen, lead, and iodine elements (Fig. 2g, h), while the broad hump profiles in corresponding XRD patterns confirm the absence of long-range order at the macroscopic scale (Fig. 2l).

Although the existence of flux activates the internal component dynamics, it did not affect the decomposition of the organic moiety: ^1^H NMR results confirmed identical organic structure of BPI and DPI before and after vitrification, except for a slight upfield shift of amine proton peaks due to deprotonation (Supplementary Fig. 25)^36–38^. The minimization of BPI decomposition results in a very similar bandgap of the crystalline and glass phases (Supplementary Fig. 26), which likely originates from the lower processing temperature and minimized loss of organic components that preserve the local Pb–I coordination motif^4,13,15,39^. Furthermore, DSC measurements over six repeated heating-cooling cycles of a-BPI-flux exhibited minimal fluctuations in *T*_*m*_ and crystallization temperature (*T*_c_) signals, demonstrating robust thermal stability and reversible crystal-glass transition within the applied temperature range (Supplementary Fig. 27). Optical transmittance of a-BPI-flux and a-DPI-flux at a thickness of 0.5 mm reaches 45% and 68%, respectively, within the 550–800 nm spectral region (Supplementary Fig. 28). Therefore, the flux-mediated approach can prevent the organic component loss in repeated processing, thereby opens up a new avenue for sustainable reuse of hybrid perovskite glasses.

### Flux chemistry in perovskite glass

**Fig. 3:**
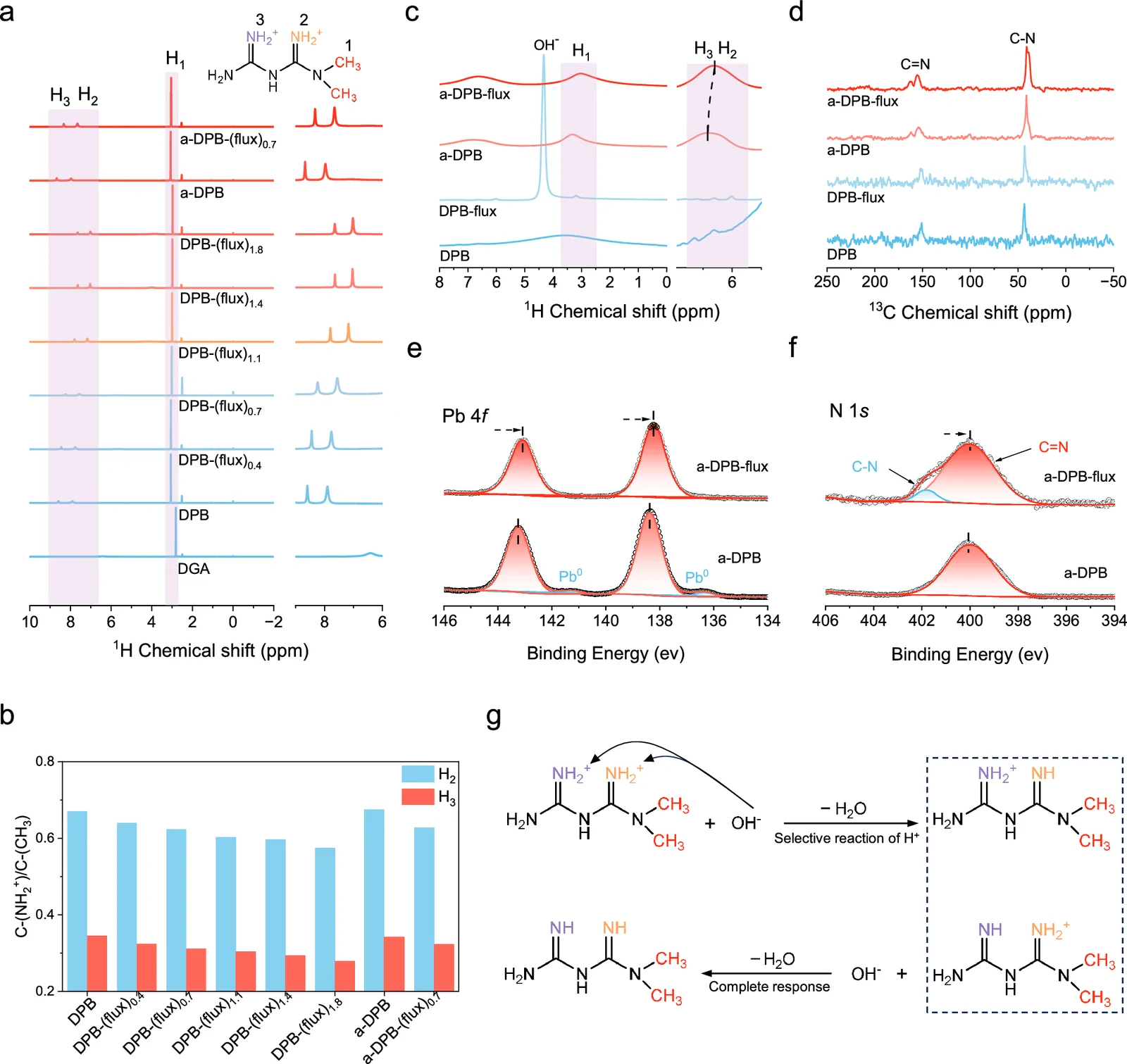
Deprotonation reaction of perovskite with hydroxide flux. **a** ^1^H NMR spectra of DPB crystals and glass with varying LiOH contents in DMSO. Inset is the molecular structure of protonated DGA. The chemical shift of hydrogen signal from amine groups indicates the deprotonation reaction between the hydroxide and guanidinium cations. Solid-state **b** Quantitative analysis of H_2_/H_1_ and H_3_/H_1_ ratios derived from ^1^H NMR peak area of DPB crystals and glass with different LiOH amounts. The content of protonated amine groups progressively decreased relative to methyl groups with increasing doping levels. H_2_ depletion occurs slightly faster than H_3_ site due to the distinction in chemical environments. **c** ^1^H NMR and **d** ^13^C NMR spectra of doped and undoped DPB crystals and glass. XPS spectra of **e** Pb 4 *f* and **f** N 1 *s* of doped and undoped DPB glass. **g** Schematic diagram of the deprotonation reaction process of protonated DGA with alkali metal hydroxides.

In the presence of hydroxide flux, the fusion of metal halide perovskites is likely to be initialized by an acid-base neutralization reaction between hydroxide and ammonium molecules^40,41^. To monitor the underlying chemical reaction, we conducted ^1^H Nuclear magnetic resonance **(**NMR) measurements of a series of DPB samples with varying LiOH doping levels in DMSO-d_6_ solution, and the results are shown Fig. 3a. Characteristic peaks at 3.05 ppm, 7.91 ppm and 8.6 ppm correspond to H_1_ from methyl groups, H_2_ and H_3_ from protonated amine groups (labeled in the inset of Fig. 3a), respectively, while the unprotonated amine hydrogens appear as a broad peak in the high field^42,43^. As the LiOH content increases, the H_2_ and H_3_ resonances progressively shift upfield in both perovskite and glass phases, indicating reduced electron density around exchangeable N–H protons due to deprotonation reaction^44,45^. Quantitative analysis of the integrated peak areas of the characteristic N–H proton and methyl proton signals reveals the negative dependance of protonated amine concentration on the amount of LiOH flux, whereby approximately 75 ~ 85% of the hydroxide ions participate in deprotonation, with potential discrepancies arising from measurement limitations (Fig. 3b)^46^.

Solid-state NMR spectroscopy offers more realistic evidence for the deprotonation reaction under native solvent-free environments^47^. Analogous to the solution-state ^1^H NMR results, an upfield shift of N–H proton signals occurs in solid-state ^1^H NMR spectra of both crystals and glasses with LiOH flux (Fig. 3c). The disordered nature of these glassy materials, combined with the resolution constraints of solid-state NMR, resulted in merged H_2_/H_3_ resonances as a signal broad peak in all samples. An additional peak corresponding to hydroxyl hydrogen emerged in DPB-flux, but disappeared in a-DPB-flux, because of diffusivity-limited neutralization reaction upon mechanical grinding prior to heating. Solid-state ^13^C NMR spectra in Fig. 3d show that the presence of flux minimally impacts the C = N signals in both crystalline and glassy states. However, due to the redistribution of the electron cloud around the C atom during the transition from crystal to glass, this resonance splits during vitrification, which is consistent with the C = N stretch shift in the Fourier transform infrared (FTIR) spectra (Supplementary Fig. 29). In particular, deconvolution of X-ray photoelectron spectroscopy (XPS) Pb 4 *f* spectra reveals a discernible Pb^0^ peak at 136.4 eV and 141.3 eV for a-DPB, as one decomposition product upon the melt-processing procedure, whereas this peak is absent for a-DPB-flux (Fig. 3e). Concomitantly, an extra C–N species at binding energy of 401.85 eV was identified in the XPS N 1 *s* spectra of a-DPB-flux, in agreement with the proposed deprotonation mechanism where the electron density on nitrogen atoms is improved through N–H proton dissociation (Figs. 3f, g)^48^. The consumption of hydroxyl also shifted the Li 1 s peak from 55.85 eV to 55.15 eV, as the coordination environment of Li^+^ counter ions was changed from hydroxide to bromide anions (Supplementary Fig. 30)^49^. Besides the neutralization reaction with ammonium molecules, hydroxide ions from LiOH may also interact with lead ions to form lead hydroxide (Pb(OH)_2_). Our Raman measurements rule out this reaction mechanism, since only Pb–Br vibrational modes (40–150 cm^−1^), other than Pb–O vibrations (250–400 cm^−1^), can be probed in all samples (Supplementary Fig. 31).

**Fig. 4:**
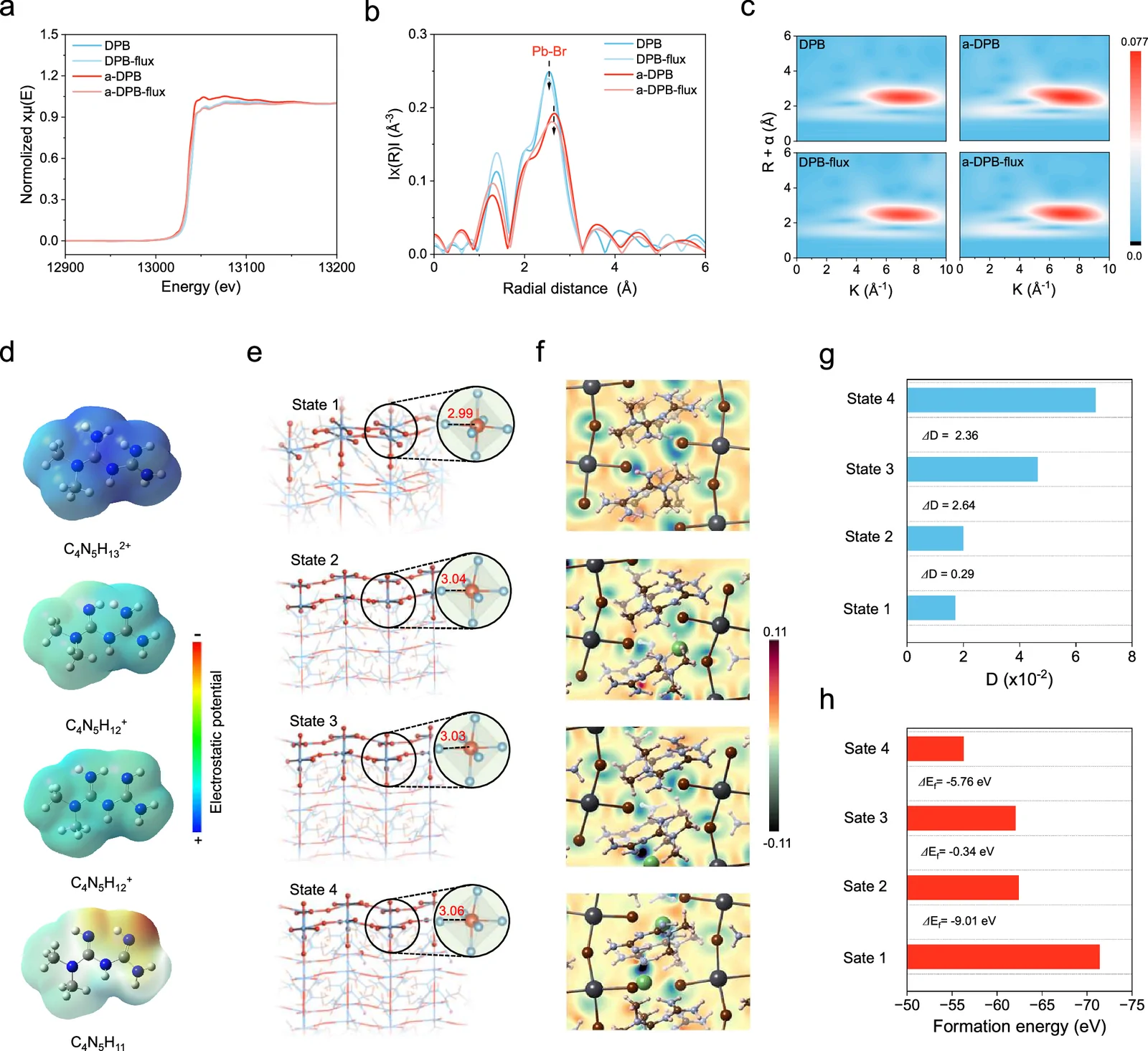
Structural evolution characteristics of perovskite under flux doping. **a** Pb K-edge XANES spectra, **b** Fourier-transform *k*^3^-weighted *χ*(*k*) functions and **c** Wavelet-transform plots of *k*^3^-weighted EXAFS signals of doped and undoped samples. **d** Electrostatic potential of DGA molecules at different protonation states. Red and blue regions represent negative and positive electrostatic potential values, respectively. In the overlaid molecular structures, carbon, nitrogen, and hydrogen atoms are depicted in gray, blue, and white, respectively. **e** The atomic structural evolution of DPB perovskite with varying degrees of deprotonation: pristine structure without deprotonation (state 1), partial deprotonation at H_2_ or H_3_ site (states 2 and 3), and full deprotonation at both sites (state 4). Deprotonation of the organic cations would lead to octahedral distortions in the inorganic framework, which manifests as structural variations in bond lengths and bond angles. **f** Differential charge density analysis, **g** distortion index (*D*) and **h** formation energy of octahedral layers for the four corresponding states. The deprotonation process is associated with a monotonic reduction in formation energy along with pronounced octahedron distortion.

Aforementioned analyses have established the deprotonation reaction mechanism of hydroxide flux with the organic cations in hybrid perovskites, but further investigations are required to elucidate the subsequent structural and chemical evolution with respect to inorganic framework structure, intermolecular interaction, and enthalpy-entropy variation. X-ray absorption spectroscopy (XAS) measurements were performed to probe the local coordination environments of the inorganic networks of DPB crystals and glasses. As shown in Fig. 4a, the nearly identical absorption edge positions in X-ray absorption near-edge structure (XANES) spectra of these samples confirm the retained Pb^2+^ oxidation state after vitrification. Fourier-transform extended X-ray absorption fine structure (FT-EXAFS) spectra and corresponding Morlet wavelet transform results accentuate reduced Pb coordination number in the sequence of DPB > DPB-flux > a-DPB > a-DPB-flux, as reflected by the intensity of the characteristic peaks (Fig. 4b, c). Concurrently, Pb–Br bond elongation, as measured by the peak shift from 2.54–2.51 Å of the crystals to 2.66–2.63 Å of the glasses, happened in the crystal-to-glass transition regardless of the presence of flux. These structural observations can be interpreted as two basic aspects: the prerequisite for the melting of perovskites is the partial depolymerization of inorganic networks to activate the flow units, and the deprotonation reaction with flux can further destabilize the inorganic framework for promoting the fusion events.

The bonding character of organic cations to the inorganic framework can be evaluated by the electrostatic potential surface (EPS) analysis of the surrounding molecular environments^50^. When increasing the deprotonation degree, electrostatic potential of the DGA molecule gradually shifts from positive to negative polarity, and eventually inverses at the completely deprotonated guanidine group, because of the loss of electropositive protons (Fig. 4d). The variation in charge density weakens the original organic-inorganic interactions, and elongates the Pb–Br bond in the crystalline structure: removal of H_2_/H_3_ protons extends bond length from 2.99–3.06 Å (Fig. 4e). Further differential charge density maps in Fig. 4f visualize the intermolecular bonding mode of the DGA to inorganic networks: in the initial state, the molecules electrostatically interact with Br⁻ without shared electron density to stabilize the structure; the loss of hydrogen species reduces the electron cloud density around original positions; finally, the imino nitrogen atoms that have lost protons in DGA move away from the halide anions and coordinate to lithium ions, thereby enhancing the molecular freedom. Meanwhile, the Li^+^ ions from LiOH flux that can provide charge compensation for the partially deprotonated organic moieties, closely interact with Br⁻ and deprotonated imino N sites at the deprotonated organic/halide interface in all models (Supplementary Fig. 32). Such a coordination environment would restrict the movement of Li^+^. Quantitative analysis of the distortion index (*D*) of the inorganic octahedra networks reveals the increase of *D* from 0.01708 to 0.06994 upon dual deprotonation (Fig. 4g). As a consequence, the formation energy of crystalline DPB increases from −71.38 eV to −56.27 eV upon the deprotonation process (Fig. 4h), corresponding to reduced energy barriers for crystal-to-melt transition. Therefore, the trace flux doping would not fully disintegrate the crystalline phase before heating, but would marginally disrupt the crystalline structure, change the intermolecular coordinating environment, and subsequently contribute to a reduced Δ*H*_fus_ of the perovskite crystals (Supplementary Fig. 33, Supplementary Note 2 and Supplementary Table 5)^29,33^.

### Radiation detection performance

**Fig. 5:**
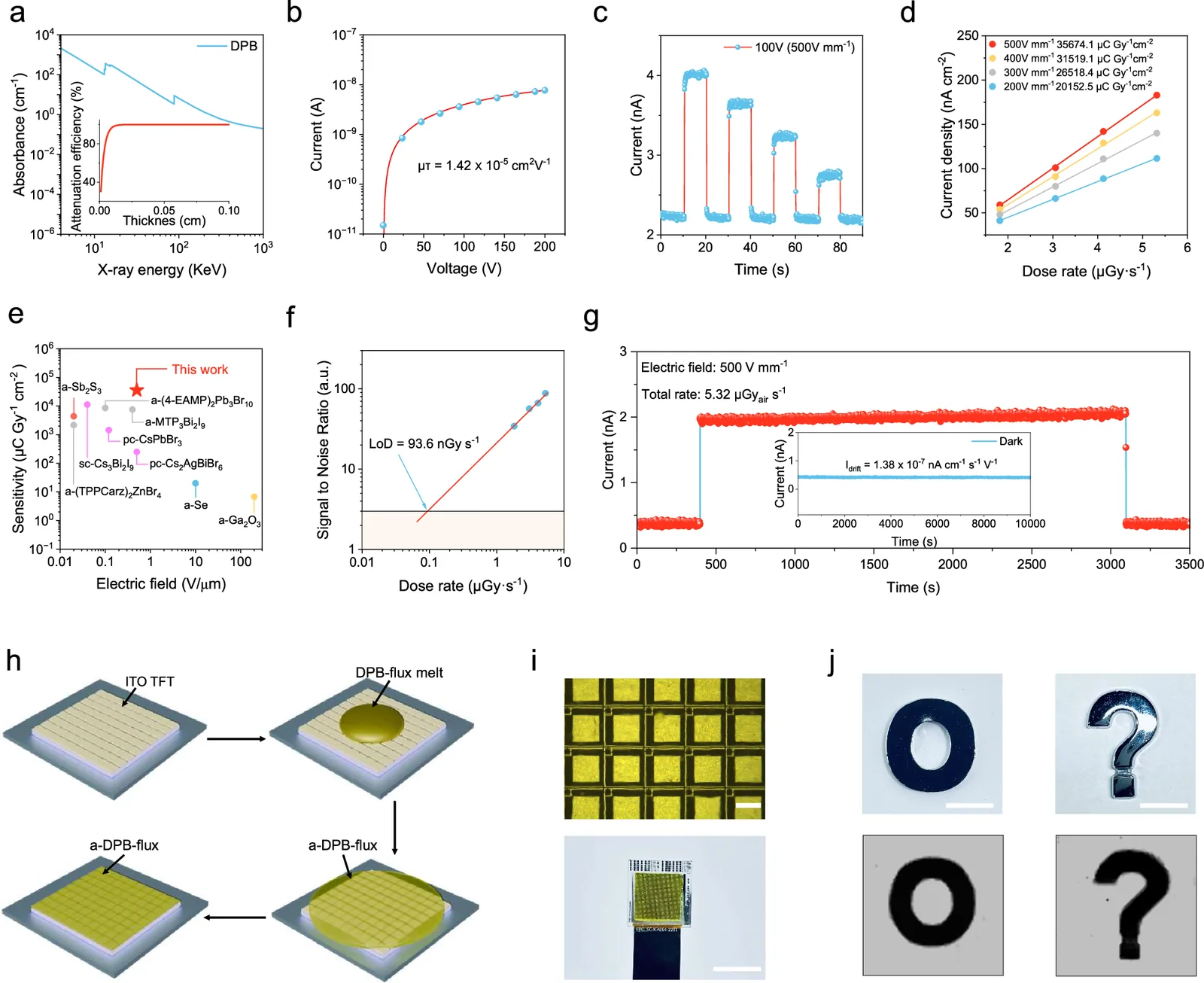
Radiation detection device based on a-DPB-flux glass. **a** X-ray photon attenuation coefficient of DPB, with an inset showing calculated X-ray attenuation efficiency. **b** Photoconductivity characterization of a-DPB-flux device. **c** Temporal response of a-DPB-flux device under varying X-ray dose rates. **d** Dose rate-dependent output current density of a-DPB-flux device. **e** Comparison of the sensitivity of amorphous and polycrystalline-based X-ray detectors. The prefixes of a-, pc-, and sc- denote amorphous, polycrystalline, and single-crystal samples, respectively. **f** Dose rate-dependent SNR of the a-DPB-flux device. **g** The radiation stability of the a-DPB-flux device was evaluated under a dose rate of 5.32 μGy_air_ s^−1^ and a bias voltage of 500 V mm^−1^. Inset shows the dark current stability of the device. **h** Schematic diagram of the assembly procedure of the a-DPB-flux device on the TFT backplane. **i** Optical micrograph of pixel units in a-DPB-flux integrated TFT device and photographs of TFT device integrated with a-DPB-flux plate (scale bars: 250 μm and 3 cm). **j** X-ray imaging of metallic symbols captured by a-DPB-flux integrated TFT array at 5.32 μGy_air_ s^−1^ dose rate with 15 V bias (scale bars: 1 cm).

Unlike its crystalline counterparts that are often fabricated with toxic polar solvents, the low-temperature melting behavior of perovskite glasses endows them with excellent solvent- and vacuum-free processability on various substrates towards opto-electronic applications^51,52^. The selected 2D perovskite materials are potentially useful in radiation detection applications, as their high atomic number and layered structure enable efficient radiation attenuation and favorable charge transport characteristics^53^. For example, a 0.1-mm-thick DPB sample achieves an attenuation efficiency of 97.3% for 40 keV X-ray photons (Fig. 5a). The resistivity *ρ* of a-DPB-flux derived from dark current-voltage (I–V) characteristics attained 2.25 × 10^9^ Ω cm (Supplementary Fig. 34). Carrier mobility-lifetime product (*μτ*), a parameter quantifying how far charge carriers can drift before recombination, serves as one key figure-of-merit for a radiation detection device. By fitting photoconductivity data with the modified Hecht equation, we find that the addition of LiOH slightly reduces the *μτ* product of the crystals, suggestive of the formation of trap states (Supplementary Fig. 35). In the glassy phase, a-DPB-flux achieves a *μτ* of 1.42 × 10^−5^ cm^2^ V^−1^ (Fig. 5b), whereas a-DPB without flux shows a drastically lower *μτ* of 6.57 × 10^−6^ cm^2^ V^−1^ due to thermal decomposition. Thus, the utilization of flux lowers the processing temperature to suppress the thermal decomposition, and thereby benefits charge transport in a-DPB-flux.

We next measured the response currents of the a-DPB-flux device (Ag/glass/Ag) under varying X-ray dose rates and electric fields to estimate its detection performance (Fig. 5c, d). At electric fields of 200–500 V mm^−1^, the a-DPB-flux device displays stable current signals, rapid X-ray response, and a linear correlation with the incident X-ray dose rate (Supplementary Fig. 36). The sensitivity increases as a function of high electric fields due to the bias-driven carrier collection, and achieves a maximum value of 35674.1 μC Gy_air_^−1^ cm^−2^ at 500 V mm^−1^ among the top-tier level for amorphous semiconducting materials (Fig. 5e and Supplementary Table 6)^32,54^. Minimum detectable limit (MDL), which reflects the ability of detectors to resolve weak signals, is another crucial metric for practical X-ray applications. By extrapolating the dose-rate-dependent signal-to-noise ratio (SNR) to a threshold of 3, the theoretical MDL of the a-DPB-flux detector was determined to be 93.6 nGy s^−1^ (Fig. 5f). Given that the *T*_g_ of a-DPB-flux is well above room temperature, ionic current that typically manifests as the baseline drift or signal shift is not expected during device operation. The stability measurements in Fig. 5g show the dark current drift of only 1.38 × 10^−7^ nA cm^−1^ s^−1^ V^−1^ under an electric field of 500 V mm^−1^, as well as a stable photocurrent under continuous X-ray irradiation at 5.32 μGy_air_ s^−1^. The overlapping of I–V curves measured by opposite scans of the a-DPB-flux device confirms its tolerance to electric field and X-ray radiation (Supplementary Fig. 37). These results demonstrate the device’s significant tolerance to humidity, residual ions, electric field, and X-ray radiation.

The melt-processing of perovskite glass is particularly suitable for device integration on commercial thin-film transistor (TFT) pixel circuits for X-ray imaging functions (Fig. 5h). The melt-pressing procedure was performed at 220 °C, remaining below the processing limit of IGZO TFT backplanes (230 °C). The fluidity of DPB melt guarantees the uniform interfacial contact without any bubbles or voids (Fig. 5i and Supplementary Fig. 38), whereas its viscosity imparts mechanical robustness to the resulting glass/TFT junction. To evaluate its potential for large-area imaging, we fabricated a large-area (4 cm in diameter) a-DPB-flux device and deposited a 5 × 5 array of silver electrodes on its surface (Supplementary Fig. 39). The resistivities measured at the 25 pixels exhibited excellent uniformity, which is beneficial for large-area flat-panel X-ray imaging. Under a dose rate of 5.32 μGy_air_ s^−1^, the imaging capability of the a-DPB-flux glass array was illustrated by visualizing a metallic symbol, where the object’s contour could be clearly resolved in the reconstructed images acquired from the readout circuit (Fig. 5j and Supplementary Fig. 40).

### Recycling roadmap of perovskite glass devices

**Fig. 6:**
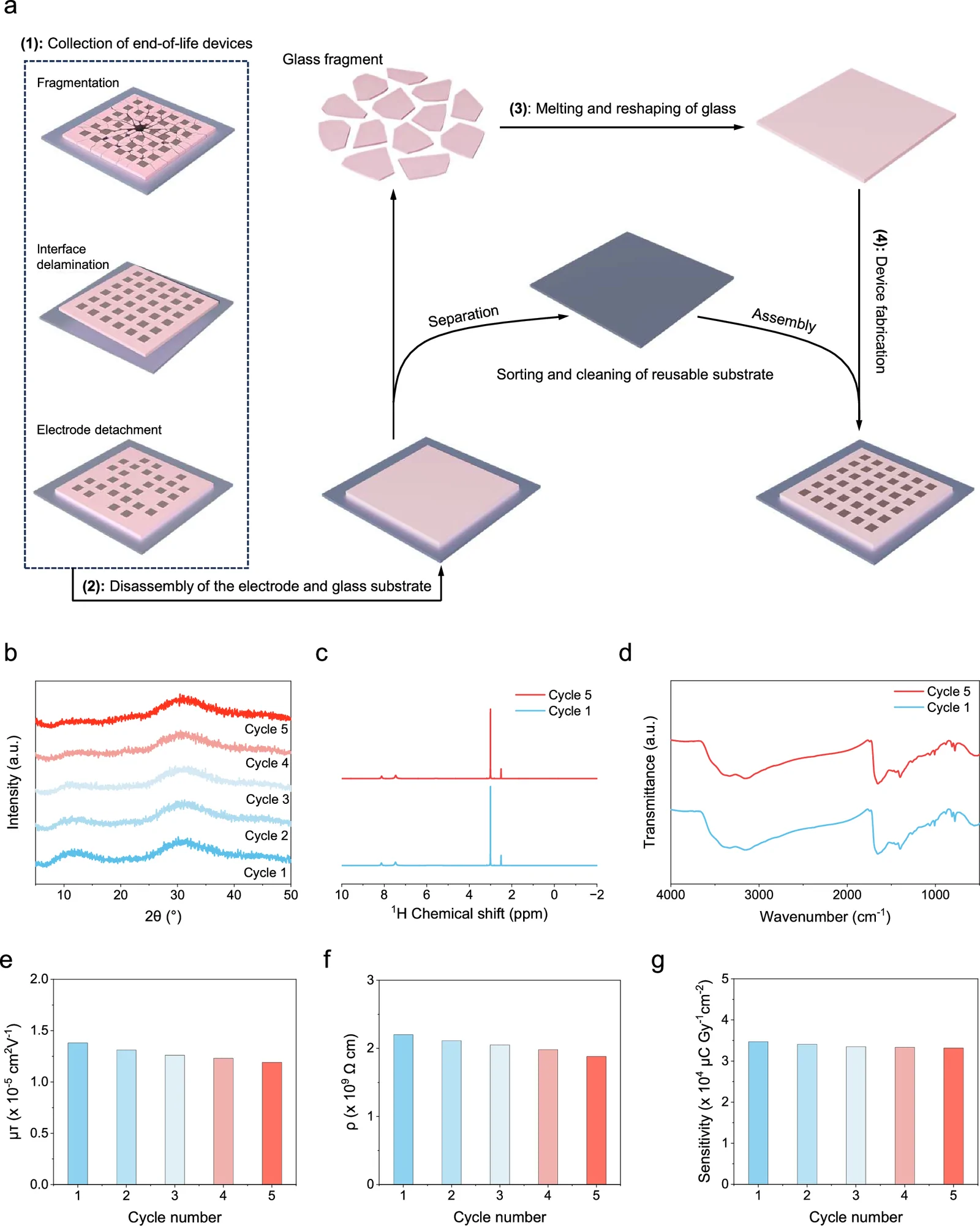
Sustainable recycling of a-DPB-flux device. **a** Schematic diagram of the recycling process of decommissioned devices. **b** XRD patterns of a-DPB-flux glass made from the 1st to the 5th cycle. **c** ^1^H NMR and **d** FTIR spectra of a-DPB-flux glass processed at the 1st and 5th cycles. Comparison of the **e** *μτ* product, **f** resistivity, and **g** detection sensitivity for a-DPB-flux glass detectors made from the 1st to the 5th cycle.

Leveraging the melt-processing capabilities of perovskite glass, the decommissioned devices that undergo fragmentation, interface delamination, or electrode detachment issues, should be principally compatible with the standard recycle procedure of traditional oxide glasses. Along this line, we have established a closed-loop recycling pathway for the perovskite devices as follows: (1) collection of end-of-life devices, (2) disassembly of the electrode and glass substrate, (3) melting and reshaping of perovskite glass, (4) device fabrication (Fig. 6a). This strategy does not require any toxic solvents with near-unity recycling efficiency, which meets with the requirements of environmental sustainability. In contrast, the recycling of traditional silicon-based devices primarily involves purifying wafers and etching metals using organic solvents and strong acids, followed by high-temperature smelting to produce silicon ingots^55,56^. This energy-consuming approach is also plagued by the residual dopants and impurities.

The structure and physical properties of the perovskite glass were examined across successive cycling. XRD analysis in Fig. 6b confirmed the typical amorphous structure of a-DPB-flux throughout five cycles. The organic moiety and local chemical environment are also preserved upon the melt-processing procedures as validated by the retained characteristic peaks of ^1^H NMR and FTIR spectra (Figs. 6c, d). Furthermore, we measured the optoelectronic performance of a-DPB-flux devices across multiple processing cycles. The resistivity and the *μτ* product, exhibit only minimal variation, remaining within the same order of magnitude (Fig. 6e, f, Supplementary Figs. 41and 42). Upon X-ray irradiation, the detection sensitivity remains largely unaffected after repeated cycling (Fig. 6g and Supplementary Fig. 43), suggestive of a promising candidate for reusable radiation detectors. It has been known that the unique orbital hybridization of metal halide semiconductors makes it tolerant to most of the defects^57,58^. Such unique defect chemistry may also contribute to the excellent recyclability of these materials.

## Discussion

The manifestation of flux-mediated approach circumvents the thermal budget issue, that stemmed from the deficit of the melting points relative to decomposition temperatures. By using trace amount of LiOH fluxes (<1.1 wt%), we demonstrate the wide applicability of this strategy in depressing the melting temperature of 8 metal halide systems by approximately 21–77 °C. Importantly, two non-meltable perovskites, e.g., BPI and DPI can be successfully melted and vitrified on the basis of this synthetic protocol. The reduction of processing temperature allows the successful fabrication of sustainable perovskite glass materials without notable loss in physical properties. Experimental and computational investigations reveal that the partial deprotonation reaction of hydroxide ions to ammonium protons would weaken the intermolecular interactions and disrupt the inorganic framework ordering, and thereby collectively contribute to *T*_m_ reduction. These semi-conductive perovskite glasses also adopt the advantages of melt-processability and integrability, that have been successfully integrated onto a TFT backplane for real-time high-resolution X-ray imaging. After multiple melt-processing cycles, the detection performance of these perovskite glasses has shown negligible degradation. We anticipate that the large diversification of the glassy material family derived from crystalline hybrid perovskites would realize sustainable applications in light detection, neuromorphic computing, catalysis and energy conversion devices, and may also offer abundant unique materials for fundamental researches in condensed matter physics.

## Methods

### Materials

Lead bromide (PbBr_2_, 99%), lead iodide (PbI_2_, 99%), bismuth oxide (Bi_2_O_3_, 99%), 1,1-dimethylbiguanide (DGA, 99%), (S)-(-)-1-(1-naphthyl)ethylamine (S-NEA, 99%), (R)-( + )-1-(1-naphthyl)ethylamine (R-NEA, 99%), 4-(ethylaminomethyl)pyridine (4-EAMP, 99%), butylamine (BA, 99%), hydrobromic acid (HBr, 33 wt% solution in acetic acid) and hydroiodic acid (HI, 57 wt% in water, with 1.5% H_3_PO_2_) were purchased from Shanghai Macklin Biochemical Technology Co. Lithium iodide (LiI, 99%), sodium iodide (NaI, 99%), potassium iodide (KI, 99%), lithium bromide (LiBr, 99%), sodium bromide (NaBr, 99%), potassium bromide (KBr, 99%), lithium chloride (LiCl, 99%), sodium chloride (NaCl, 99%), potassium chloride (KCl, 99%), lithium fluoride (LiF, 99%) sodium fluoride (NaF, 99%), potassium fluoride (KF, 99%), lithium sulfate (Li_2_SO_4_, 99%), sodium sulfate (Na_2_SO_4_, 99%), potassium sulfate (K_2_SO_4_, 99%), lithium nitrate (LiNO_3_, 99%), sodium nitrate (NaNO_3_, 99%), potassium nitrate (KNO_3_, 99%), lithium carbonate (Li_2_CO_3_, 99%), sodium carbonate (Na_2_CO_3_, 99%), potassium carbonate (K_2_CO_3_, 99%), lithium hydroxide (LiOH, 99%), sodium hydroxide (NaOH, 99%) and potassium hydroxide (KOH, 99%) were purchased from Shanghai Aladdin Biochemical Technology Co. All reagents were used directly without further purification.

### Synthesis of DGABr_2_

DGA (77.5 mmol) and absolute ethyl alcohol (5 ml) were mixed in a 50 ml round-bottom flask under stirring. After cooling in an ice bath, 32 ml of HBr solution was added and reacted for 9 h, followed by rotary evaporation at 60 °C. The obtained solid products were recrystallized using anhydrous diethyl ether, and finally dried at 60 °C in a vacuum oven for 24 h.

### Synthesis of DGAI_2_

DGA (77.5 mmol) and absolute ethyl alcohol (5 ml) were mixed in a 50 ml round-bottom flask under stirring. After cooling in an ice bath, 21 ml of HI solution was added and reacted for 9 h, followed by rotary evaporation at 60 °C. The obtained solid products were recrystallized using anhydrous diethyl ether, and finally dried at 60 °C in a vacuum oven for 24 h.

### Synthesis of (DGA)PbBr_4_ single crystals

To grow (DGA)PbBr_4_ crystals, 3.67 g (10 mmol) PbBr_2_ and 2.89 g (10 mmol) DGABr_2_ were dissolved in HBr (35 ml). The mixture was heated to 160 °C under stirring for 30 min to form a pale yellow transparent solution. After that, the hot solution was slowly cooled to room temperature at a rate of 5 °C min^−1^ to yield pale yellow crystals.

### Synthesis of (DGA)PbI_4_ single crystals

To grow (DGA)PbI_4_, 4.61 g (10 mmol) PbI_2_ and 3.83 g (10 mmol) DGAI_2_ were dissolved in HI (30 ml). The solution was heated at 150 °C with stirring at 500 rpm for 30 min to form a brownish-yellow transparent solution. Subsequent slow cooling to room temperature at a rate of 5 °C min^−1^ to yield brownish-yellow block-shaped crystals.

### Synthesis of (DGA)_2_Bi_2_Br_10_·4H_2_O single crystals

To grow (DGA)_2_Bi_2_Br_10_·4H_2_O, 2.33 g (5 mmol) Bi_2_O_3_ and 2.89 g (10 mmol) DGABr_2_ were dissolved in HBr (25 ml). The mixture was heated at 150 °C with continuous stirring at 500 rpm for 30 min, resulting in a yellow transparent solution. Subsequent slow cooling to room temperature at a rate of 5 °C min^−1^ produced light yellow block-shaped crystals.

### Synthesis of (DGA)_2_Bi_2_I_10_·2H_2_O single crystals

To grow (DGA)_2_Bi_2_I_10_·2H_2_O, 2.33 g (5 mmol) Bi_2_O_3_ and 3.83 g (10 mmol) DGAI_2_ were dissolved in HI (30 ml). The solution was heated at 160 °C with stirring at 500 rpm for 30 min until a dark red transparent solution formed. Subsequent slow cooling to room temperature at a rate of 5 °C min^−1^ yielded dark red block-shaped crystals.

### Synthesis of (S-NEA)_2_PbBr_4_ and (R-NEA)_2_PbBr_4_ single crystals

To grow (S-NEA)_2_PbBr_4_ and (R-NEA)_2_PbBr_4_, 90 mg (0.24 mmol) PbBr_2_ and 78 μl (0.48 mmol) S-NEA were dissolved in aq. HBr (1 ml) and deionized water (2.4 ml) in a sealed vial at 95 °C. The hot solution was slowly cooled to room temperature over a period of 24 h in a water bath, resulting in the formation of colorless plate-like (S-NEA)_2_PbBr_4_ single crystals. The (R-NEA)_2_PbBr_4_ crystals were grown using the same recipe used for (S-NEA)_2_PbBr_4_, with R-NEA substituted as the ammonium group precursor.

### Synthesis of (BA)_2_PbI_4_ single crystals

To grow (BA)_2_PbI_4_, 2.305 g (5 mmol) PbI_2_ and 0.732 g (10 mmol) BA were dissolved in HI (20 ml). The mixture was placed on a heated stirring platform at 160°C and stirred at 500 rpm for 30 min until a clear brownish-yellow solution was obtained. The hot solution was slowly cooled to room temperature at a rate of 5 °C min^−1^ to yield brownish-yellow plate-like crystals. The resulting (BA)_2_PbI_4_ crystals were collected by filtration, washed with diethyl ether, and dried in a vacuum oven at 60 °C for 24 h.

### Preparation of amorphous glasses

The glass samples were prepared via a direct melt-quenching strategy. First, (DGA)PbBr_4_ crystals and LiOH powder were mixed in a mass ratio of 91:1 and finely grounded for 5 min. The homogeneous mixture was transferred to a quartz crucible and heated in a muffle furnace at 230 °C for 30 min to form a uniform melt. Subsequently, the melt was poured onto an aluminum plate and allowed to cool naturally to room temperature, yielding transparent glass. For the undoped group, undoped samples (without LiOH addition) were prepared under identical procedures but required a higher heating temperature of 270 °C.

Other perovskite glass samples in this study were prepared using identical synthetic protocols with adjusted LiOH doping ratios and processing temperatures. For (DGA)_2_Bi_2_Br_10_·4H_2_O, (DGA)_2_Bi_2_I_10_·2H_2_O, (4-EAMP)_2_Pb_3_Br_10_, (S-NEA)_2_PbBr_4_ and (R-NEA)_2_PbBr_4_, the LiOH incorporation mass ratios were 107:1, 138:1, 235:1 and 121:1, respectively, with corresponding processing temperatures of 220 °C, 210 °C, 190 °C and 170 °C. The processing temperatures of samples without LiOH addition were 250 °C, 260 °C, 230 °C and 200 °C, respectively. For (BA)_2_PbI_4_ and (DAG)PbI_4_, the LiOH mass ratios were 90:1 and 117:1, respectively, and the processing temperatures were 240 °C and 230 °C, respectively. The bulk glasses of (BA)_2_PbI_4_ and (DAG)PbI_4_ were obtained by heating and melting the materials in a vacuum-sealed quartz glass tube, followed by rapid quenching in liquid nitrogen.

### Material characterization

Single-crystal X-ray diffraction (SCXRD) measurements were performed on a Bruker Smart Apex CCD system at 100 K using Mo K*α* radiation (λ = 0.71073 Å). Powder X-ray diffraction (PXRD) patterns were recorded on a Bruker Advance D8 X-ray diffractometer equipped with Cu K*α* radiation (40 kV, 40 mA). Thermogravimetric analysis (TGA) was conducted using a Netzsch STA 449 F3 instrument under nitrogen flow (50 ml min^−1^), with samples heated from room temperature to 600 °C at a constant heating rate of 10 °C min^−1^. Differential scanning calorimetry (DSC) measurements were performed on a Netzsch DSC 404 F3 instrument under identical temperature conditions and gas flow parameters, with an aluminum crucible equipped with ventilation holes. Combined TGA-DSC analysis was carried out using a Netzsch STA 449 F3 instrument, with data acquisition spanning 30–600 °C at a heating rate of 10 °C min^−1^ under nitrogen atmosphere. UV-vis transmittance spectra of the glass samples were obtained using a Jinda UV-2600 spectrophotometer operating in the wavelength range of 200–800 nm. The structure of the single crystals was observed by an LW150PT optical microscope equipped with crossed polarizers. Transmission electron microscopy (TEM) characterization was performed using a Thermo Fisher Talos F200X microscope operated at 200 kV acceleration voltage. High-angle annular dark-field scanning transmission electron microscopy (HAADF-STEM) images were acquired with a convergence semi-angle of 11 mrad and collection angles of 59 and 200 mrad. Energy-dispersive X-ray spectroscopy (EDX) mapping was conducted using four in-column Super-X detectors. Field-emission scanning electron microscopy (FE-SEM) images were collected on a Hitachi S-4800 microscope operated at 5 kV. ^1^H nuclear magnetic resonance (NMR) spectra were recorded at 298 K on a Bruker Avance III HD 400 MHz spectrometer using tetramethylsilane (TMS) as an internal reference. Solid-state NMR experiments were performed on a Bruker Avance III 600 MHz spectrometer with magic-angle spinning at 8 kHz using a 4 mm zirconia rotor. X-ray photoelectron spectroscopy (XPS) measurements were conducted on a Thermo Scientific ESCALAB 250Xi system with monochromatic Al K*α* radiation (*hν* = 1486.6 eV). Synchrotron-based X-ray absorption spectroscopy measurements at the Pb L_3_-edge were performed at the 1W1B beamline of the Shanghai Synchrotron Radiation Facility (BSRF), collecting both extended X-ray absorption fine structure (EXAFS) and X-ray absorption near-edge structure (XANES) data at ambient temperature. Raman spectra were acquired using a Renishaw in Via Reflex confocal Raman microscope with 532 nm laser excitation. Fourier transform infrared (FTIR) spectra were recorded on a PerkinElmer Frontier spectrometer using KBr pellet method.

### X-ray detection measurement and imaging

The single-pixel X-ray detector was fabricated by using a (DGA)PbBr_4_ glass sheet (thickness: 0.2 mm) with an Ag/(DGA)PbBr_4_ glass/Ag structure. Ag electrodes (100 nm thick) were deposited on both sides via thermal evaporation. The effective device area of the X-ray detector was 0.01 cm^2^. During the X-ray detection experiment, the glass sample was exposed to an X-ray tube (Cu anode, Canon, A40) with a tube voltage of 40 kV. The dose rates were controlled by adjusting the tube current (2–40 mA) or using aluminum foil as attenuation layer, which was calibrated by a gas ionization chamber (PTW 34013). The X-ray response current was measured using a Keithley 2400 digital source meter. The sensitivity (*S*) of the X-ray detector, defined as the response capability to the dose rate per unit area, can be calculated using the following formula:

1 $$S=\frac{{I}_{{{{\rm{p}}}}}-{I}_{{{{\rm{d}}}}}}{{DA}}$$

where *I*_p_ is the photocurrent, *I*_d_ is the dark current, *D* is the dose rate, and *A* is the detection area^59,60^.

For X-ray imaging, a 3.3 × 3.3 cm size (DGA)PbBr_4_ glass was integrated onto the TFT substrate backplane by hot-pouring metho. The TFT backplane and readout circuits were provided by LinkZill. Specifically, the (DGA)PbBr_4_ melt was poured and pressed on the TFT backplane to form an intimate contact at the interface. Finally, the top surface of the device was covered with 100 nm of silver as the top electrode. The flat-panel detector was operated under a 5.32 μGy_air_ s^−1^ dose rate with 15 V bias for imaging. The sample size of the pixel value distribution in the detector was 4096, and each pixel had a grayscale value between 0 and 255.

### Photoconductivity analysis

For *µτ* product measurement, a 405 nm LED as excitation light, was modulated at 50 Hz by a function generator. The photoconductivity current of the glass sample was recorded by using a Keithley 2400 digital source meter. A modified Hecht equation was used to fit the current-voltage curve, yielding a product (*µτ*) and a surface recombination velocity (*s*):

2 $$I=\frac{{I}_{0}\mu \tau V}{{L}^{2}}\frac{1-\exp (-\frac{{L}^{2}}{\mu \tau V})}{1+\frac{L}{V}\,\frac{s}{\mu }}$$

where *I*_0_ is the saturated photocurrent, *L* is the thickness, *V* is the applied bias, and *τ* is the carrier lifetime^61^.

### Theoretical calculation methods

Density functional theory (DFT) and Gaussian 09 were used to calculate the molecular structure^62–65^. The quantum calculations for geometry optimizations and vibrations were carried out at the B3LYP/LANL2DZ level of theory. Global optimization with frequency calculation was performed using the 6–31 g basis. The isosurface maps and structure were visualized by the Gaussview 06 package and visual molecular dynamics. The kinetic cut-off energy was set to 450 eV and coupled with the projector-augmented wave method. Also, the structure optimizations adopted Monkhorst–Pack 1 × 1 × 1 k-grids. All the atoms were allowed to relax and the convergence criteria for the total forces and EDIFFG were set as −0.05. The formation energy *E*_f_(*X*) is obtained from perovskite models with different deprotonation degrees. The total energy *E*(*X*) of the substrate system is calculated by DFT, *n*_*i*_ is the number of atoms of *i* (Pb, N, C, etc) in the substrate and *μ*_*i*_ is the chemical potential of atom *i*. The formation energy is defined as follows:

3 $${E}_{f}\left(X\right)=E\left(X\right)-{\varSigma }_{i}{n}_{i}{\mu }_{i}$$

## Supplementary information

Supplementary Information

Transparent Peer Review file

## Source data

Source Data

## Data Availability

Source data are provided with this paper. The data within the Supplementary Information are available from the corresponding authors. The crystal structure reported in this article has been uploaded to the Cambridge Crystallographic Data Center (CCDC). The deposition numbers are 2545763, 2545764, and 2545765. Source data are provided with this paper.
