# Supplementary material for: Deprotonation-mediated vitrification of organic-inorganic hybrid perovskites
Source: Nat Commun. 2026 Aug 17;17:9872. doi: 10.1038/s41467-026-76712-z (PMC13578220; doi:10.1038/s41467-026-76712-z)
Supplement: Supplementary file 1 [file 41467_2026_76712_MOESM1_ESM.pdf]

Supplementary Information

Deprotonation-mediated vitrification of organic-inorganic hybrid perovskites

Peng Wang[1], Guocan Chen[1], Yuchen Zhu[1], Zejiang Huang[2], Zhanpeng Wei[1], Da Liu[1], Sihan Zeng[1], Qing Li[1], Yuhao Zhao[1], Haiyang Yuan[1], Xue Lu Wang[2], Yu Hou[1]*, Hua Gui Yang[1]* and Shuang Yang[1]*

[1]Key Laboratory for Ultrafine Materials of Ministry of Education, Shanghai Engineering Research Center of Hierarchical Nanomaterials, School of Materials Science and Engineering, East China University of Science and Technology, 130 Meilong Road, Shanghai 200237, China.

[2]Physics Department & Shanghai Key Laboratory of Magnetic Resonance, School of Physics and Electronic Science, East China Normal University, North Zhongshan Road 3663, Shanghai 200062, China.

*Correspondence: yhou@ecust.edu.cn (Y.H.), hgyang@ecust.edu.cn (H.G.Y.), syang@ecust.edu.cn (S.Y.)

Name definition: Uniformly abbreviate the names of perovskite materials mentioned in the text. For example, $(DGA)PbBr_4$ is abbreviated as DPB. Crystal and amorphous glass are defined as DPB and a-DPB respectively, and the same is true for other materials in the text. The presence and absence of LiOH are represented by w/ flux and w/o flux respectively.

Supplementary Table 1. The mass ratio (wt%) of the flux for all samples in Figure 1c.

	OH^-	CO_3^{2-}	NO_3^-	SO_4^{2-}	F^-	Cl^-	Br^-	I^-
Li^+	1.1	3.38	3.15	5.03	1.19	1.94	3.97	6.12
Na^+	1.83	4.85	3.89	6.49	1.92	2.67	4.71	6.86
K^+	2.57	6.32	4.62	7.97	2.66	3.41	5.44	7.59

Supplementary Table 2. Crystal data and structure refinement of (DGA)$PbBr_4$.

Empirical formula	$C_4H_{13}N_5PbBr_4$
Formula weight	658.02
Temperature/K	100.00
Crystal system	monoclinic
Space group	$P2_1/n$
a/Å	6.1724(2)
b/Å	11.7273(4)
c/Å	19.6302(8)
α/°	90
β/°	97.9480(10)
γ/°	90
Volume/Å^3	1407.29(9)
Z	4
ρ_{calc}g/cm^3	3.106
μ/mm^{-1}	23.322
F (000)	1176.0
Crystal size/mm^3	0.15 × 0.13 × 0.12
Radiation	MoKα (λ = 0.71073)
2θ range for data collection/°	5.444 to 52.974
Index ranges	-6 ⩽ h ⩽ 7, -14 ⩽ k ⩽ 14, -24 ⩽ l ⩽ 24
Reflections collected	15848
Independent reflections	2916 [R_{int} = 0.0677, R_{sigma} = 0.0482]
Data/restraints/parameters	2916/0/129
Goodness-of-fit on F^2	1.045
Final R indexes [I>=2σ (I)]	R_1 = 0.0284, wR_2 = 0.0685
Final R indexes [all data]	R_1 = 0.0321, wR_2 = 0.0706
Largest diff. peak/hole / e Å^{-3}	0.85/-1.73

Supplementary Table 3. Crystal data and structure refinement of $(DGA)_2Bi_2Br_{10}\cdot 4H_2O$.

Empirical formula	$C_8H_{34}Bi_2Br_{10}N_{10}O_4$
Formula weight	1551.51
Temperature/K	100.00
Crystal system	triclinic
Space group	*P-1*
a/Å	9.2365(3)
b/Å	9.6080(4)
c/Å	10.7047(4)
α/°	81.2250(10)
β/°	77.2480(10)
γ/°	70.2820(10)
Volume/Å^3	869.06(6)
Z	1
ρ_{calc}g/cm^3	2.965
μ/mm^{-1}	21.637
F (000)	700.0
Crystal size/mm^3	0.12 × 0.11 × 0.1
Radiation	MoKα (λ = 0.71073)
2θ range for data collection/°	4.764 to 52.882
Index ranges	$-10 \leqslant h \leqslant 11$, $-12 \leqslant k \leqslant 12$, $-13 \leqslant l \leqslant 13$
Reflections collected	9144
Independent reflections	3557 [R_{int} = 0.0565, R_{sigma} = 0.0660]
Data/restraints/parameters	3557/0/162
Goodness-of-fit on F^2	1.039
Final R indexes [I>=2σ (I)]	R_1 = 0.0333, wR_2 = 0.0732
Final R indexes [all data]	R_1 = 0.0396, wR_2 = 0.0765
Largest diff. peak/hole / e Å^{-3}	1.07/-1.69

Supplementary Table 4. Crystal data and structure refinement of $(DGA)_2Bi_2I_{10}\cdot 2H_2O$.

Empirical formula	$C_8H_{30}Bi_2I_{10}N_{10}O_2$
Formula weight	1985.38
Temperature/K	100.00
Crystal system	monoclinic
Space group	$P2_1/n$
a/Å	11.756(3)
b/Å	10.779(3)
c/Å	15.598(4)
α/°	90
β/°	102.357(9)
γ/°	90
Volume/Å^3	1930.7(8)
Z	2
ρ_{calc}g/cm^3	3.415
μ/mm^{-1}	17.120
F (000)	1720.0
Crystal size/mm^3	0.13 × 0.12 × 0.1
Radiation	MoKα (λ = 0.71073)
2θ range for data collection/°	4.63 to 52.89
Index ranges	-13 ⩽ h ⩽ 14, -13 ⩽ k ⩽ 13, -19 ⩽ l ⩽ 19
Reflections collected	15043
Independent reflections	3966 [R_{int} = 0.0622, R_{sigma} = 0.0568]
Data/restraints/parameters	3966/0/147
Goodness-of-fit on F^2	1.032
Final R indexes [I>=2σ (I)]	R_1 = 0.0300, wR_2 = 0.0613
Final R indexes [all data]	R_1 = 0.0378, wR_2 = 0.0647
Largest diff. peak/hole / e Å^{-3}	1.29/-1.60

Supplementary Table 5. The calculation results of ΔH_{fus} and ΔS_{fus} values of DPB and DPB-flux.

**Materials**	**ΔH_{fus}**	**ΔS_{fus}**
DPB	15.92 KJ mol^{-1}	29.95 J mol^{-1} K^{-1}
DPB-flux	14.82 KJ mol^{-1}	30.06 J mol^{-1} K^{-1}

Supplementary Table 6. Summary of key performance parameters of representative perovskite-based X-ray detectors.

Type	Dimension	Electric field ($V\ cm^{-1}$)	Sensitivity ($\mu C\ Gy^{-1}\ cm^{-2}$)	LoD ($nGy\ s^{-1}$)	Ref
$(NH_4)_3Bi_2I_9$	2D	22 (parallel)	8200	210	(1)
$CsPbBr_3$	3D	4000	42000	136	(2)
$MAPbBr_3$	3D	4670	21000	100	(3)
$FAPbI_3$	3D	1000	216000	2	(4)
$Cs_2AgBiBr_6$	3D	250	105	59.7	(5)
$MAPbI_3$+$PEA_2MA_8Pb_9I_{28}$	3D	333	19500	480	(6)
$PEA_2MA_8Pb_9I_{28}$	2D	6,000	10860	69	(7)
$(3AP)PbCl_4$	2D	2500	792	1540	(8)
$(F\text{-}PEA)_2PbI_4$	2D	1333	3402	23	(9)
$(4\text{-}AP)CsPb_2I_8$	2D	500	1890	529	(10)
$CsPb_2Br_5$	2D	6250	8865.6	12.7	(11)
$(BPEA)_2PbI_4$	2D	800	1003	366	(12)
$(4\text{-}AP)_3AgBi_3Br_{20}$	1D	500 (parallel)	1567	38.1	(13)
$(C_4H_{14}N_2S_2)_6Pb_5I_{22}\ 4H_2O$	1D	500	142000	11.7	(14)
$Cs_3Cu_2I_5$	0D	125	3433	33.17	(15)
$(Gua)_3Bi_2I_9$	0D	8333	18.23	237.54	(16)
α-Se	amorphous	$0.5–1\times10^5$	~20–50	~5500	(17-18)
CdZnTe	/	2500	2400	50000	(19)
$MTP_3Bi_2I_9$	glass	4000	1600	19.69	(20)
$(4\text{-}EAMP)_2Pb_3Br_{10}$	glass	1000	8581.7	135	(21)
a-DPB-flux	glass	5000	35674.1	93.6	This work

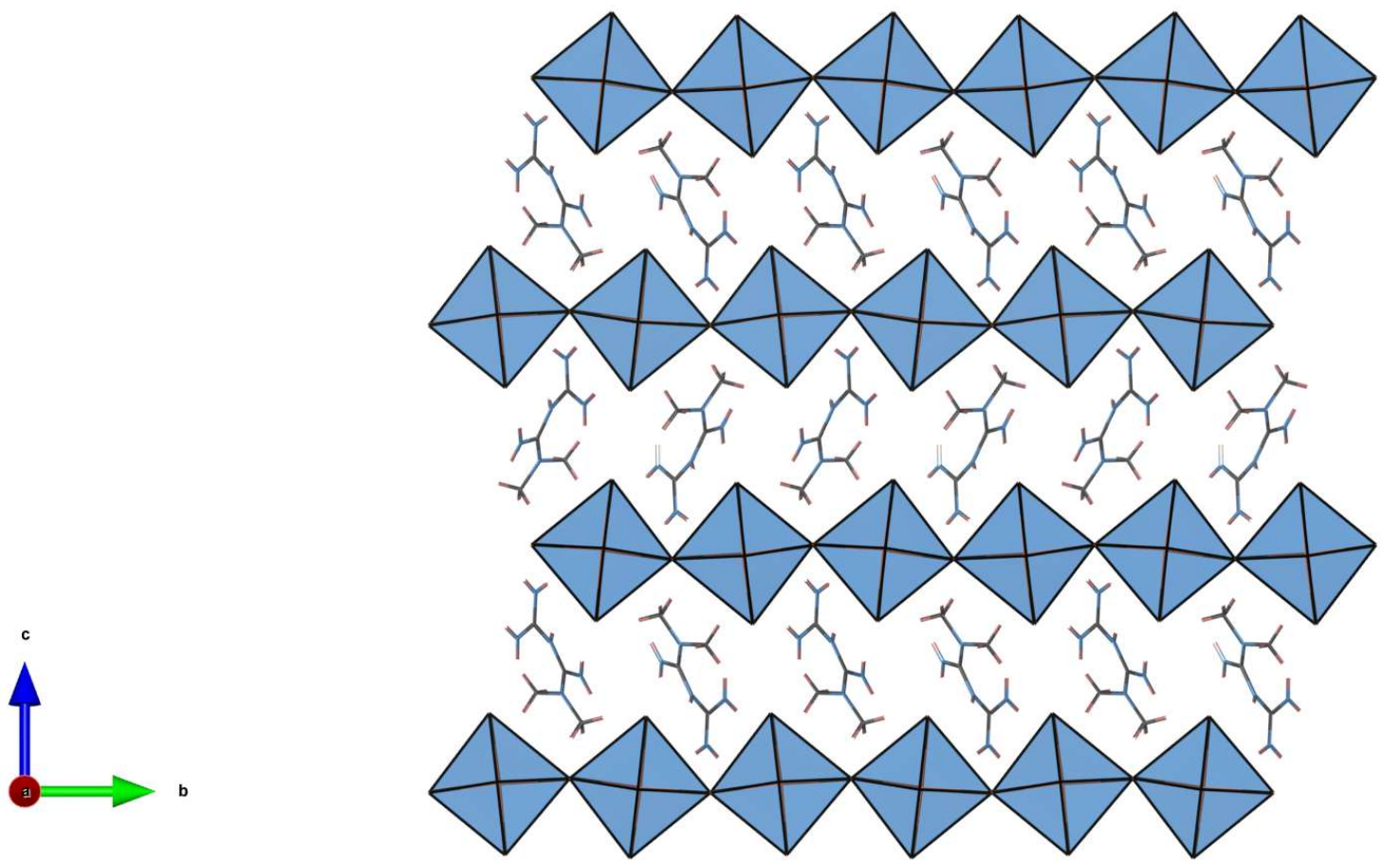

Supplementary Figure 1. Single crystal structure diagram of DPB. The compound crystallizes in the monoclinic space group *P2₁/n*, and the asymmetric unit consists of four organic molecules and corner-sharing [$PbBr_6$] octahedron units. This structure is equivalent to a DJ phase of 2D perovskites.

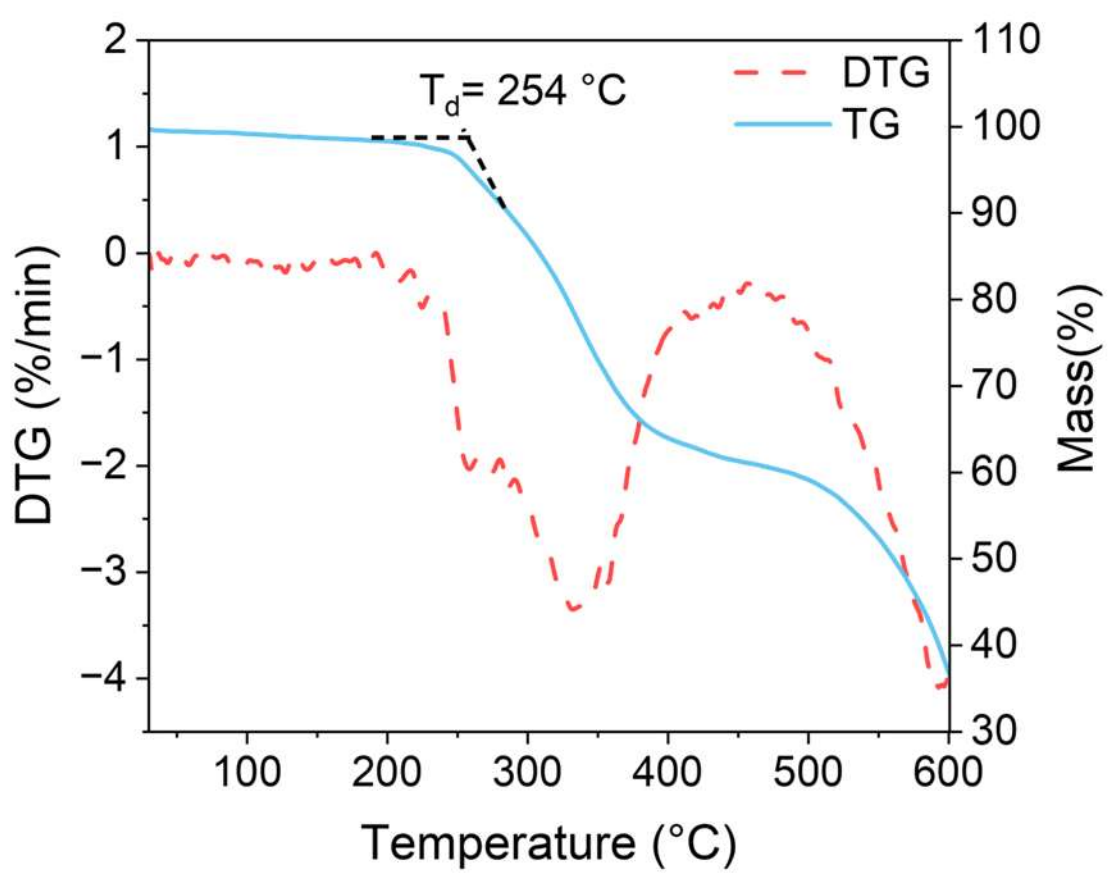

Supplementary Figure 2. TGA curves of DPB with a standard 10 °C min^{-1} ramping rate. The decomposition temperature of DPB was determined to be 254 °C.

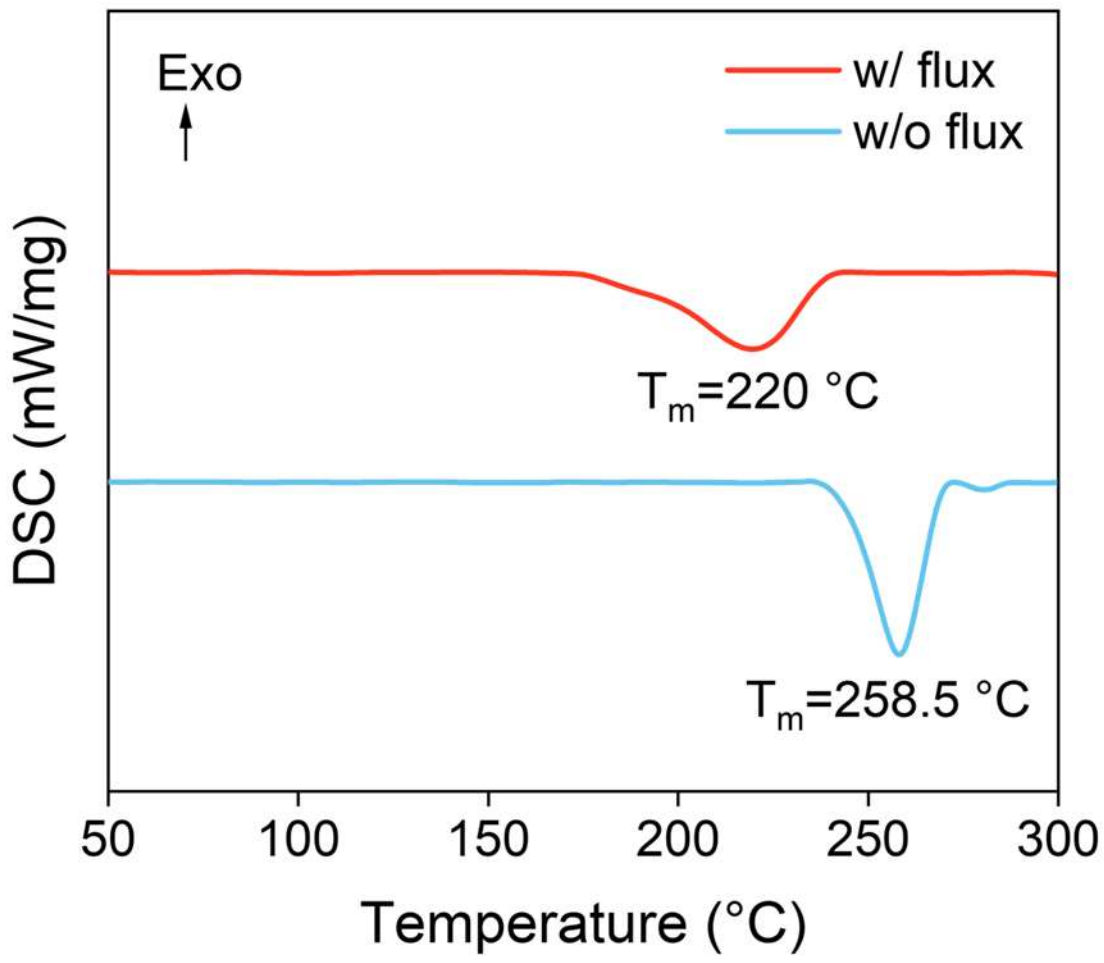

Supplementary Figure 3. DSC curves of DPB and DPB-flux with a standard 10 °C min^{-1} ramping rate. The melting point was taken as the endothermic peak temperature. After LiOH doping, the melting temperature reduced from 258.5 °C for undoped to 220 °C, showing a T_m reduction of 38.5 °C.

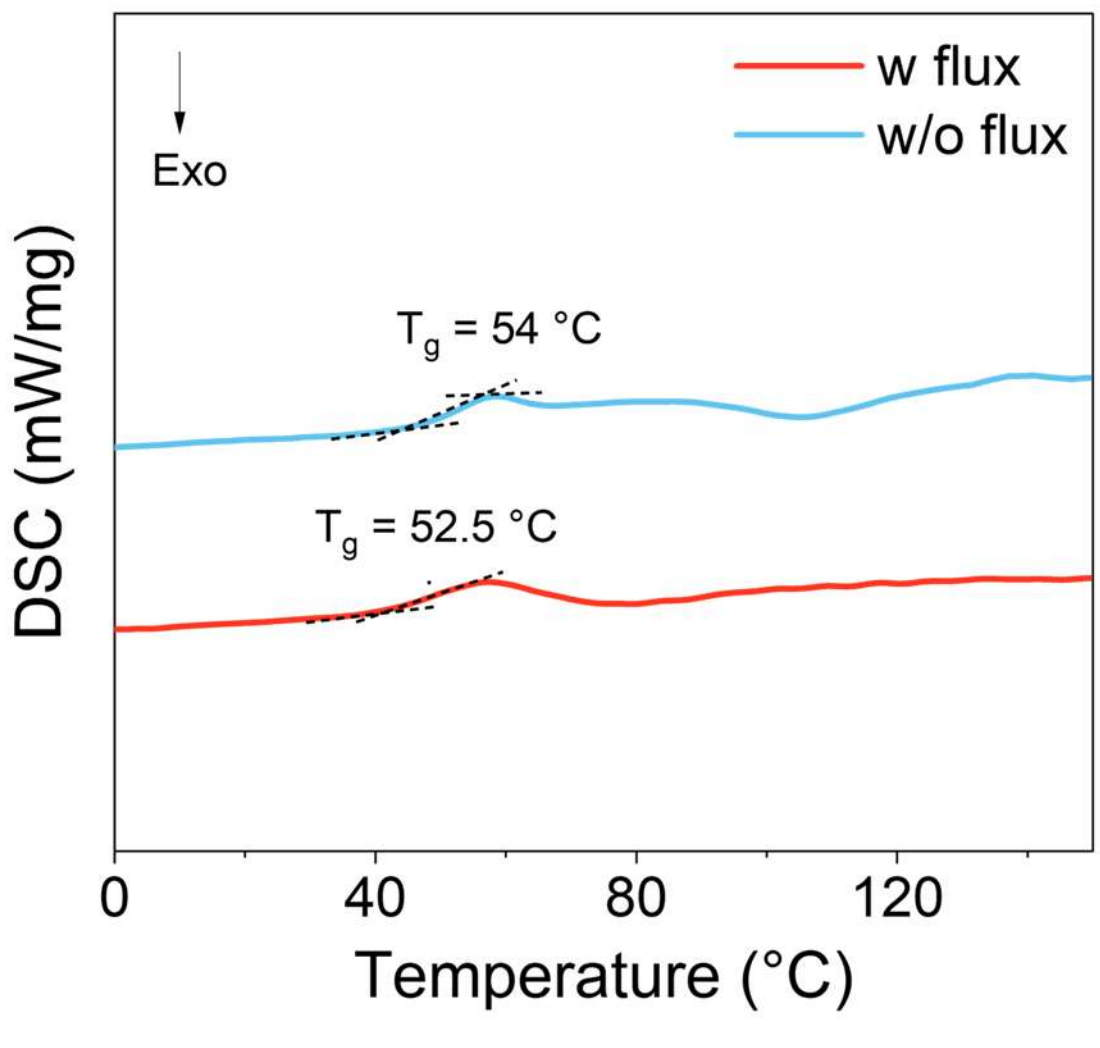

Supplementary Figure 4. DSC curves of a-DPB and a-DPB-flux with a standard 10 °C min^{-1} ramping rate. The T_g values were taken as the endothermic peak midpoint. Upon LiOH doping, T_g decreased from 54 °C to 52.5 °C.

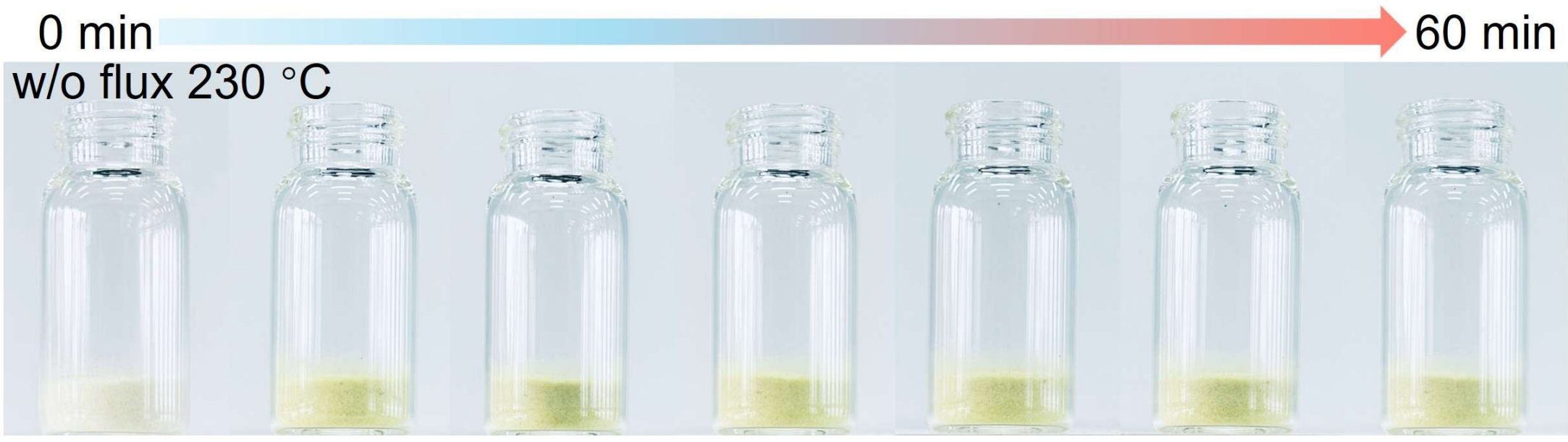

Supplementary Figure 5. DPB powder at 230 °C for various time intervals. No melting behavior can be observed after one hour of heating.

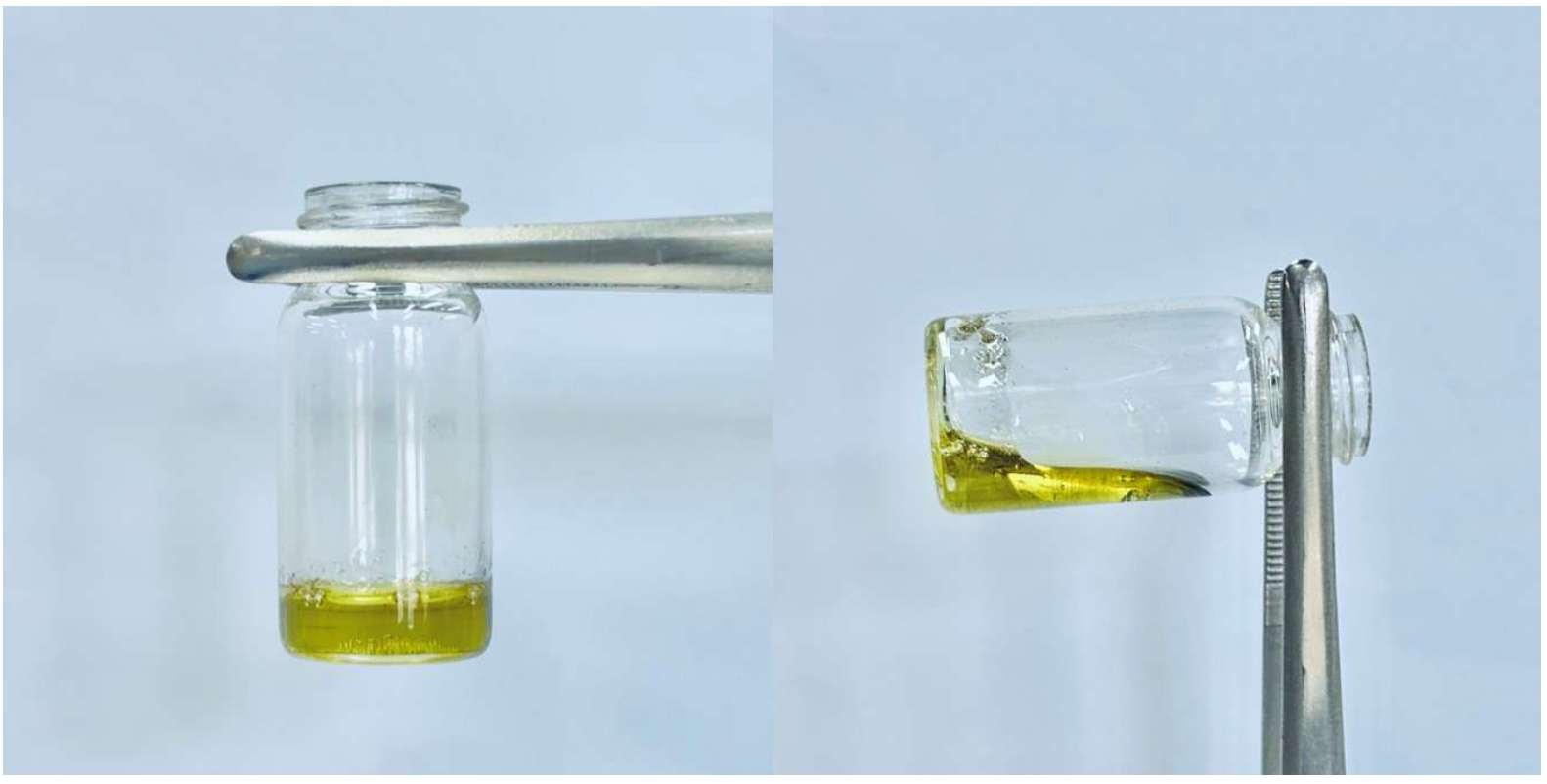

Supplementary Figure 6. Pictures of melted DPB-flux. The doped melt demonstrated certain fluidity, which can be shaped into desired structure.

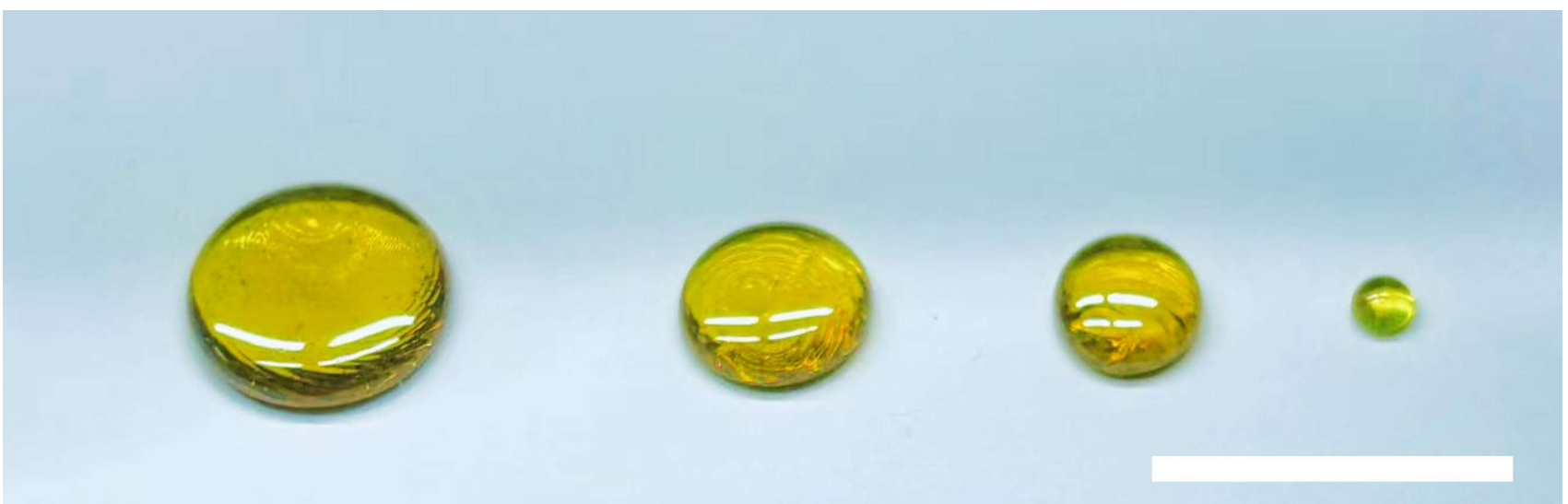

Supplementary Figure 7. a-DPB-flux with LiOH flux showing controlled dimensions and optical transparency (scale bars: 2 cm).

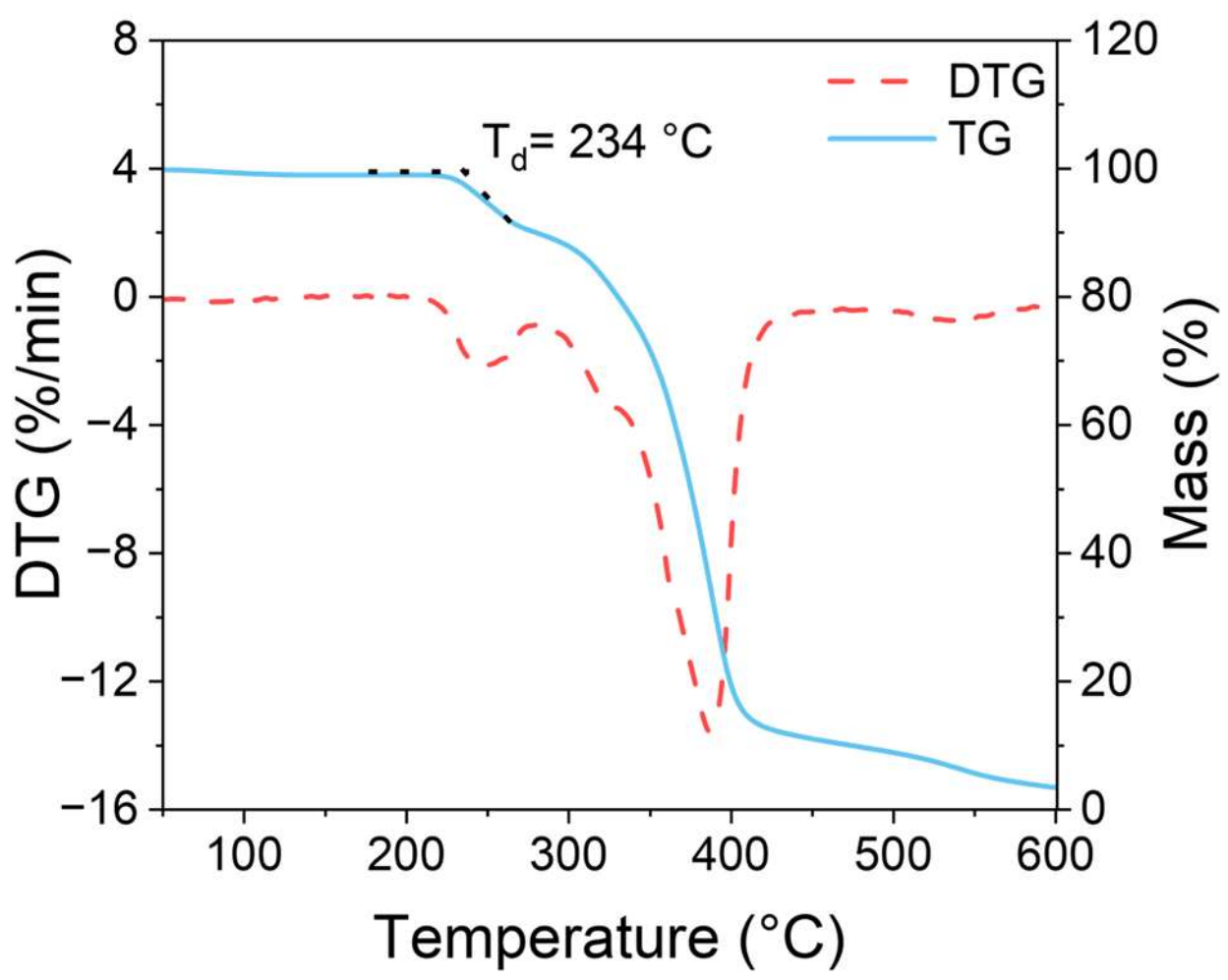

Supplementary Figure 8. TGA curves of DBI with a standard 10 °C min^{-1} ramping rate. The T_d value is estimated to be about 234 °C.

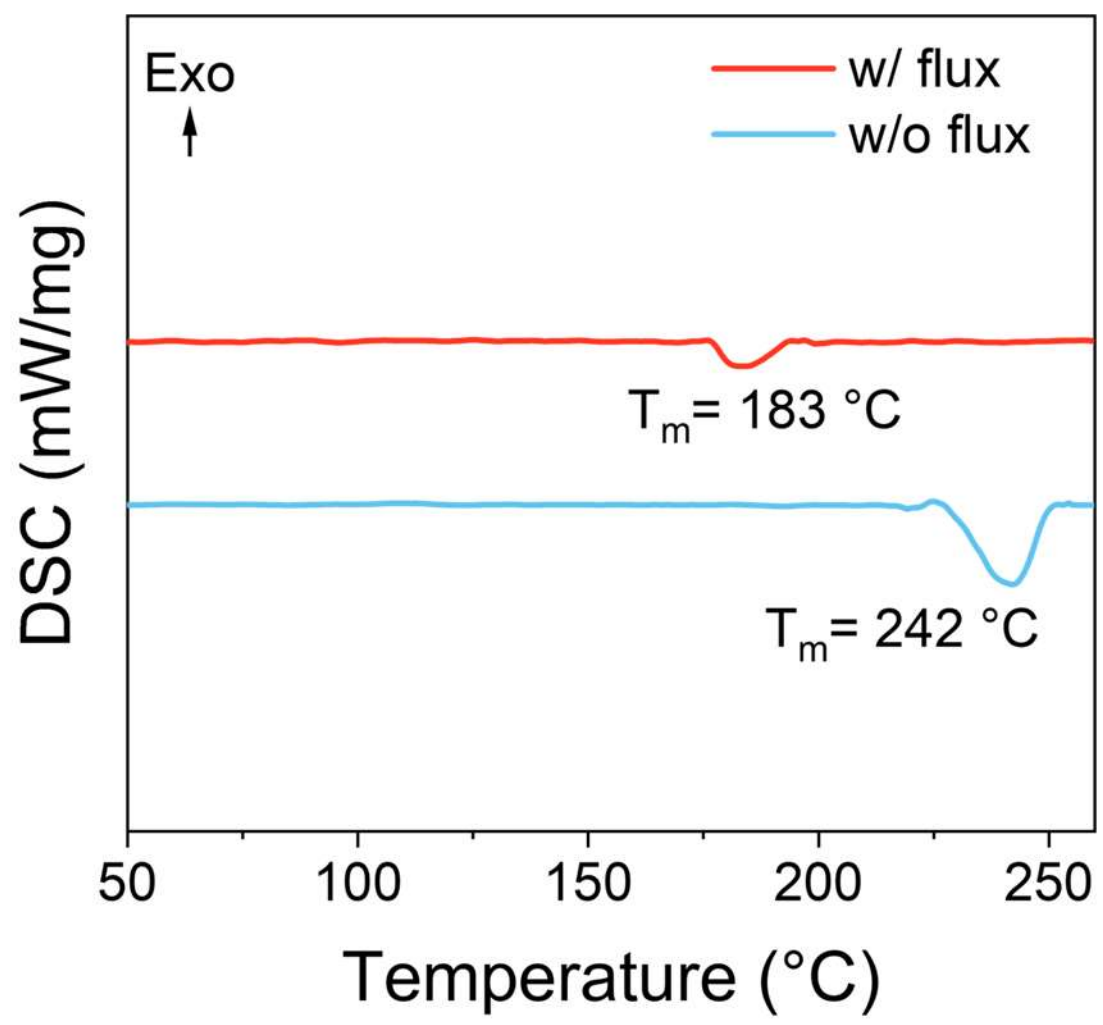

Supplementary Figure 9. DSC curves of DBI and DBI-flux with a standard 10 °C min^{-1} ramping rate. The T_m reduces from 242 °C to 183 °C after doping.

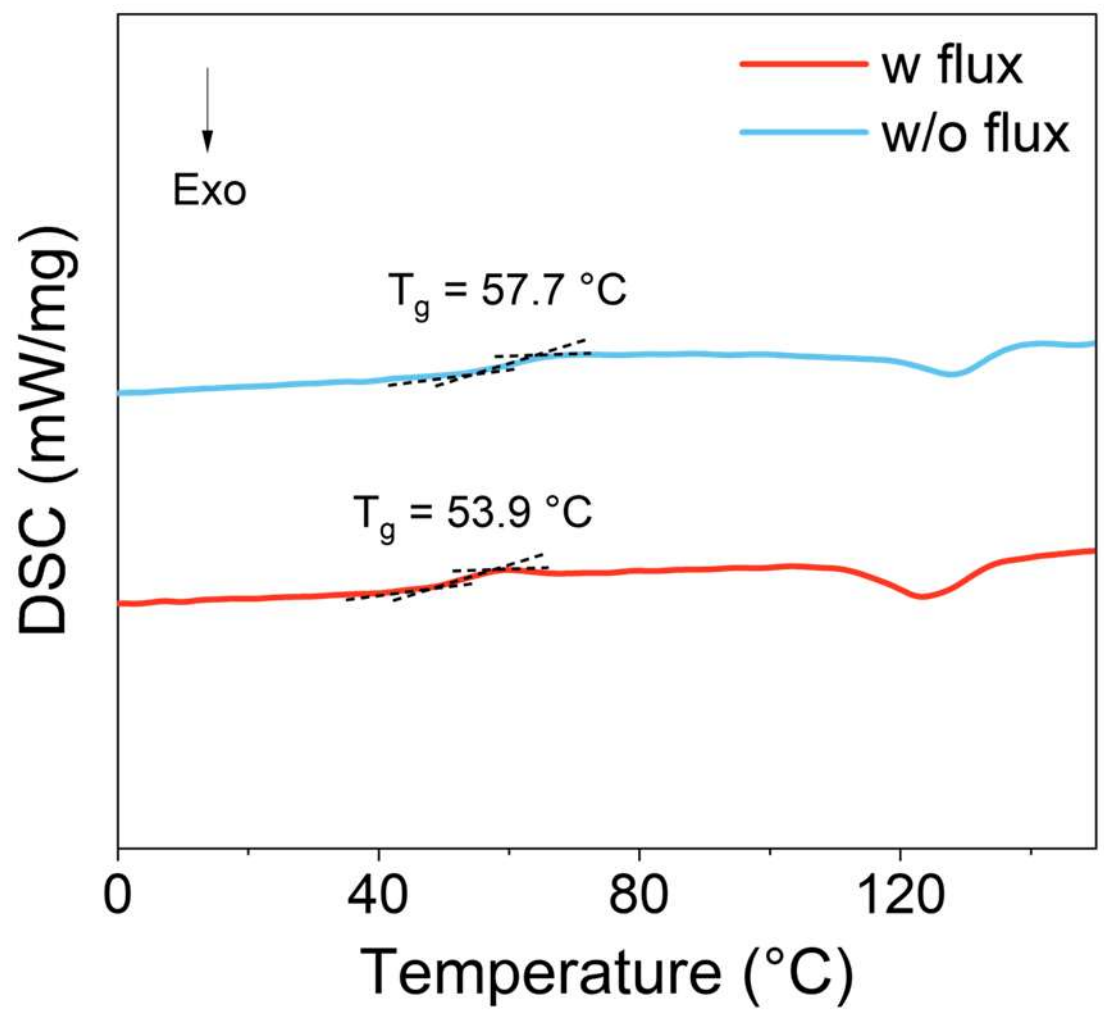

Supplementary Figure 10. DSC curves of a-DBI and a-DBI-flux with a standard 10 °C min^{-1} ramping rate. The T_g value is reduced from 57.7 °C to 53.9 °C upon the addition of LiOH.

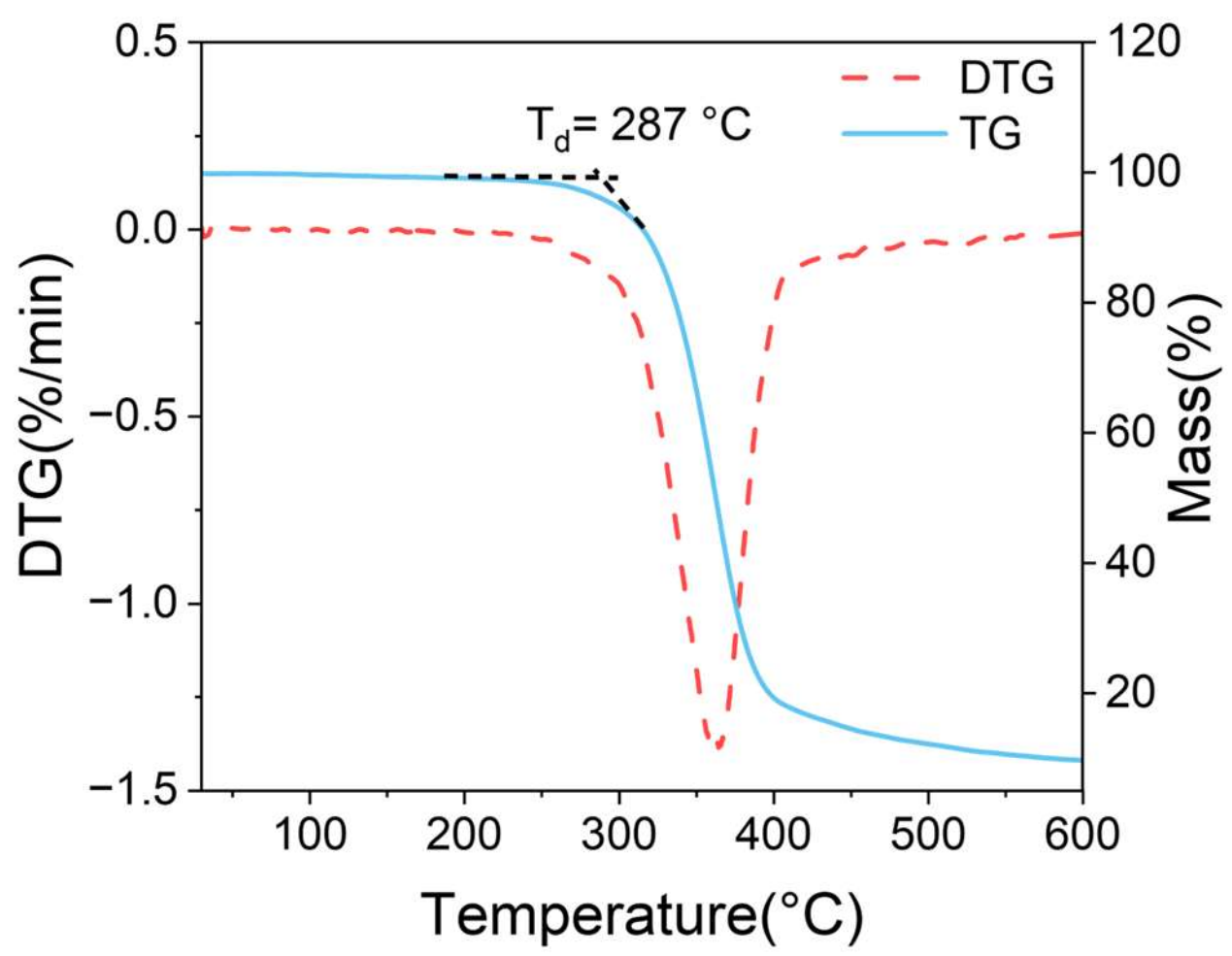

Supplementary Figure 11. TGA curves of DBB with a standard 10 °C min^{-1} ramping rate. The T_d value is approximately 287 °C.

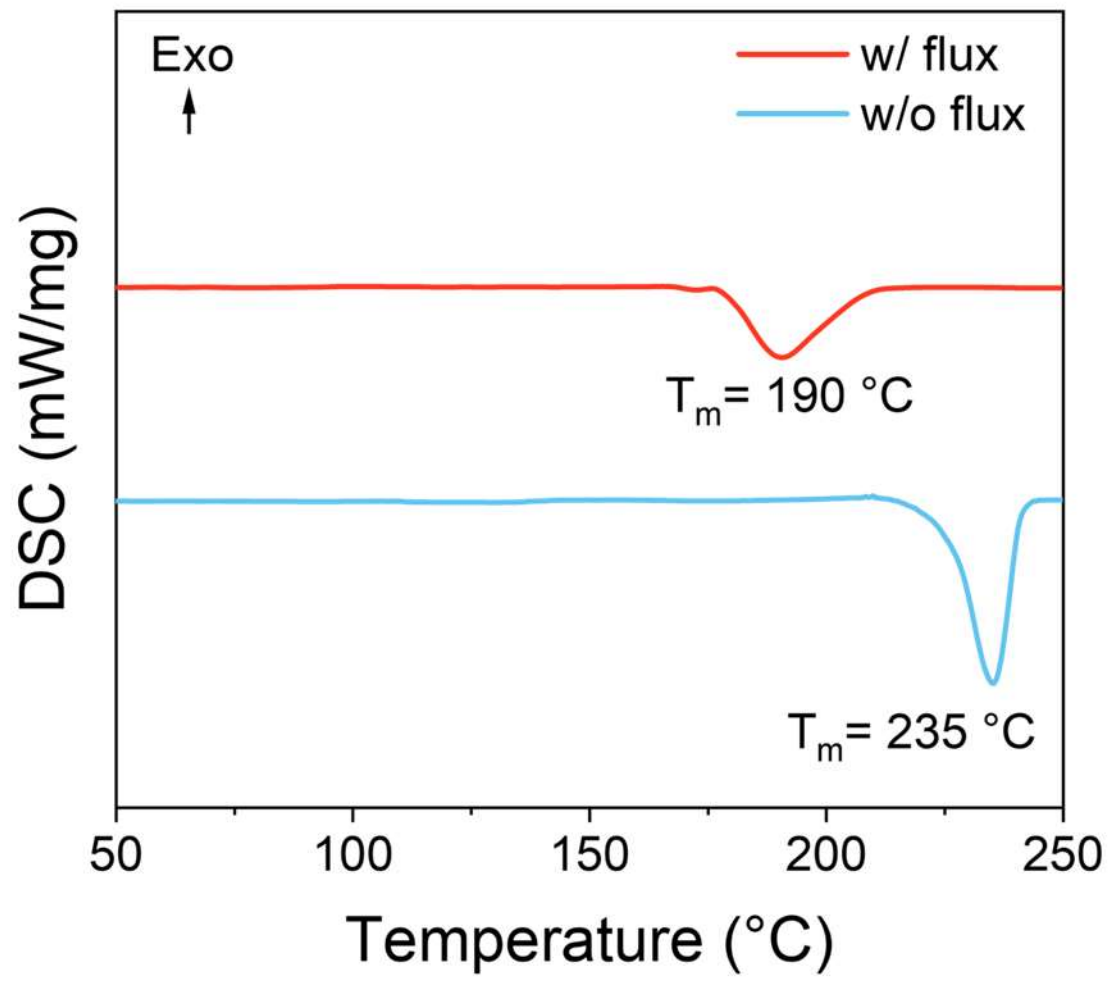

Supplementary Figure 12. DSC curves of DBB and DBB-flux with a standard 10 °C min^{-1} ramping rate. There is about 45 °C reduction in the T_m with the presence of LiOH.

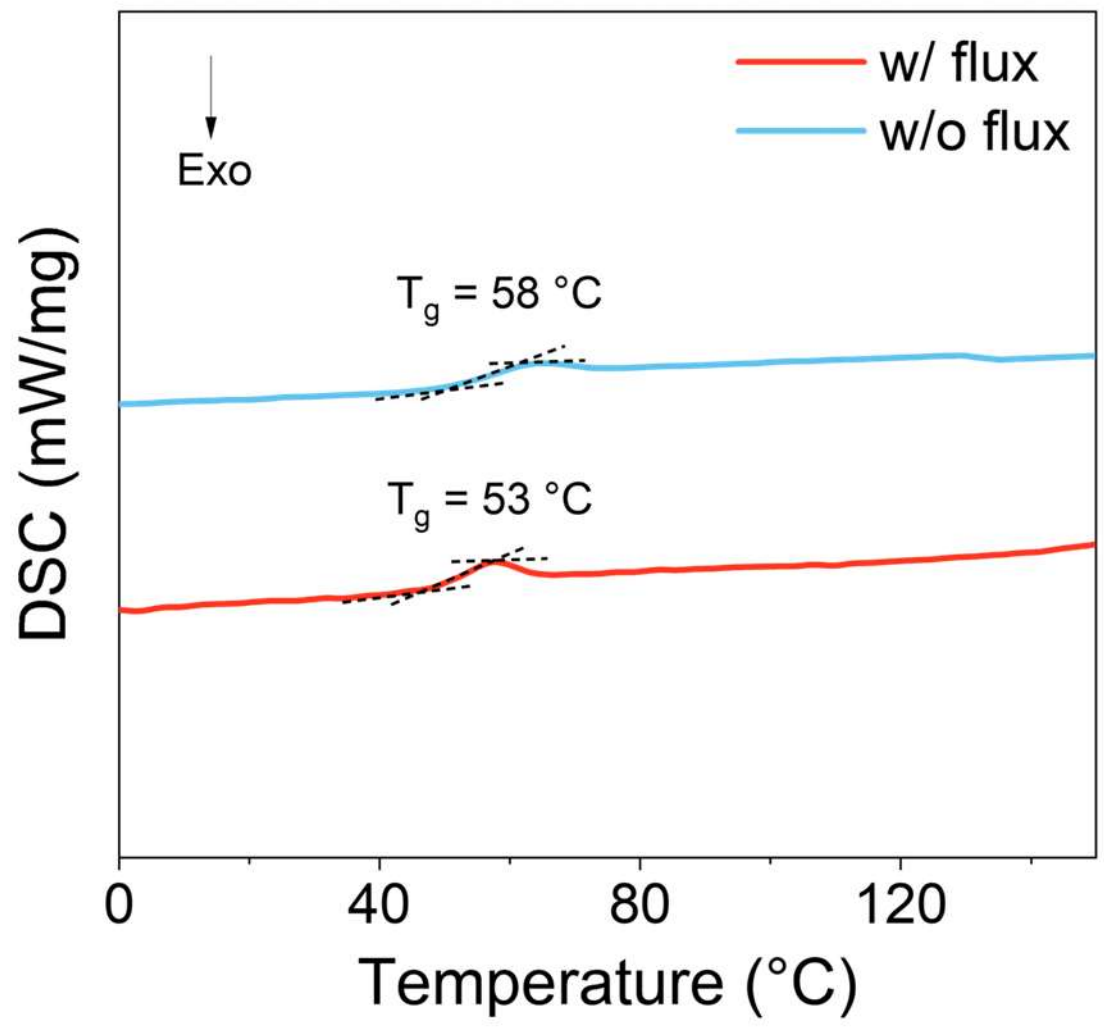

Supplementary Figure 13. DSC curves of a-DBB and a-DBB-flux with a standard 10 °C min^{-1} ramping rate. The introduction of LiOH flux reduces the T_g from 58 °C to 53 °C.

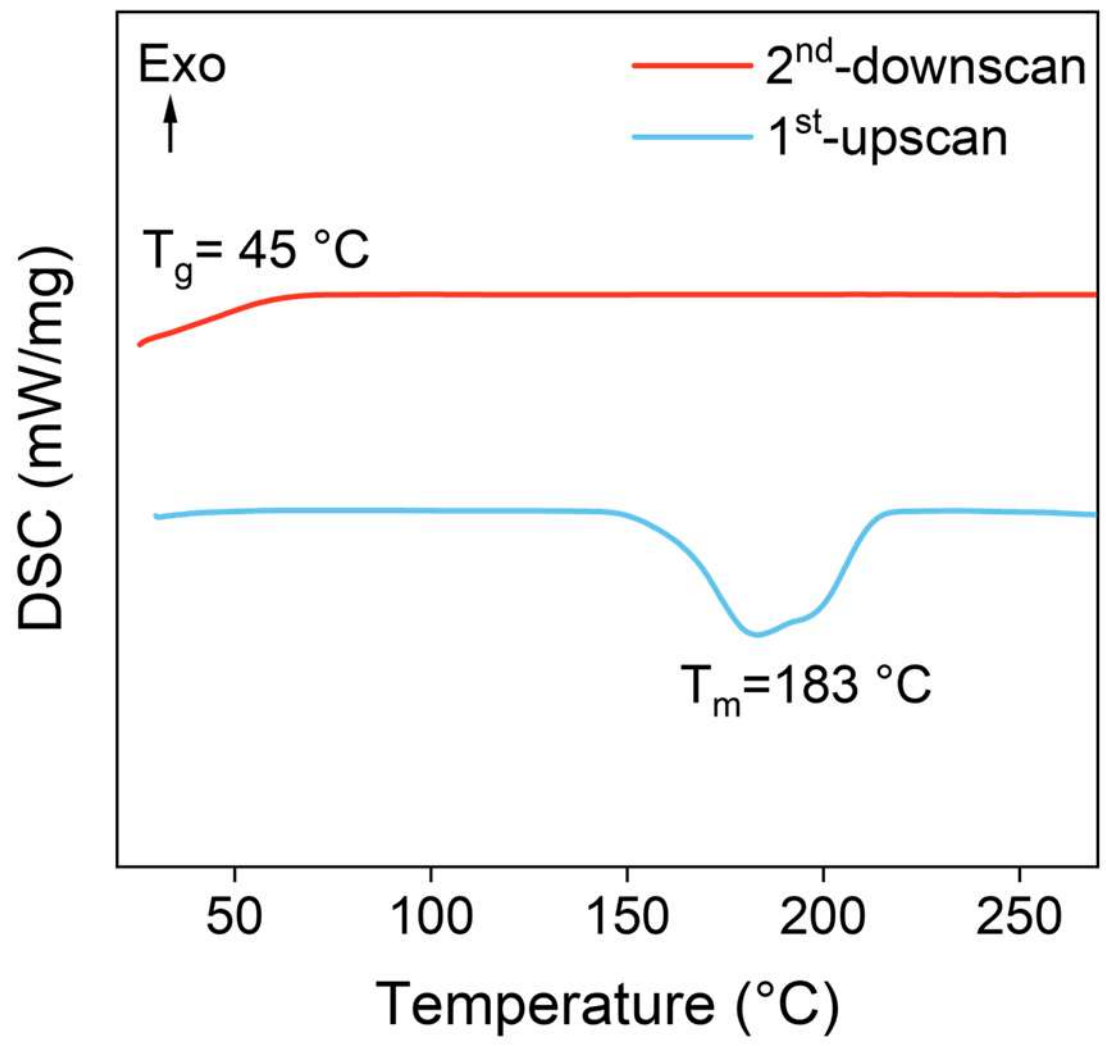

Supplementary Figure 14. DSC curves of EPB-flux. The heating and cooling rates are 10 ℃ min^{-1} and 20 ℃ min^{-1}, respectively. The T_m and T_g values are estimated to be 183 ℃ and 45 ℃ respectively.

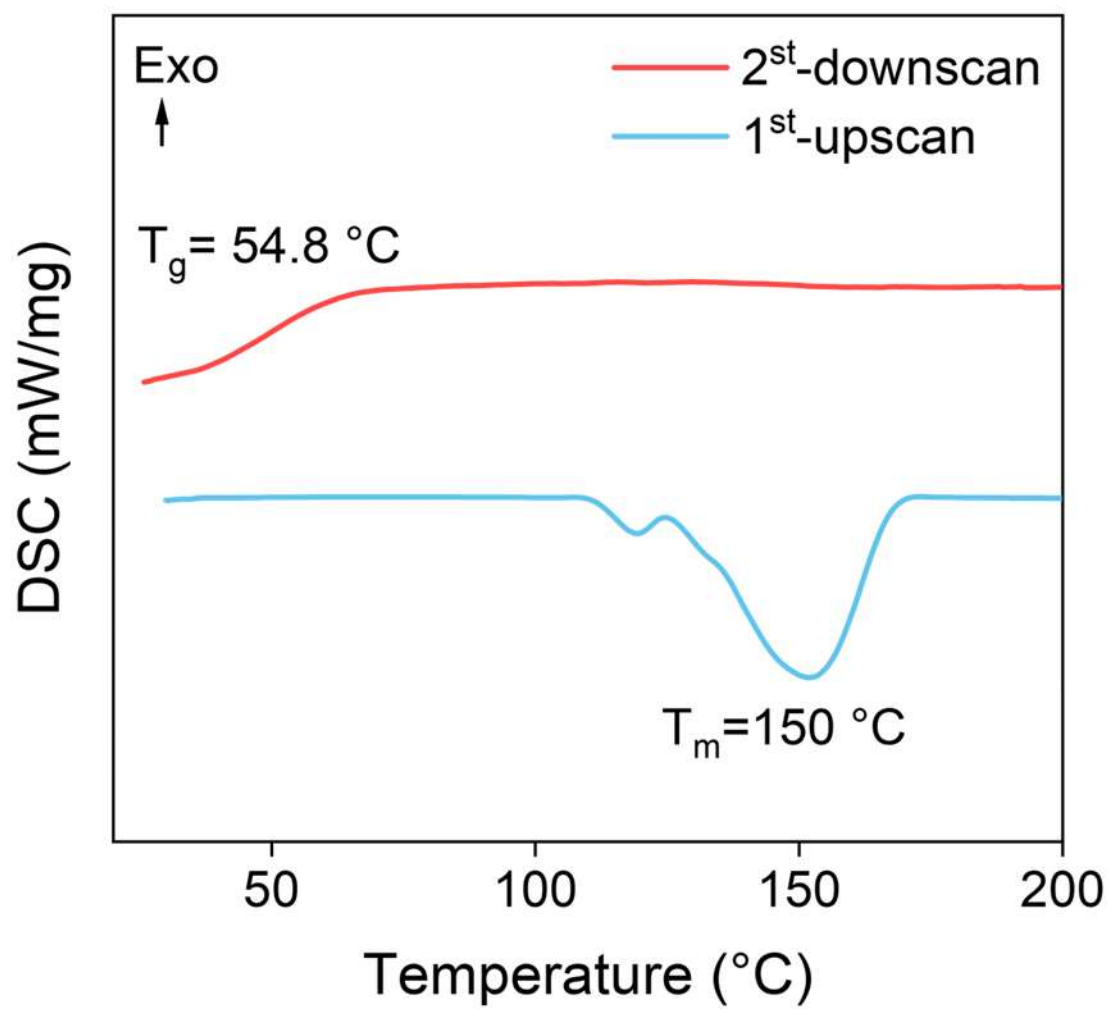

Supplementary Figure 15. DSC curves of RPB-flux. The heating and cooling rates are 10 ℃ min^{-1} and 20 ℃ min^{-1}, respectively. The T_m and T_g are 150 ℃ and 54.8 ℃ respectively.

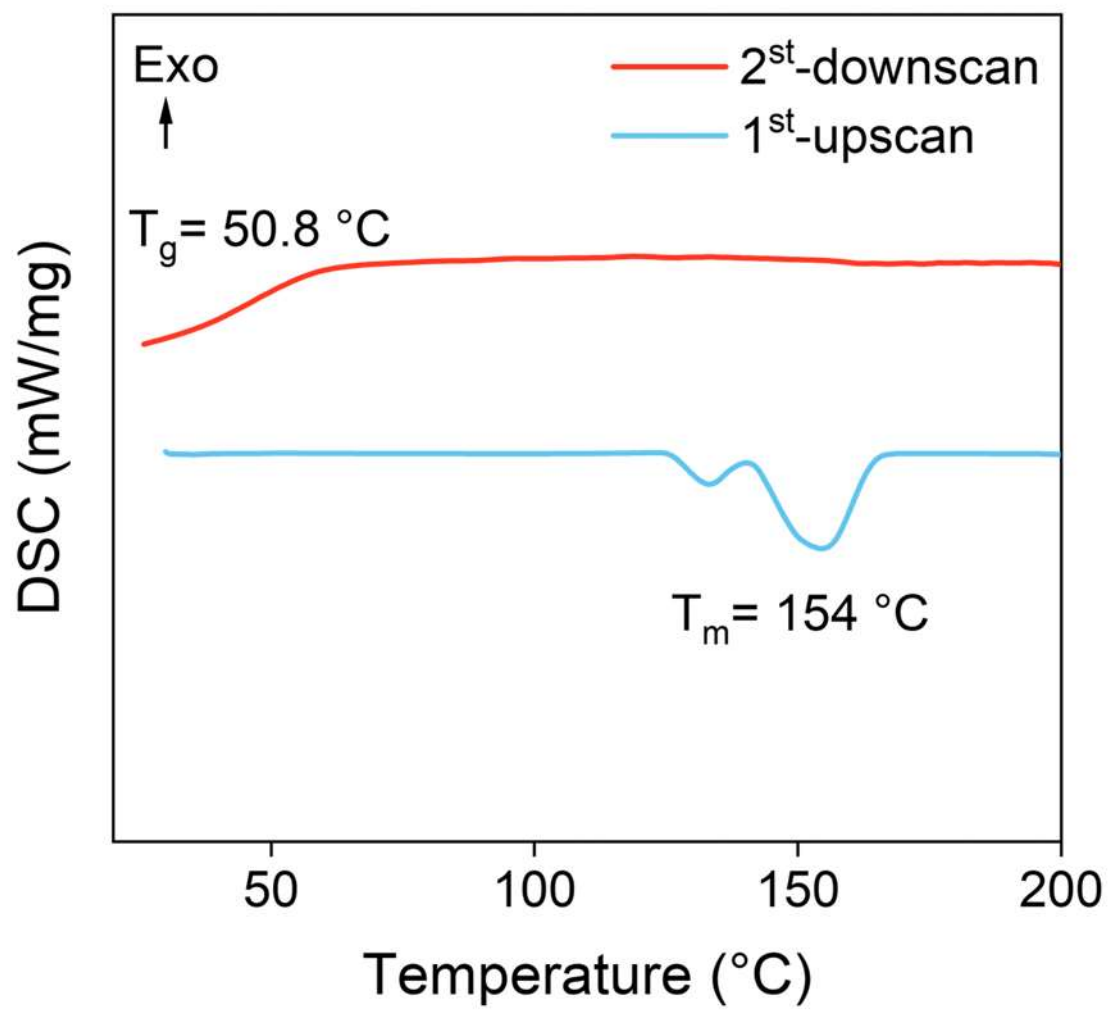

Supplementary Figure 16. DSC curves of SPB-flux. The heating and cooling rates are 10 ℃ min^{-1} and 20 ℃ min^{-1}, respectively. The T_m and T_g are 154 ℃ and 50.8 ℃ respectively.

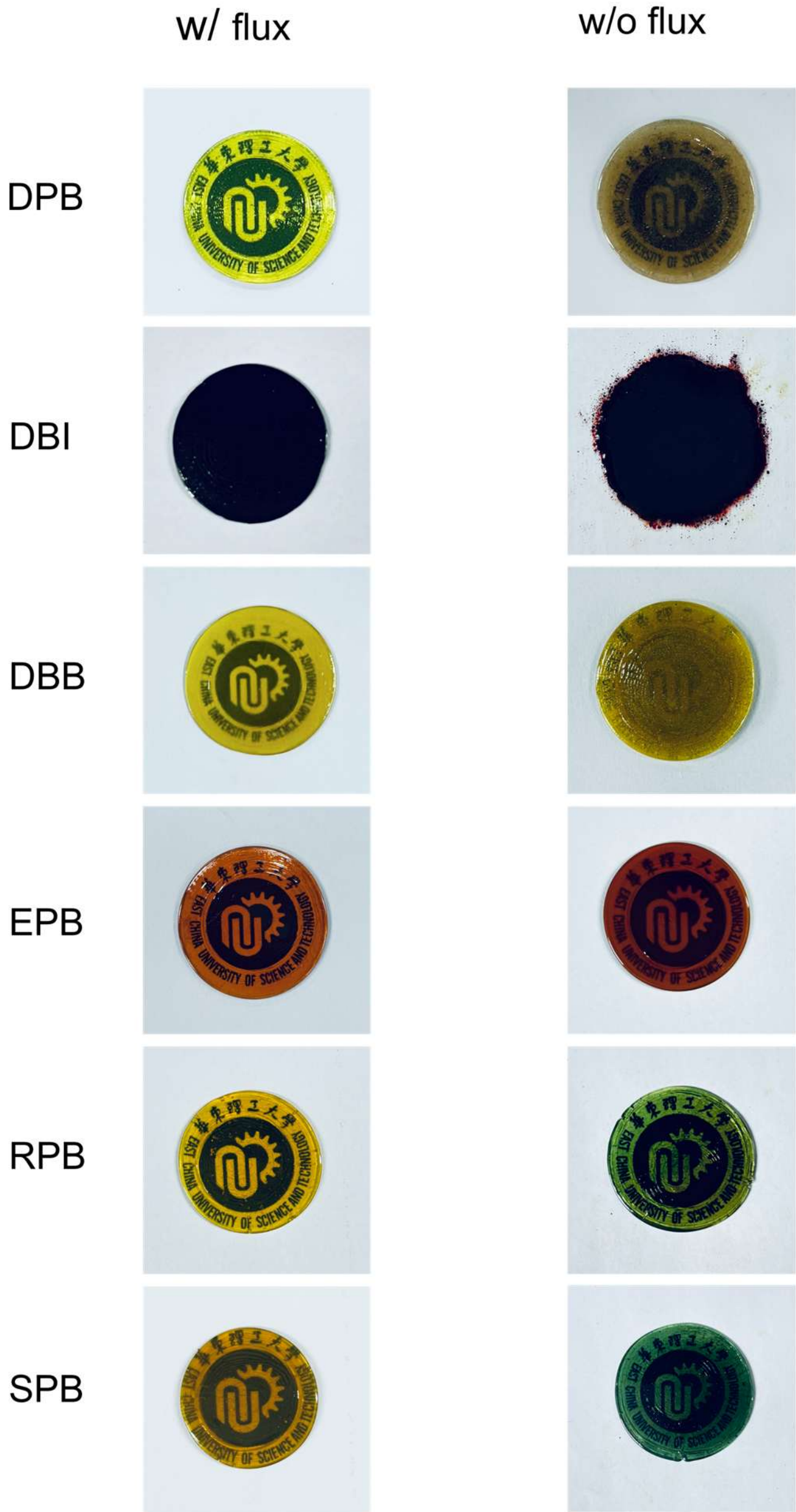

Supplementary Figure 17. Photographs of different doped and undoped glasses. Most doped ones exhibited good transmittance without notable degradation byproducts (thickness: 2 mm).

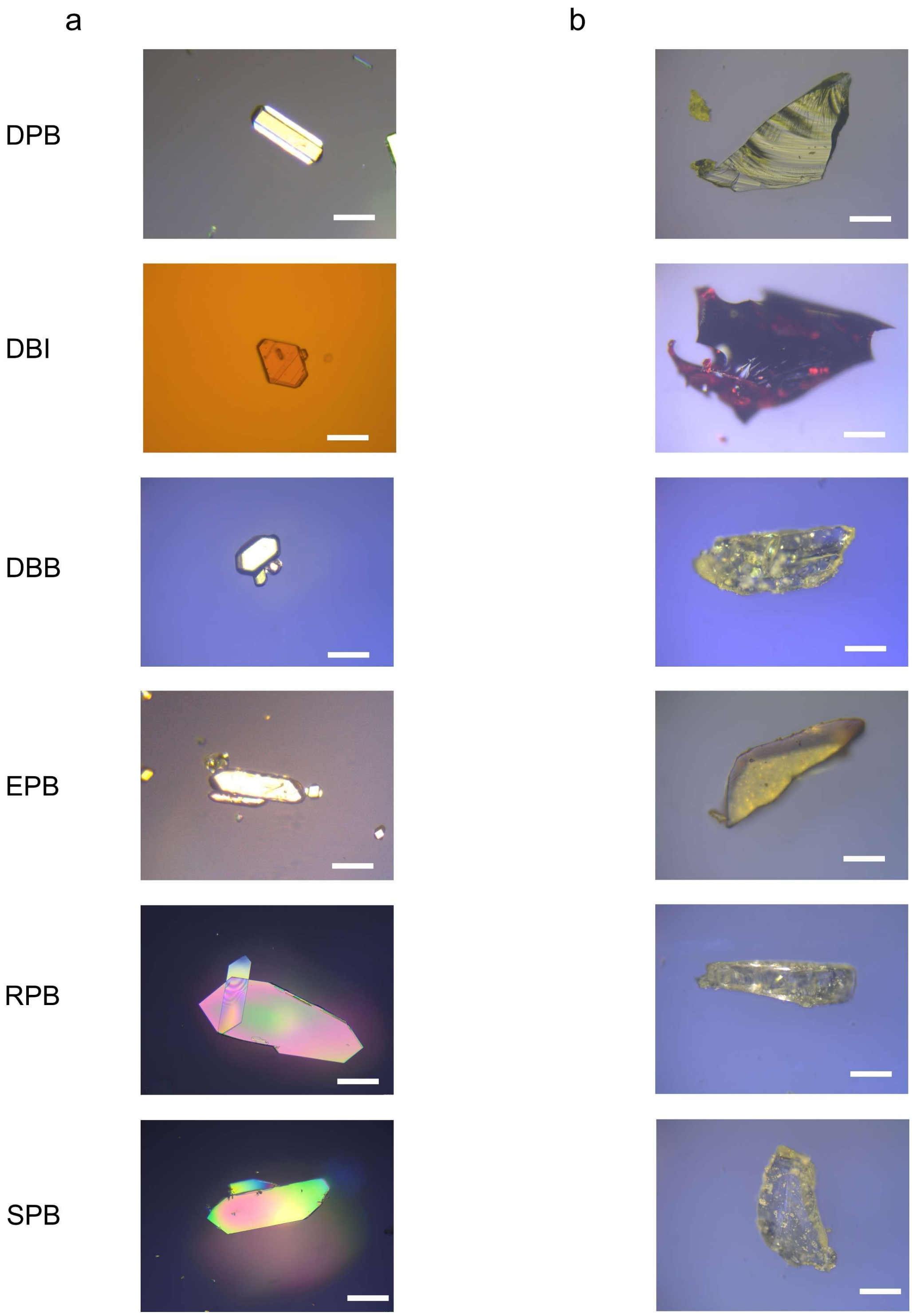

Supplementary Figure 18. Optical microscopy images of different (a) crystals and (b) glasses. All glasses were synthesized with LiOH flux. Under polarized light, the crystals exhibit distinct reflectance behavior from single-crystalline surfaces, whereas the glasses are transparent (scale bars: 100 µm).

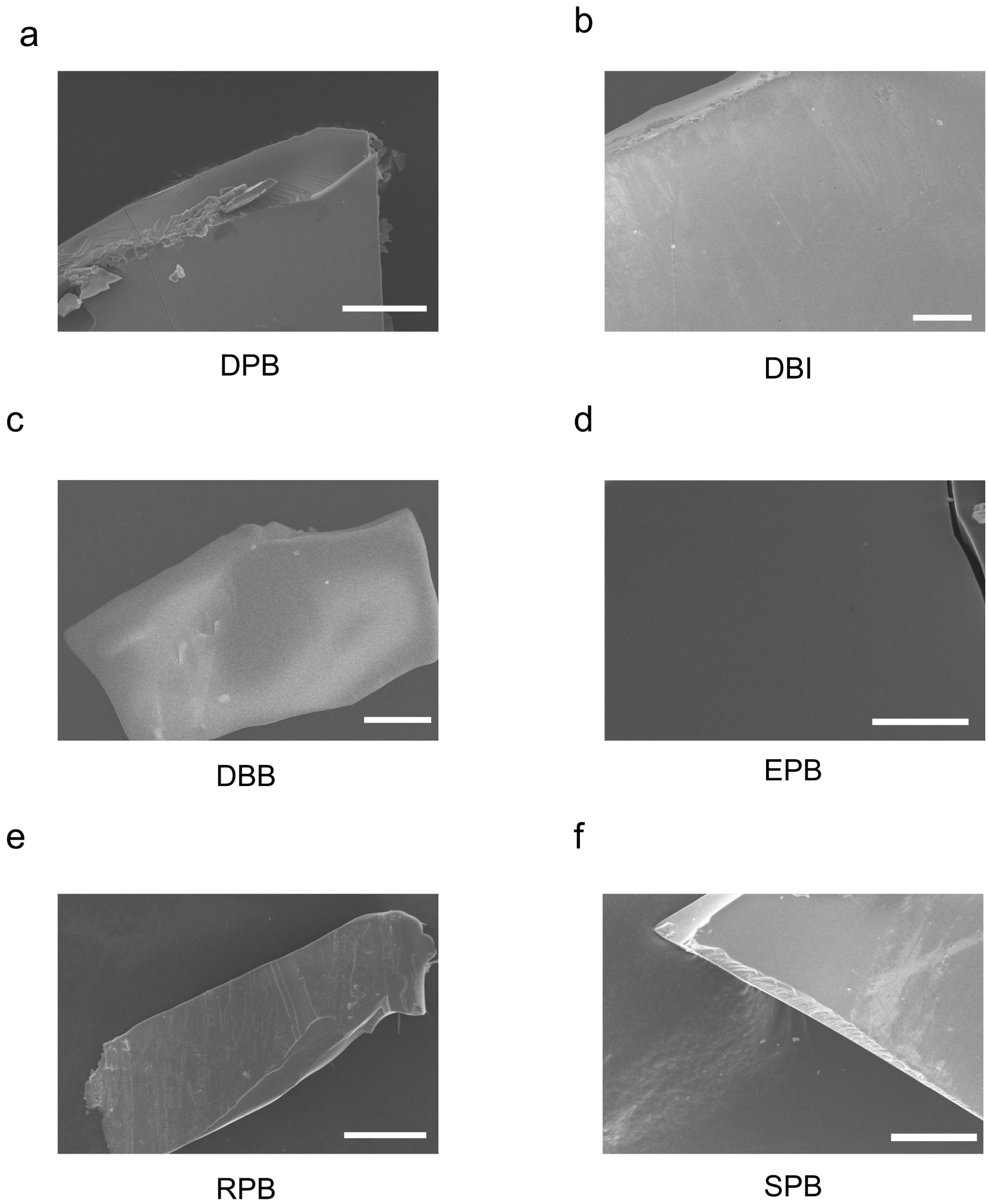

Supplementary Figure 19. SEM images of different glasses with LiOH. All glasses exhibit irregular shape and fracture surface. Scale bars are 50 μm in (a, b, e, f), 10 μm in (c), and 25 μm in (d), respectively.

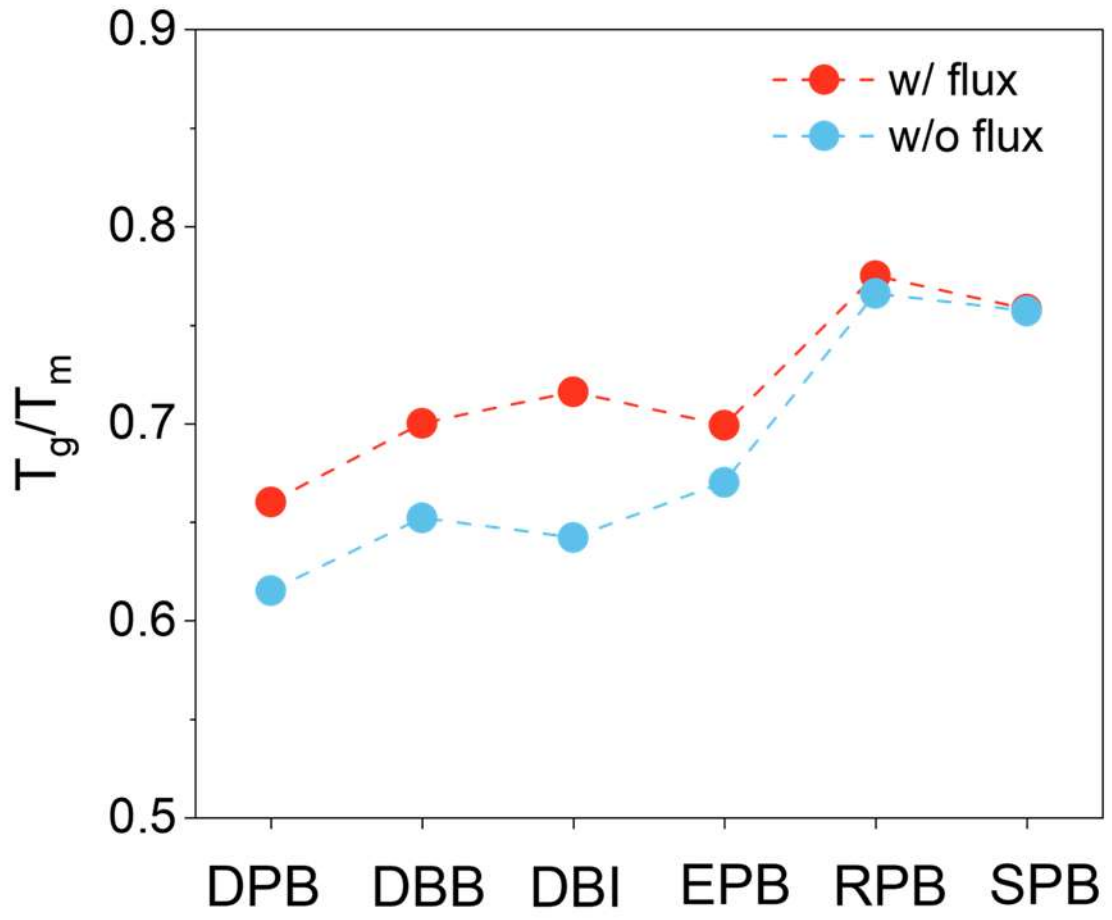

Supplementary Figure 20. T_g/T_m diagram of glasses with and without LiOH fluxes. The addition of fluxes enhances the glass-forming ability, with $T_g/T_m > 2/3$.

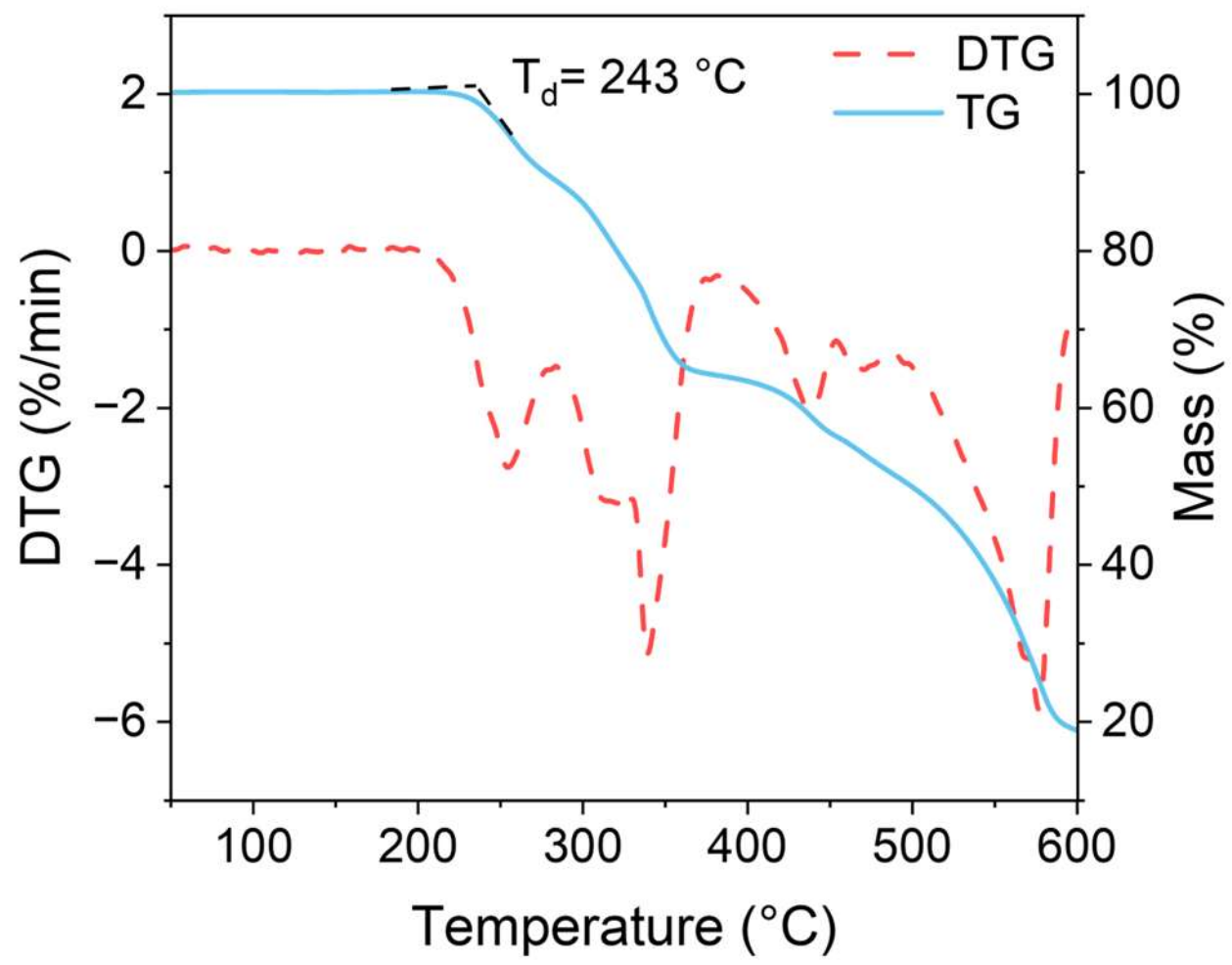

Supplementary Figure 21. TGA curves of DPI with a standard 10 °C min^{-1} ramping rate. The T_d value is determined to be about 243 °C.

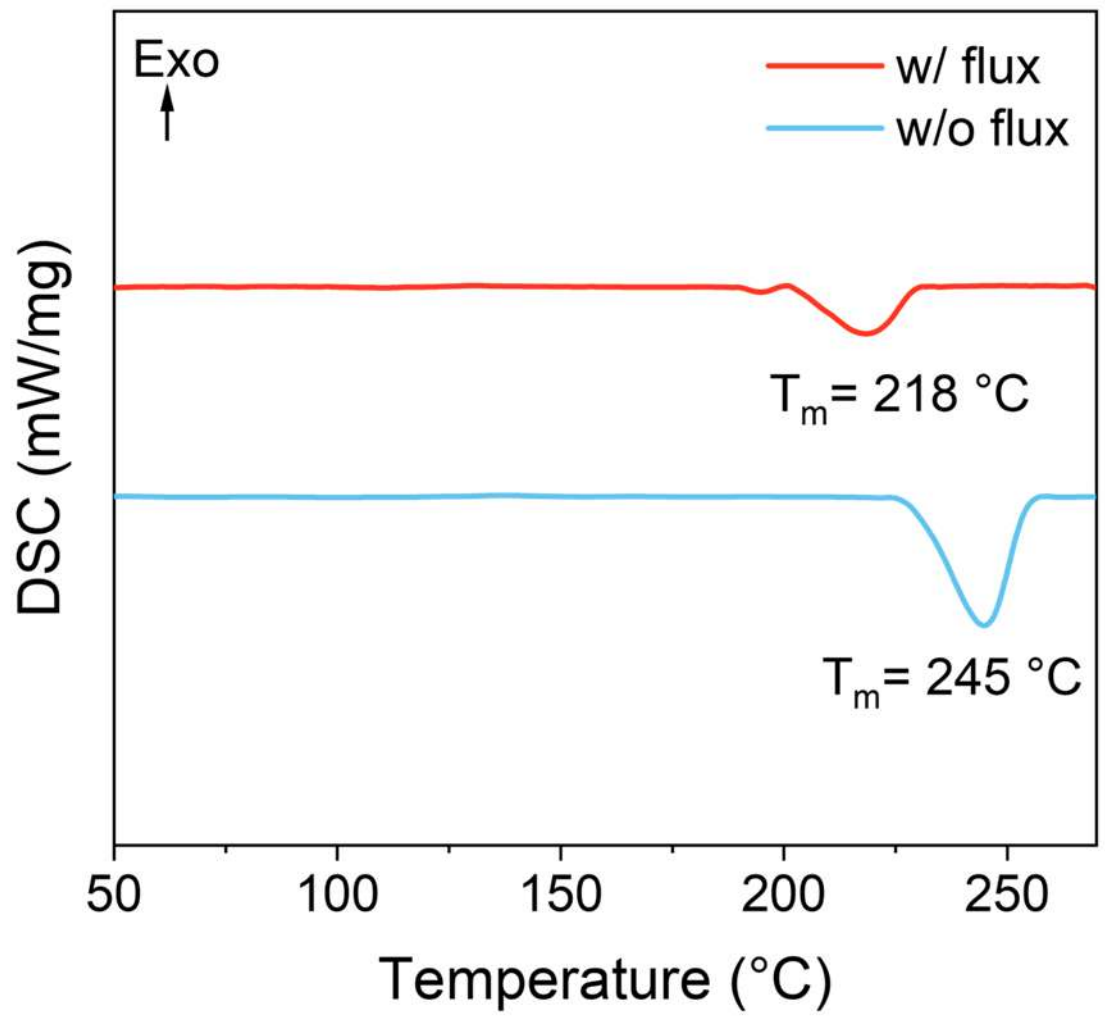

Supplementary Figure 22. DSC curves of DPI and DPI-flux with a standard 10 °C min^{-1} ramping rate. The presence of LiOH reduces the T_m from 245 °C to 218 °C.

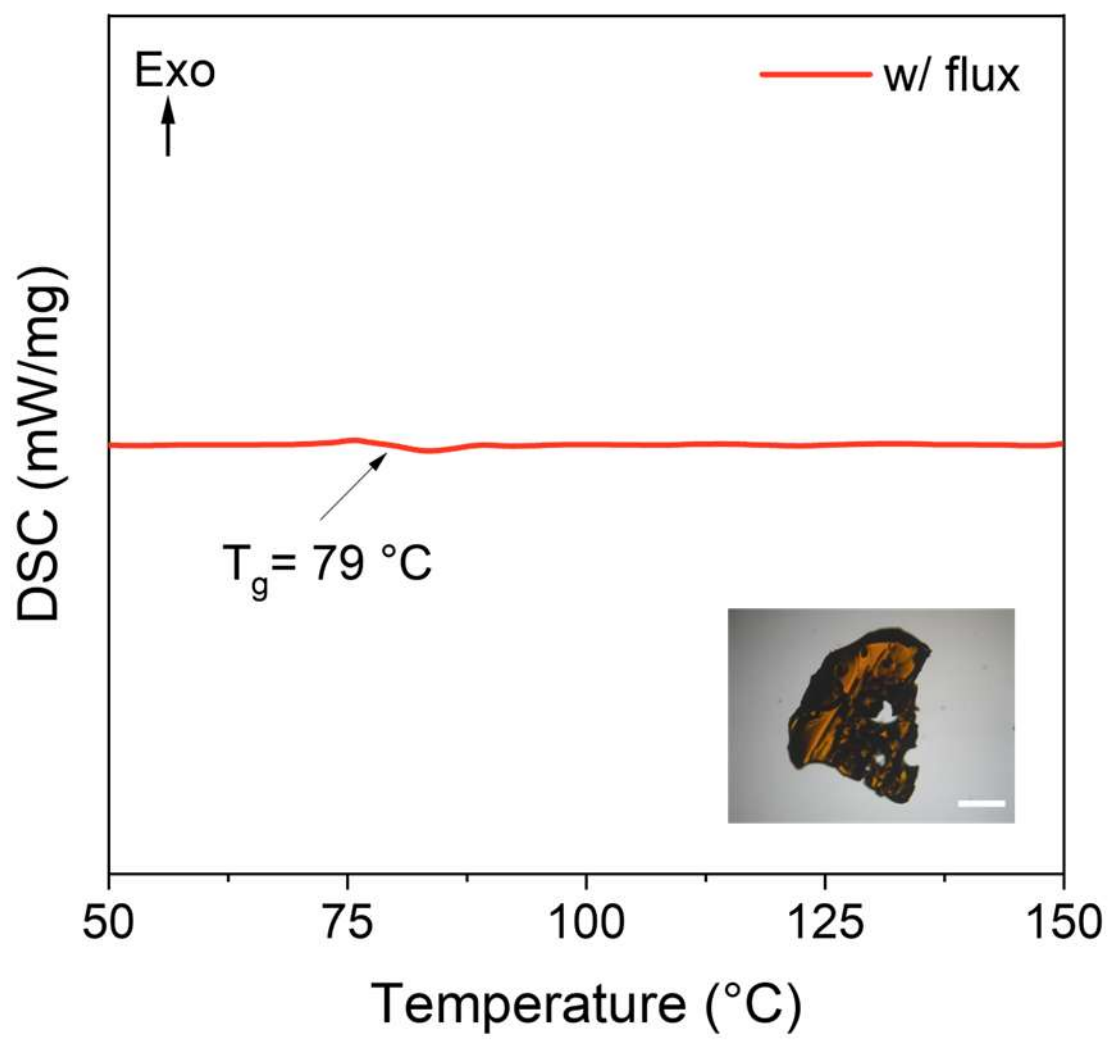

Supplementary Figure 23. DSC curves of a-BPI-flux with a standard 10 °C min^{-1} ramping rate. The T_g value is determined to be about 79 °C. Inset is an optical microscopy image of the glass (scale bar: 100 μm).

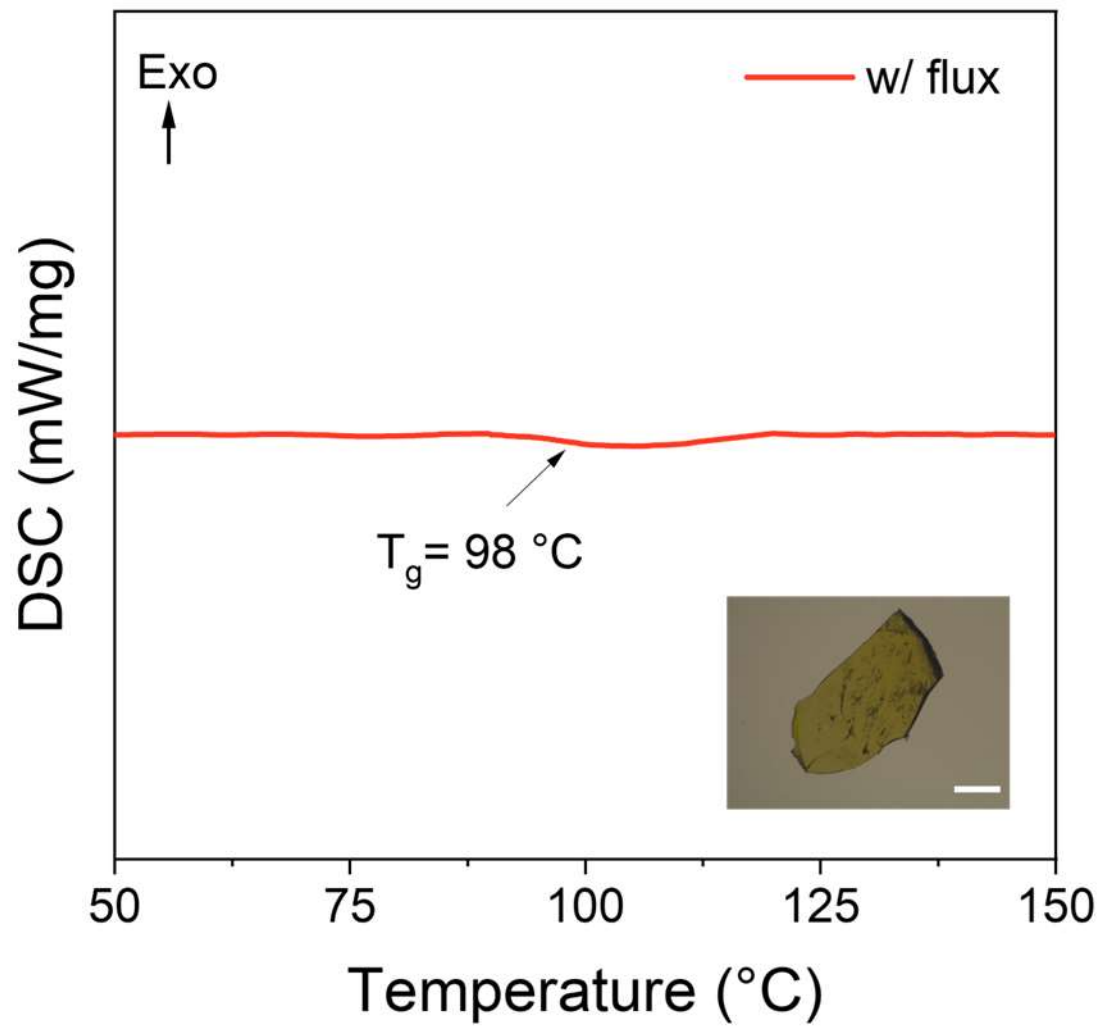

Supplementary Figure 24. DSC curves of a-DPI-flux with a standard 10 °C min^{-1} ramping rate. The T_g value is determined to be about 98 °C. Inset is an optical microscopy image of the glass (scale bar: 100 μm).

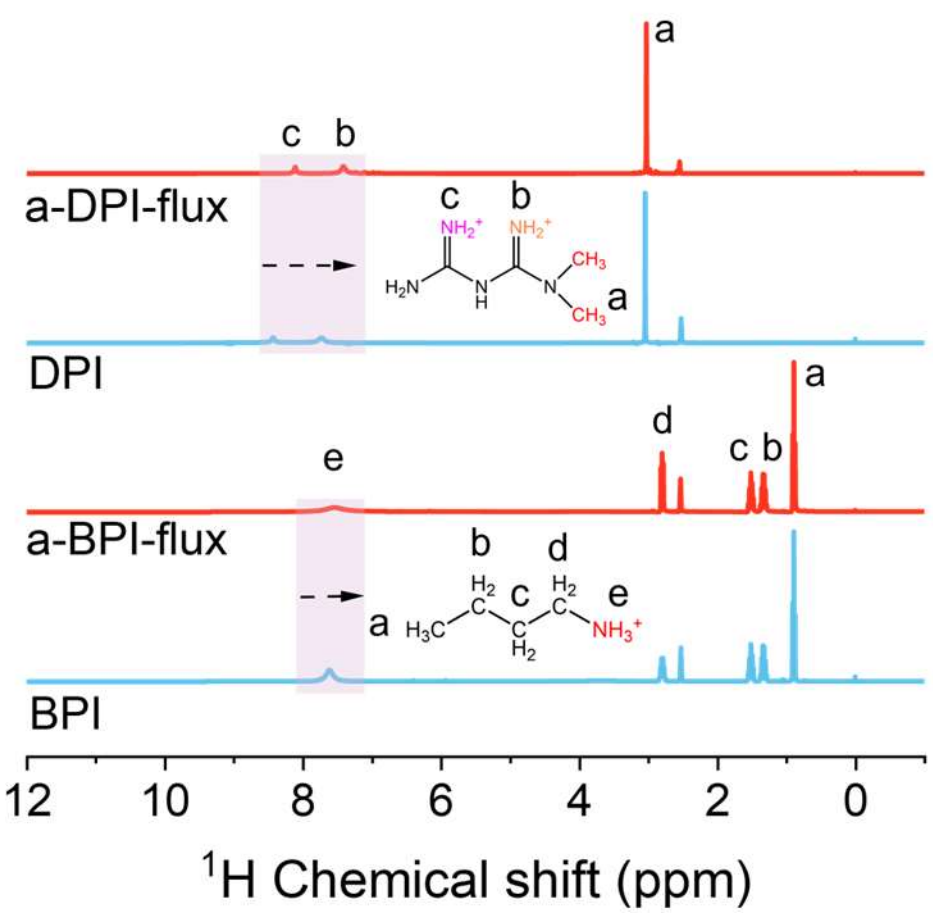

Supplementary Figure 25. ^1H NMR spectra of crystalline and glassy BPI and DPI.

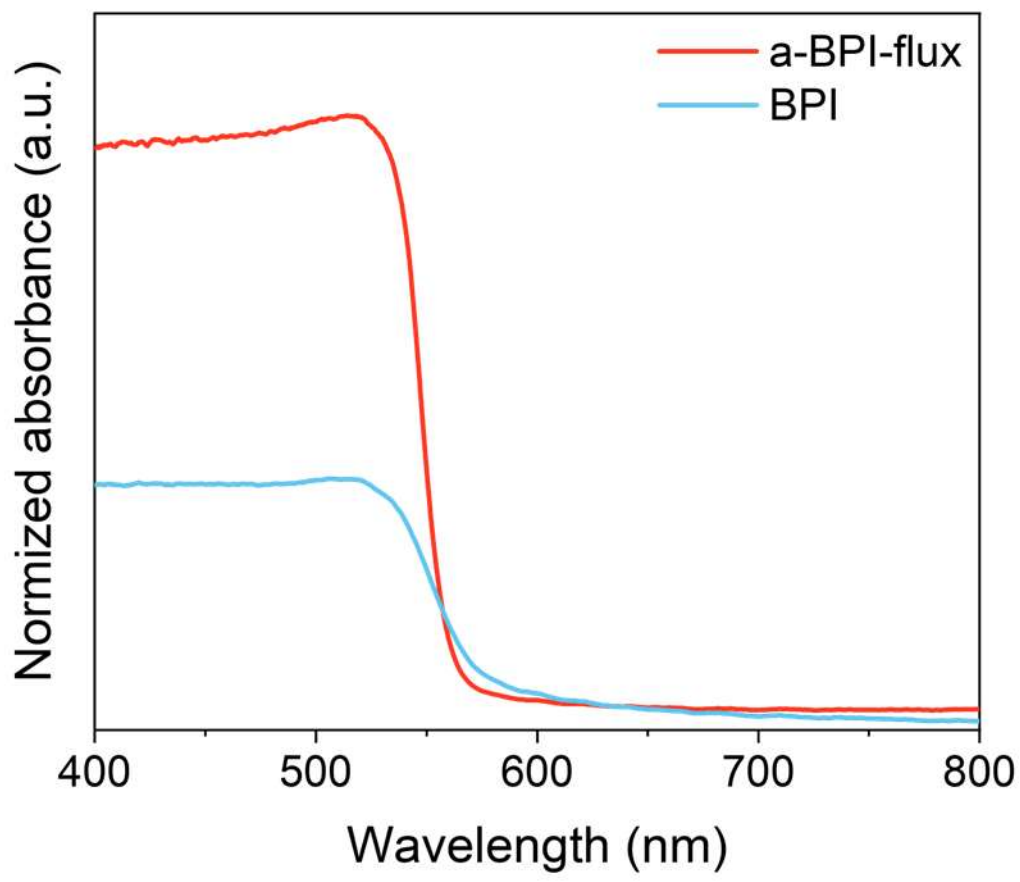

Supplementary Figure 26. UV-vis absorbance profile of the BPI and a-BPI-flux.

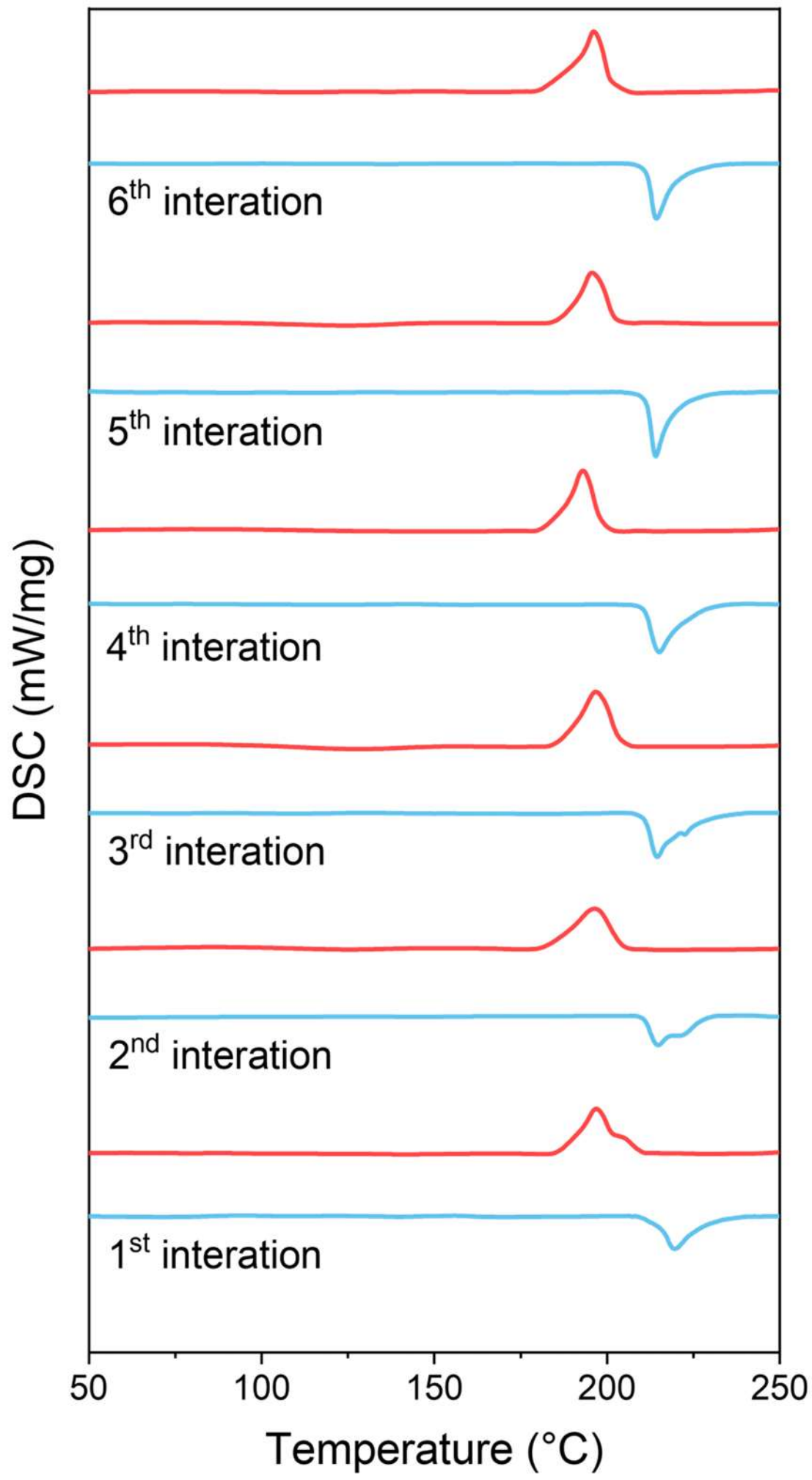

Supplementary Figure 27. DSC curves of doped BPI during six heating-cooling cycles. The heating and cooling rate are 10 °C min^{-1}. The initial shift in crystallization onset originates from several possible mechanisms, including the structural relaxation of the first melt, redistribution/consumption of residual hydroxide, release of trace water, and homogenization of Li^+ coordination during the early cycles. These changes alter the effective nucleation barrier and shift the crystallization onset. After the initial cycles, the crystallization/melting peaks become reproducible, indicating that the BPI-flux melt reaches a stable and reversible crystal/melt transformation regime. The consistency of each cycle in the melting and crystallization peaks suggests excellent thermal stability of the BPI melt.

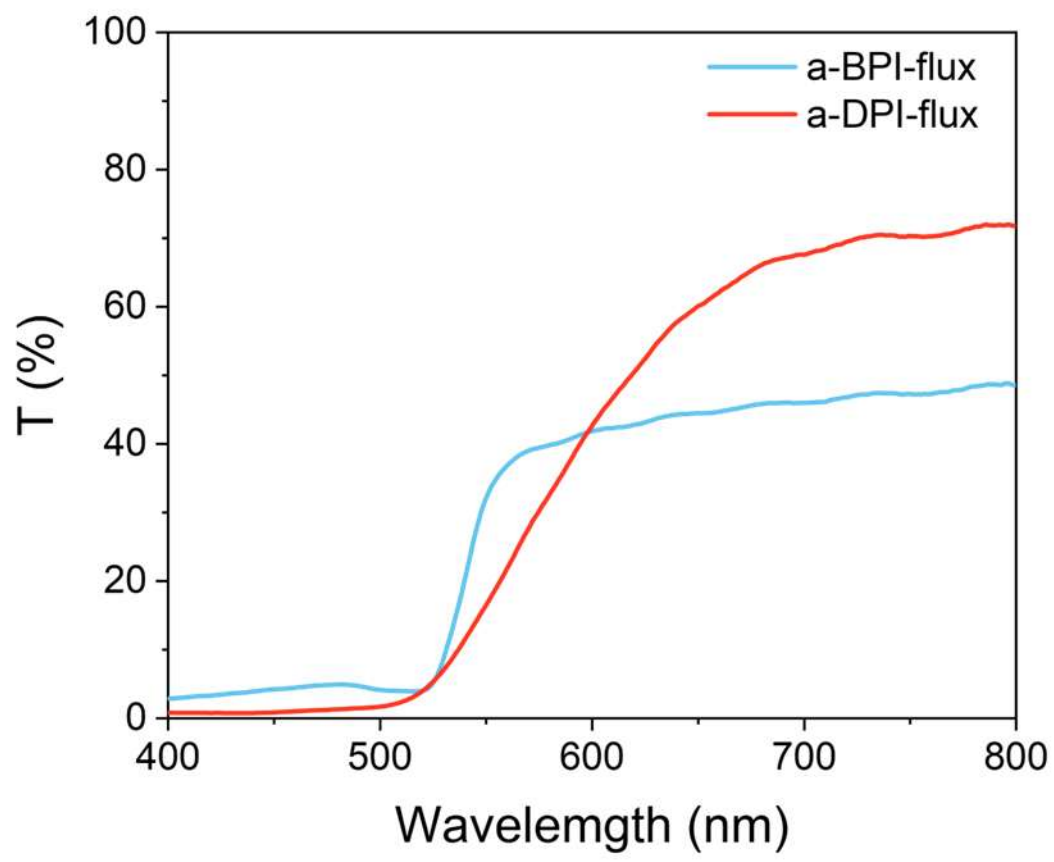

Supplementary Figure 28. UV-vis transmittance spectra of the a-BPI-flux and a-DPI-flux sheets with a thickness of 0.5 mm.

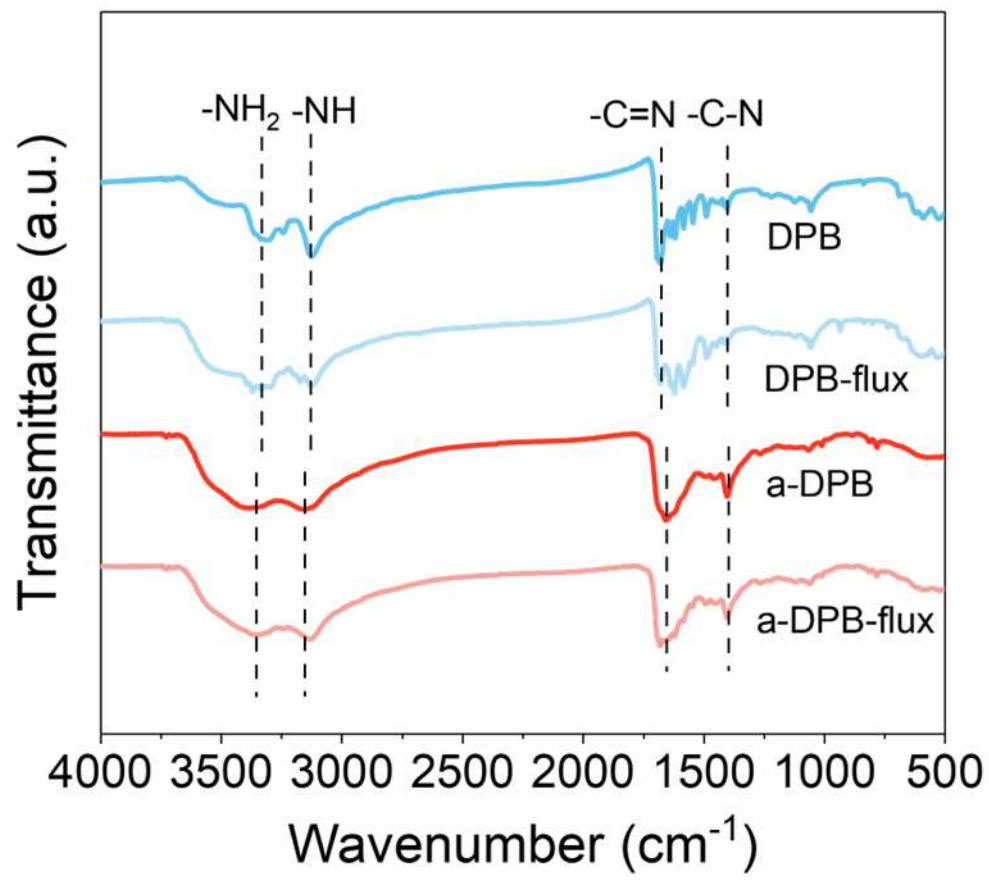

Supplementary Figure 29. Fourier transform infrared (FTIR) spectra of doped and undoped DPB crystals and glass. In the glassy phase with LiOH, the shift of the C=N peak position indicates that the deprotonation process causes changes in the electron cloud around N, while the C–N peak is less affected by proton loss and remains almost unchanged. In the crystalline phase, LiOH induced deprotonation only leads to slight decrease in the intensity of C=N and NH vibrations, without significant stretching shifts, due to the diffusion-limited reaction kinetics during the grinding process.

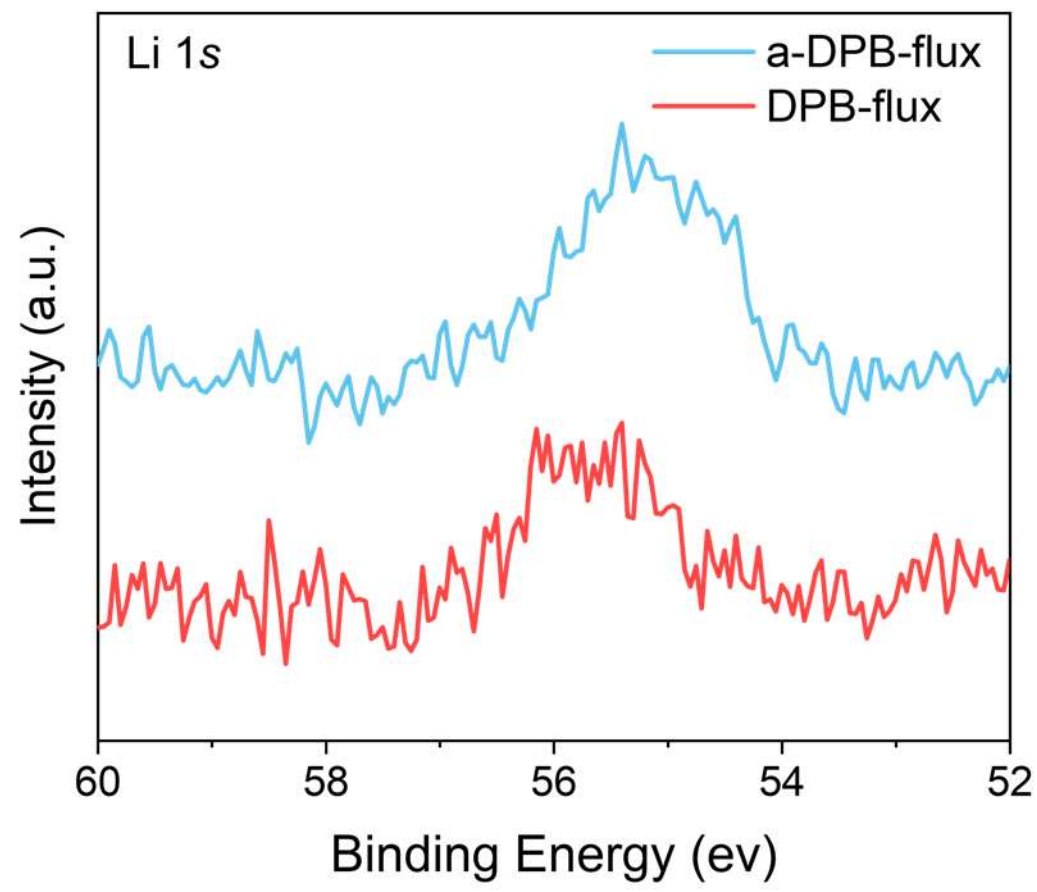

Supplementary Figure 30. XPS spectra of DPB-flux and a-DPB-flux. The shift of Li 1*s* peak implies the coordination environment evolution upon the reaction between LiOH and perovskites.

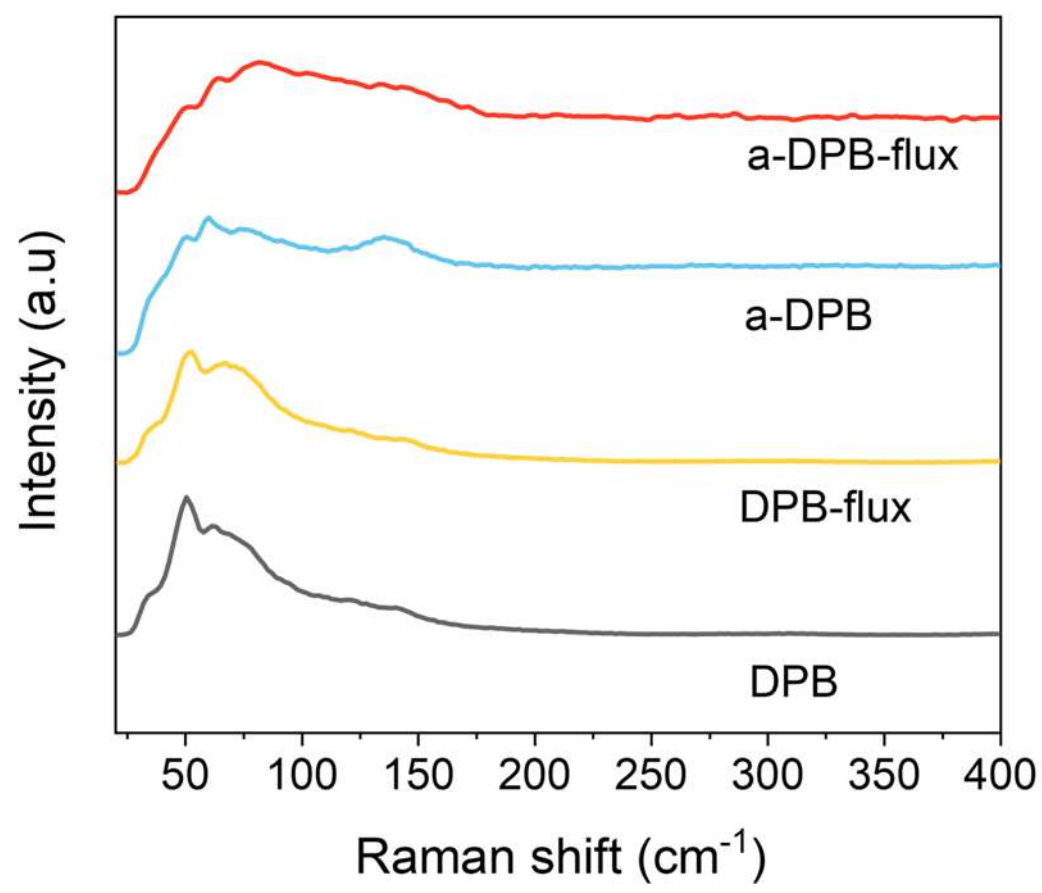

Supplementary Figure 31. Raman spectra of DPB crystals and glasses. Progressive peak broadening from crystalline to glassy states reveals structural ordering-to-disorder transition.

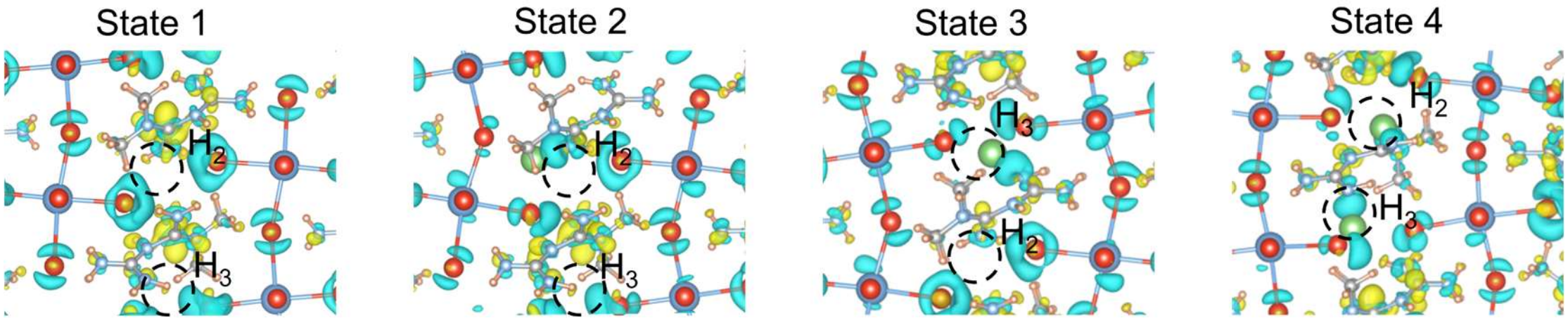

Supplementary Figure 32. Differential charge density in four states. The positions of H_2 and H_3 are marked by dotted lines. The results show that as H_2 and H_3 are removed, the electrostatic interaction between the inorganic skeleton and the organic molecule is weakened.

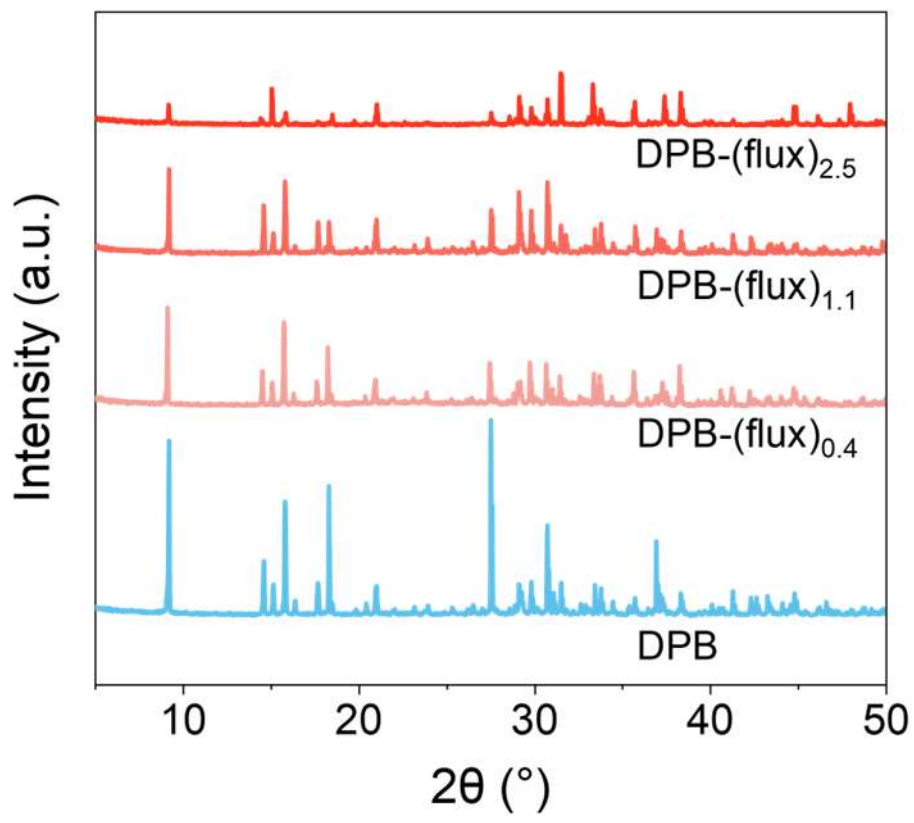

Supplementary Figure 33. XRD patterns of DPB samples with varying LiOH doping ratios. At low- LiOH loadings, the major DPB diffraction peaks are largely retained, with only minor intensity variations, indicating that room-temperature grinding induces only limited interfacial deprotonation and does not collapse the crystalline framework. At high LiOH loadings, the diffraction intensity decreases more evidently, a characteristic of markedly reduced crystallinity.

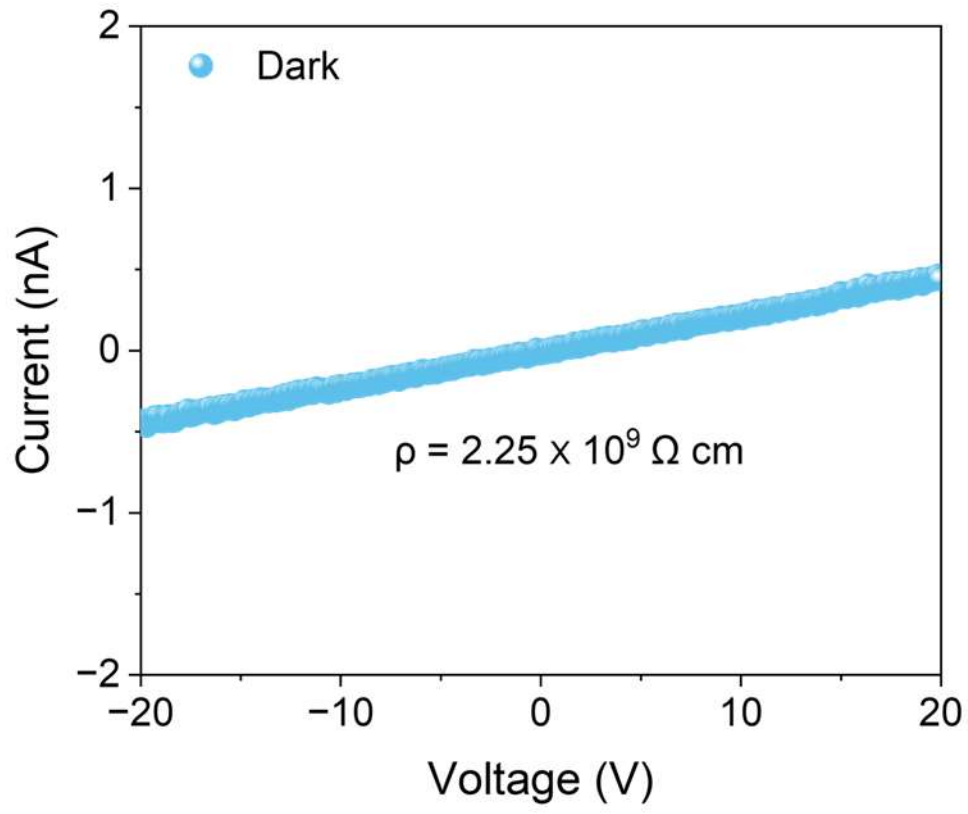

Supplementary Figure 34. Dark I-V curve of a-DPB-flux device based on a vertical Ag/glass/Ag configuration. The electronic resistivity of a-DPB-flux is calculated to be 2.25×10^9 Ω cm.

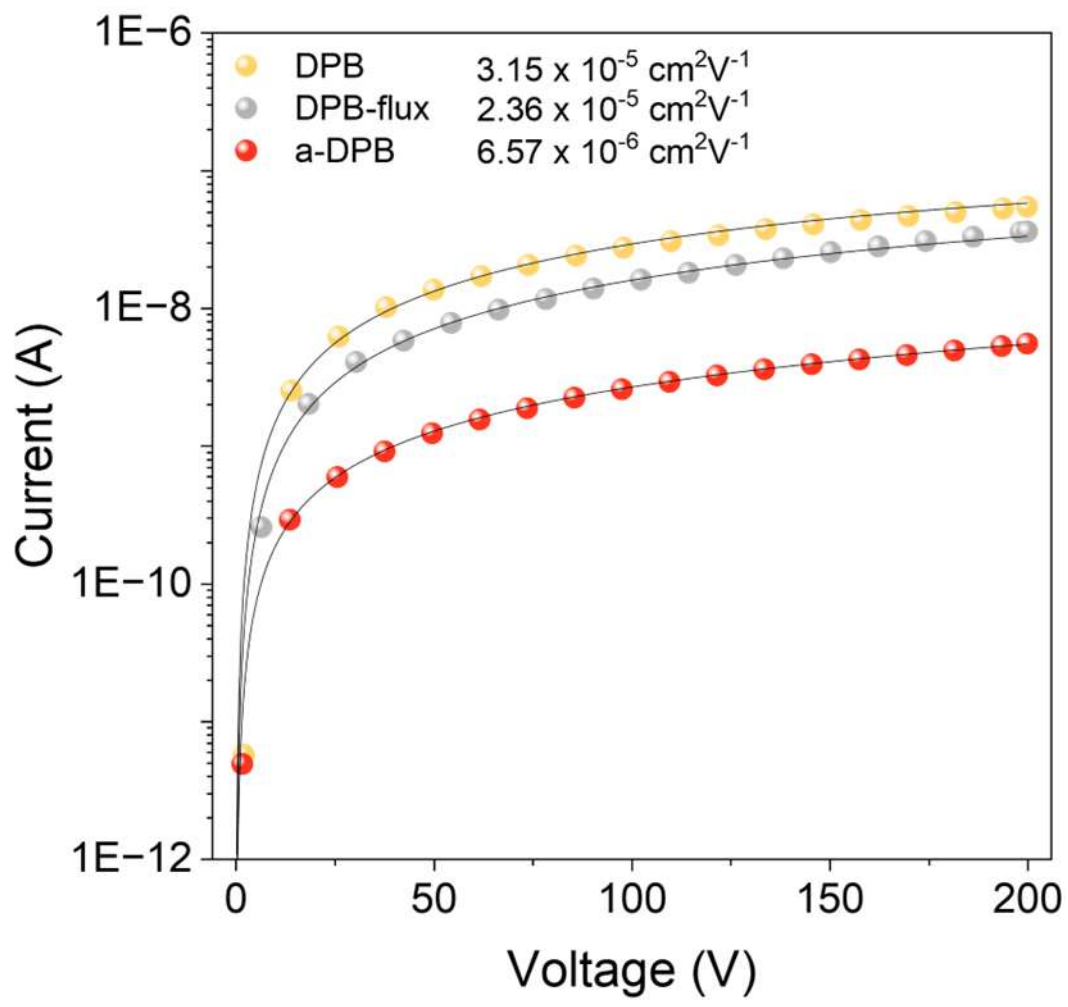

Supplementary Figure 35. Photoconductivity characterization of DPB, DPB-flux and a-DPB device.

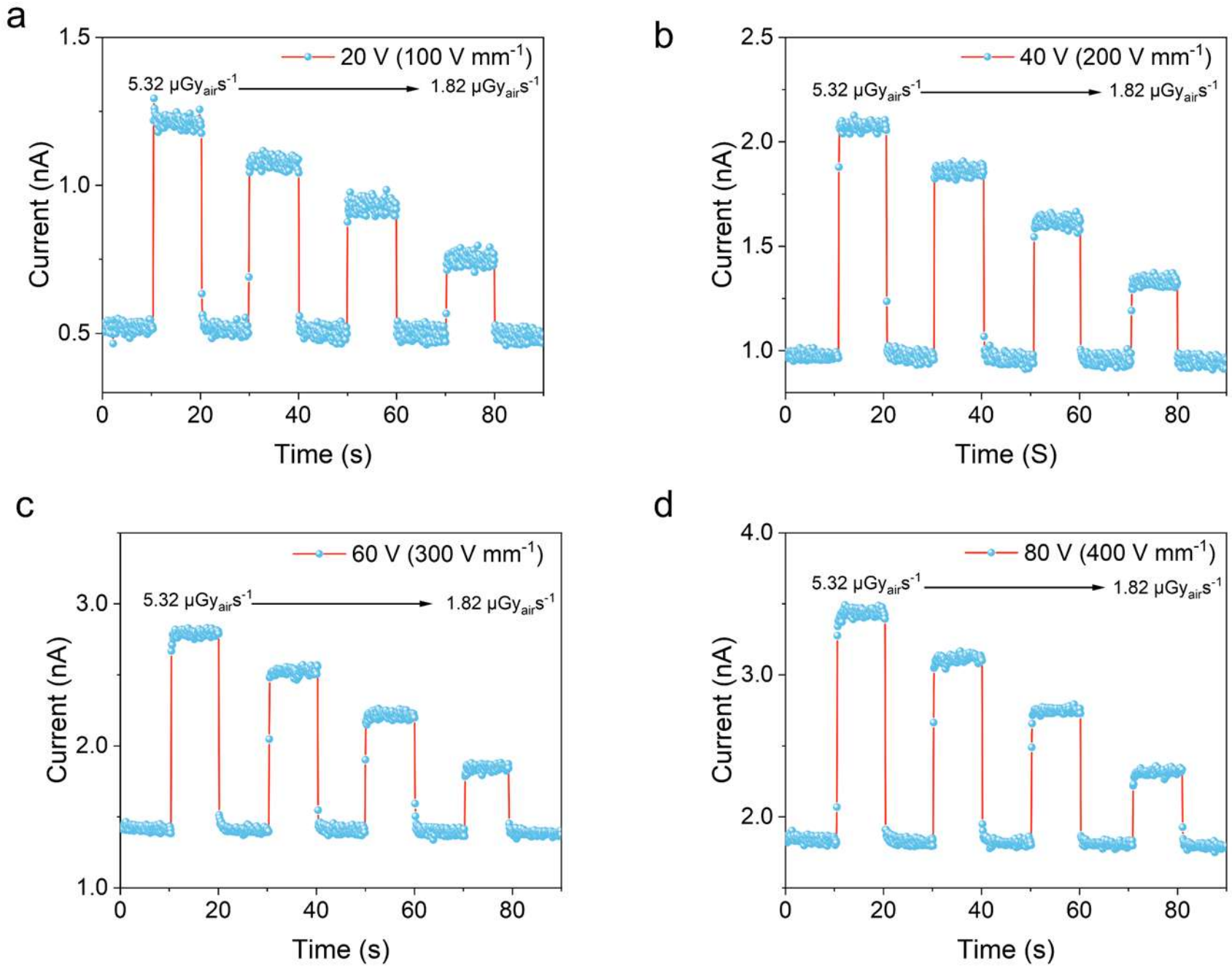

Supplementary Figure 36. (a-d) On/off photocurrent responses of the a-DPB-flux device under different X-ray dose rates and at different bias voltage.

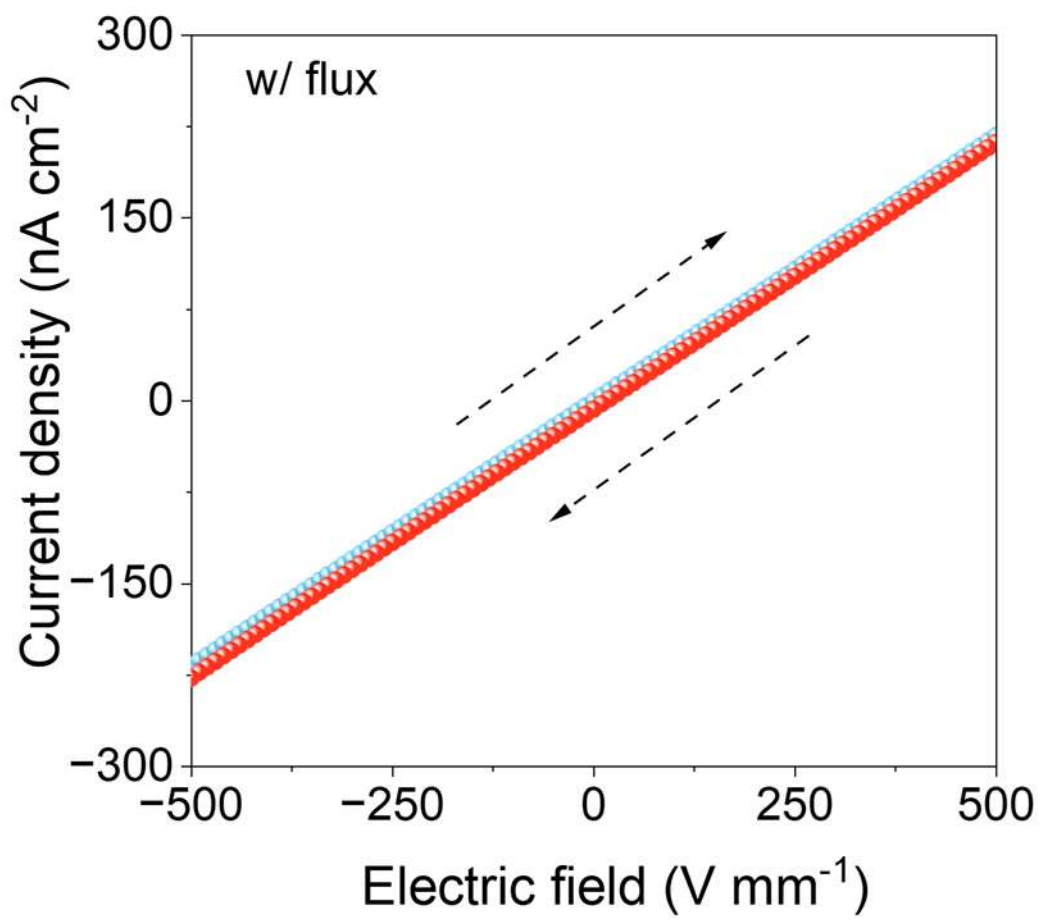

Supplementary Figure 37. Cyclic dark I-V curves (10 V s^{-1}) of the Ag/a-DPB-flux/Ag device.

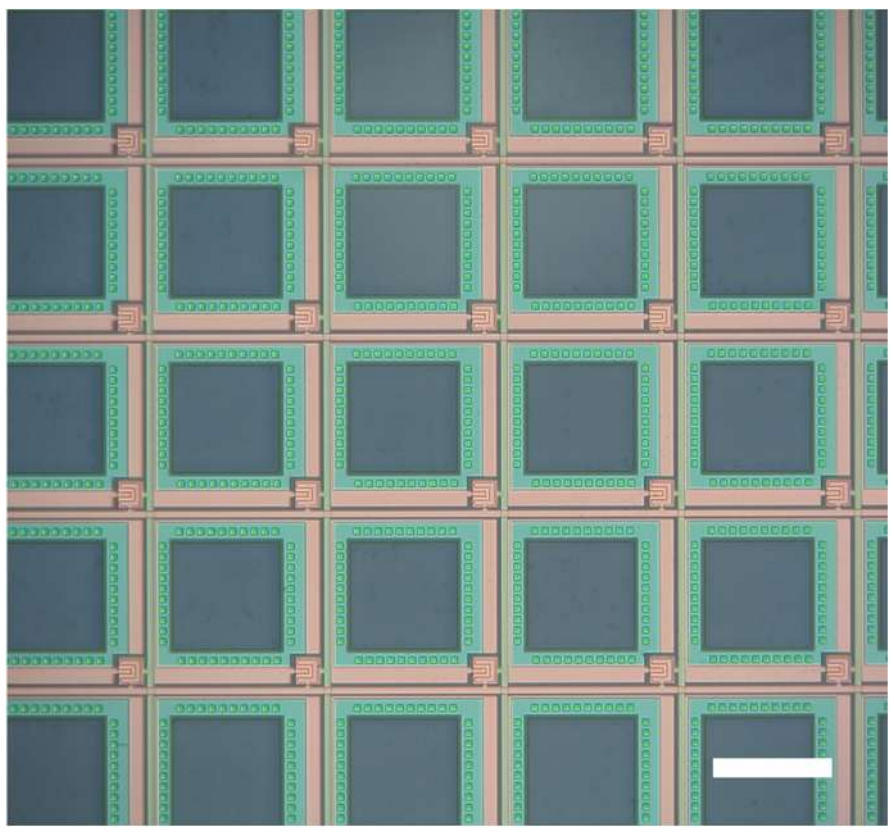

Supplementary Figure 38. Optical microscope image of a TFT backplane (scale bar: 250 μm).

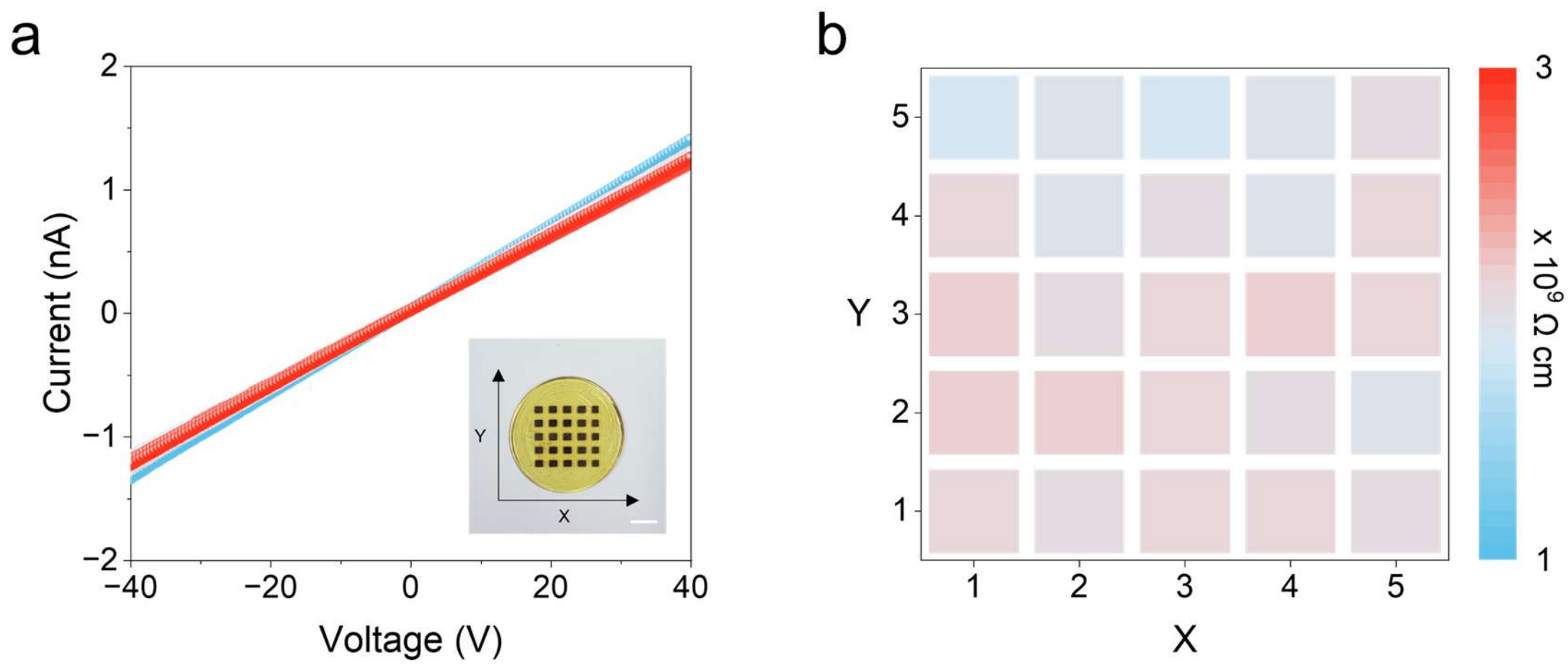

Supplementary Figure 39. (a) Dark I-V curve of a large-area a-DPB-flux device with a 5 × 5 array of metal electrodes. The inset shows a photograph of the device (scale bar: 1cm). (b) Statistical plot of resistivity for 25 individual pixels of the Ag/a-DPB-flux/Ag detector.

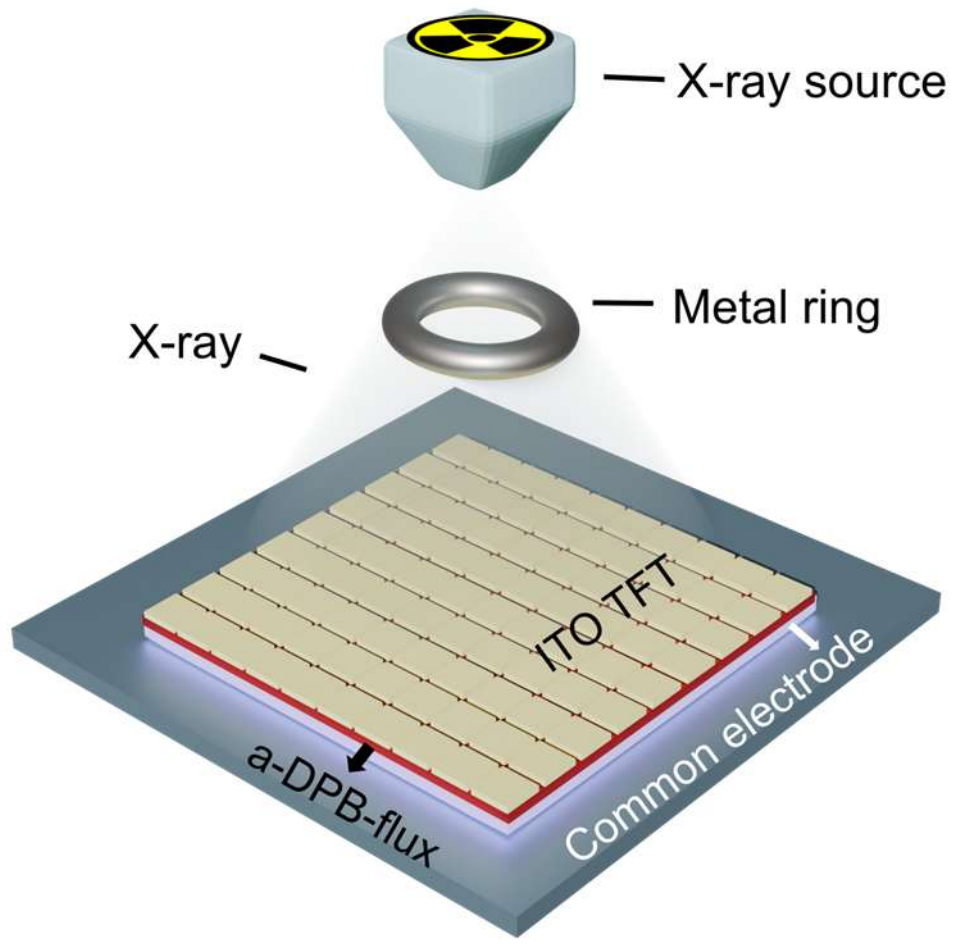

Supplementary Figure 40. Schematic diagram of X-ray the imaging experiment operated by the a-DPB-flux detector.

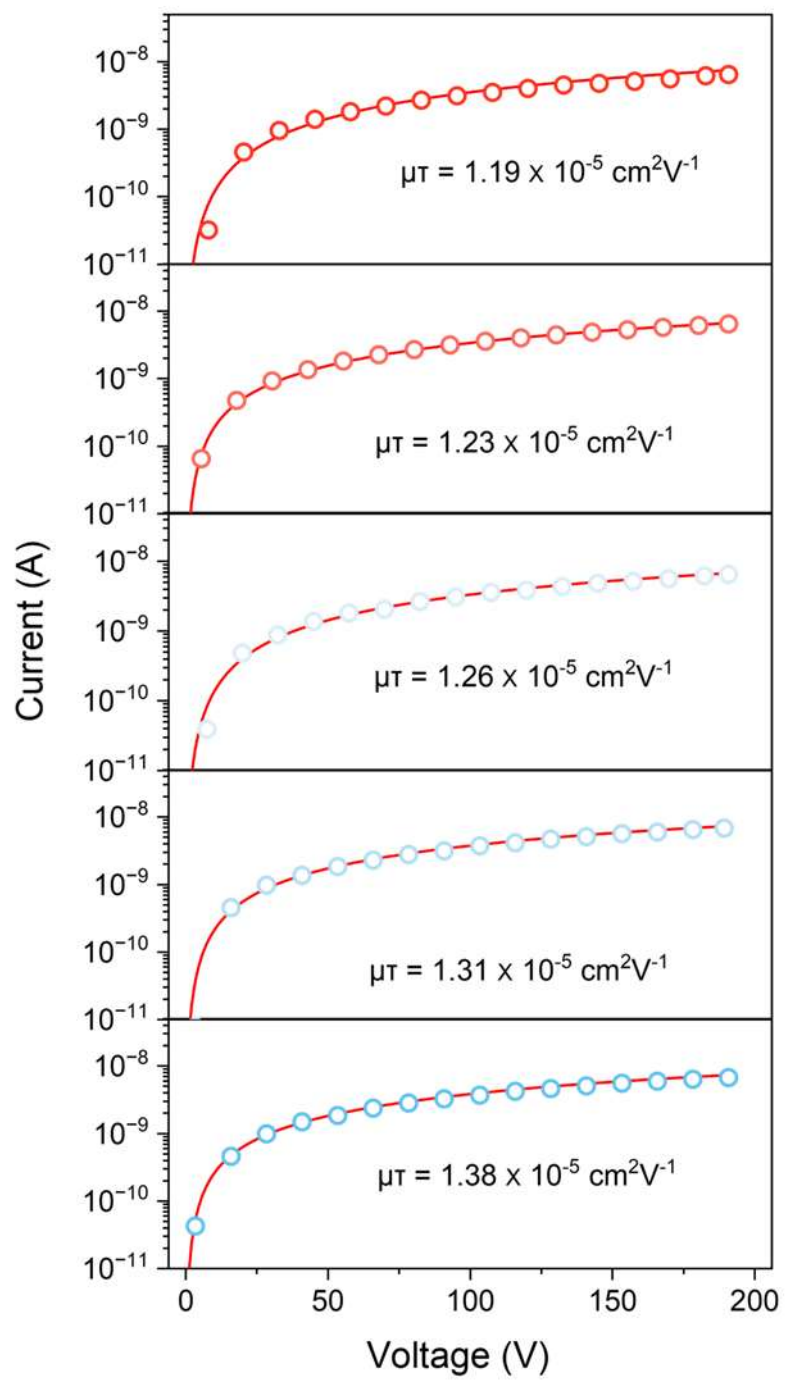

Supplementary Figure 41. The $\mu\tau$ parameter for the a-DPB-flux glass device over melt-processing cycles from 1 to 5.

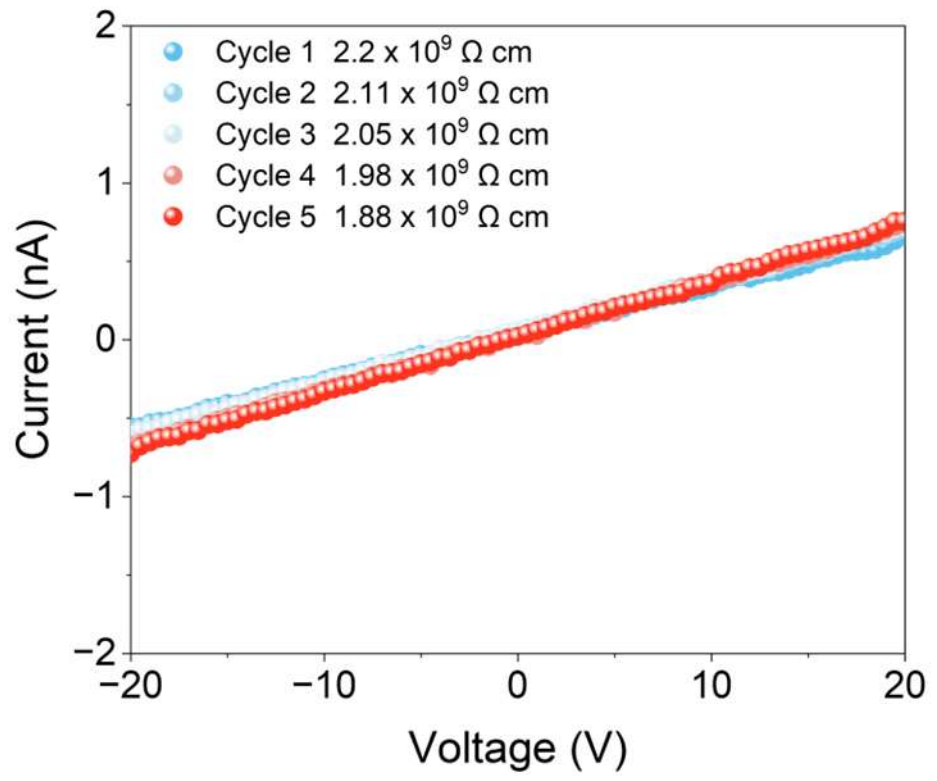

Supplementary Figure 42. The resistivity for the a-DPB-flux glass device over melt-processing cycles from 1 to 5.

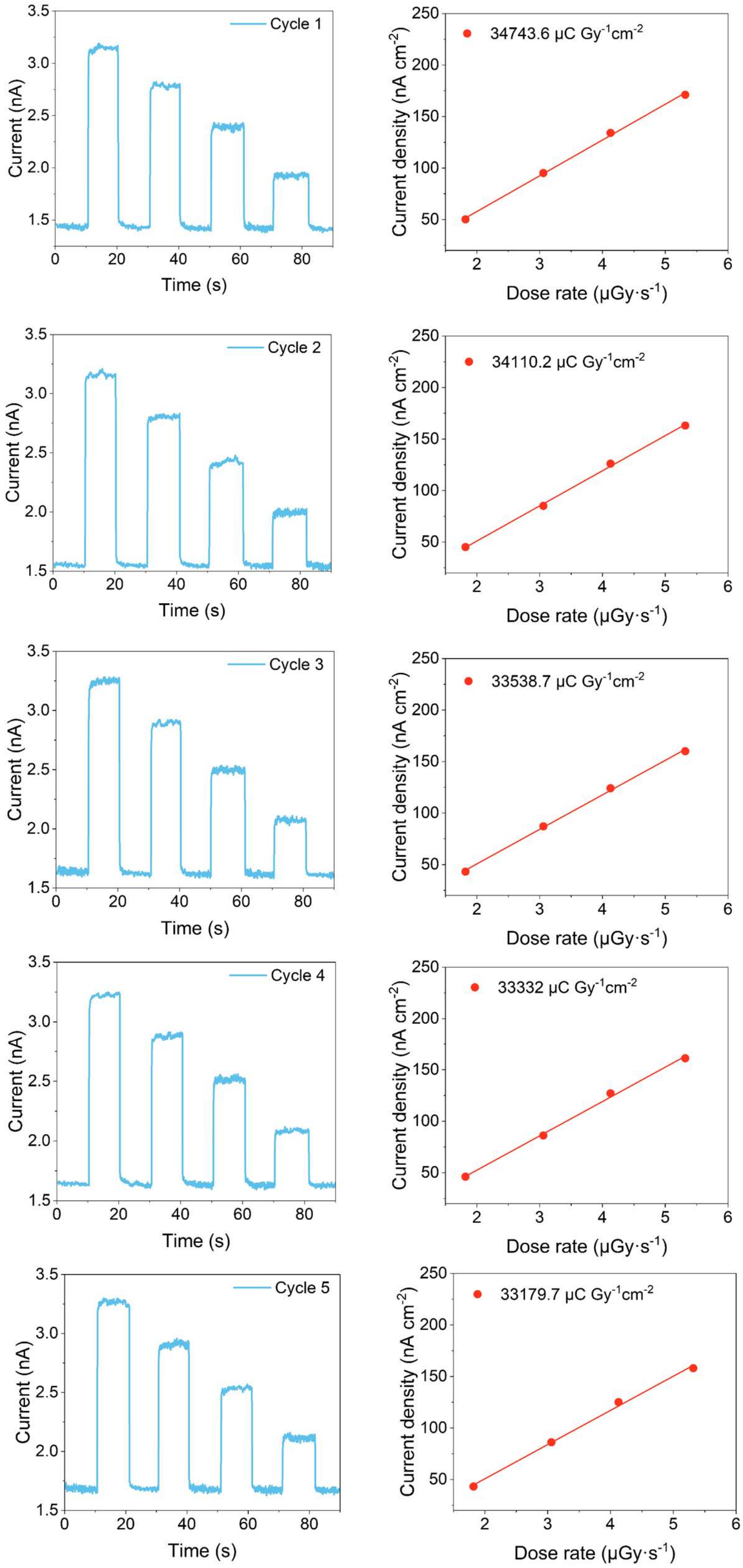

Supplementary Figure 43. The sensitivity for the a-DPB-flux glass device over melt-processing cycles from 1 to 5.

Supplementary Note 1

The reaction between oxoacid anions from the fluxes and hydrogen ions (H^+) from the organic cations is an acid-base neutralization reaction, reforming the original oxoacid. However, at elevated temperatures, these oxoacids degrade into volatile products. In an open environment, the removal of these products favors the forward direction of the reaction. The deprotonation induced by the acid-base neutralization reaction is most critical during the heating/melting process, whereas recombination upon cooling is limited by the rapid structural arrest of the glass and the local coordination of Li^+ with deprotonated organic/halide sites. The reaction equations are shown as follows:

$$SO_4^{2-} + 2H^+ = SO_3\uparrow + H_2O\uparrow\ (> 330\ °C)$$

$$2NO_3^- + 2H^+ = NO_2\uparrow + O_2\uparrow + H_2O\uparrow\ (> 80\ °C)$$

$$CO_3^{2-} + 2H^+ = CO_2\uparrow + H_2O\uparrow\ \text{(Room temperature)}$$

$$OH^- + H^+ = H_2O\uparrow\ (>100\ °C)$$

Supplementary Note 2

The thermodynamic process of solid-liquid phase transition is generally associated with enthalpy and entropy changes, as described by the relationship $T_m = \Delta H_{fus}/\Delta S_{fus}$, where ΔH_{fus} and ΔS_{fus} represent the enthalpy and entropy of fusion, respectively. Lowering T_m requires decreasing ΔH_{fus} or increasing ΔS_{fus}. The ΔH_{fus} involves intermolecular interactions in both solid and liquid phases, including Coulombic interactions, coordinate bonds, hydrogen bonding, van der Waals forces, and steric repulsion. The ΔS_{fus} comprises contributions from solid-state entropy (including configurational, vibrational, and rotational components) and liquid-state entropy (with an additional translational contribution).

In the case of DPB and DPB-flux, the ΔH_{fus} values were obtained by integrating the DSC curve, and the ΔS_{fus} values were calculated by the Gibbs free energy formula. The results show that the decrease in ΔH_{fus} and the increase in ΔS_{fus} after doping have led to a reduction in T_m.

Reference

1 Zhuang R., *et al*. Highly sensitive X-ray detector made of layered perovskite-like $(NH_4)_3Bi_2I_9$ single crystal with anisotropic response. *Nat. Photonics* **13**, 602-608 (2019).

2 Wang J., *et al*. Nonstoichiometry promoted solventless recrystallization of a thick and compact $CsPbBr_3$ film for real-time dynamic X-ray imaging. *Adv. Sci.* **11**, 2407314 (2024).

3 Wei W., *et al*. Monolithic integration of hybrid perovskite single crystals with heterogenous substrate for highly sensitive X-ray imaging. *Nat. Photonics* **11**, 315-321 (2017).

4 Chu D., *et al*. Stable and ultrasensitive X-ray detectors based on oriented single-crystal perovskite rods. *Adv. Mater.* **37**, 2500101 (2025).

5 Pan W., *et al*. $Cs_2AgBiBr_6$ single-crystal X-ray detectors with a low detection limit. *Nat. Photonics* **11**, 726-732 (2017).

6 Xu X., *et al*. Sequential growth of 2D/3D double-layer perovskite films with superior X-ray detection performance. *Adv. Sci*. **8**, 2102730 (2021).

7 He X., *et al*. Quasi-2D perovskite thick film for X-ray detection with low detection limit. *Adv. Funct. Mater.* **32**, 2109458 (2021).

8 Ma C., *et al*. Centimeter-sized 2D perovskitoid single crystals for efficient X-ray photoresponsivity. *Chem. Mater.* **34**, 1699-1709 (2022).

9 Li H., *et al*. Sensitive and stable 2D perovskite single-crystal X-ray detectors enabled by a supramolecular anchor. *Adv. Mater.* **32**, 2003790 (2020).

10 Dai H., *et al*. Building high-density polar hybrid perovskites via intercalation of Cs^+ and aromatic diamine for passive X-ray detection. *ACS Appl. Mater. Interfaces* **16**, 42372-42379 (2024).

11 Feng X., *et al*. Ligand-assisted growth of 2D perovskite single crystal for highly sensitive X-ray detectors. *Adv. Funct. Mater.* **34**, 2402166 (2024).

12 Zhong H., *et al*. Multiple interlayer interactions enable highly stable X-ray detection in 2D hybrid perovskites. *JACS Au* **4**, 2393-2402 (2024).

13 Wu W., *et al*. Unprecedented mixed low-dimensional metal halide double perovskite for stable and efficient X-ray detection. *Laser Photonics Rev.* **19**, e00276 (2025).

14 Liu D., *et al*. Quasi-one-dimensional perovskite single crystals enabling decoupled ionic-electronic transport for sensitive and stable X-ray detection. *Angew. Chem.*

Int. Ed. **65**, e24500 (2026).

15 Wu W., *et al*. Spontaneous cooling enables high-quality perovskite wafers for high-sensitivity X-ray detectors with a low-detection limit. *Adv. Sci*. **11**, 2410303 (2024).

16 Xu Y., *et al*. Evaporation crystallization of zero-dimensional guanidinium bismuth iodide perovskite single crystal for X-ray detection. *Inorg. Chem. Front.* **9**, 494-500 (2022).

17 Kasap S., *et al*. Amorphous selenium and its alloys from early xeroradiography to high resolution X-ray image detectors and ultrasensitive imaging tubes. *Phys. Status Solidi B* **246**, 1794-1805 (2009).

18 Kasap S. O., *et al*. X-ray sensitivity of photoconductors: application to stabilized a-Se. *J. Phys. D: Appl. Phys.* **33**, 2853-2865 (2000).

19 Tokuda S., *et al*. Preparation and characterization of polycrystalline CdZnTe films for large-area, high-sensitivity X-ray detectors. *J. Mater. Sci. Mater. Electron.* **15**, 1-8 (2004).

20 Xu Y., *et al*. Tailoring multi-phenyl ring cation for stable scalable hybrid bismuth iodide amorphous film: enabling record sensitivity and high-performance X-ray array imaging. *Adv. Mater*. **36**, 2406128 (2024).

21 Zhu Y., *et al*. One-dimensional hybrid metal halide with polymer-like flexible inorganic chains and glass-forming ability. *J. Am. Chem. Soc*. **147**, 35627-35637 (2025).
