# Supplementary material for: Deprotonation-mediated vitrification of organic-inorganic hybrid perovskites
Source: Nat Commun. 2026 Aug 17;17:9872. doi: 10.1038/s41467-026-76712-z (PMC13578220; doi:10.1038/s41467-026-76712-z)
Supplement: Supplementary file 2 [file 41467_2026_76712_MOESM2_ESM.pdf]

nature portfolio

Peer Review File

Deprotonation-mediated vitrification of organic-inorganic hybrid perovskites

Corresponding Author: Professor Shuang Yang

This file contains all reviewer reports in order by version, followed by all author rebuttals in order by version.

Attachments originally included by the reviewers as part of their assessment can be found at the end of this file.

Version 0:

Reviewer comments:

Reviewer #1

(Remarks to the Author)
This manuscript reports a novel "deprotonation-mediated" strategy to achieve the glassy state of various organic-inorganic hybrid perovskites (OIHPs). By introducing alkali metal hydroxide fluxes (e.g., LiOH), the authors successfully expanded the processing window from negative to positive values for several "non-meltable" perovskite systems, such as $(BA)_2PbI_4$. The resulting glassy semiconductors exhibit high X-ray sensitivity and excellent integration compatibility with commercial TFT backplanes. However, several critical scientific questions regarding material isotropy, defect chemistry, and the role of residual ions must be addressed before recommending publication.

1. While the authors provide XRD patterns and TEM images to confirm the amorphous nature of the glass, these techniques only probe the local or microscopic structure. For X-ray imaging applications, macroscopic isotropy is vital to ensure uniform charge transport across large areas.
2. Dark current analysis and electrical hysteresis: The reported X-ray sensitivity is impressive, but the report lacks detailed analysis of the dark current density and electrical hysteresis. Please provide cyclic I-V curves (forward and reverse scans) under high electric fields (up to 500 V/mm), and discuss whether the trap states in the glassy matrix or the migration of residual ions lead to detectable hysteresis, which would be detrimental to real-time imaging resolution.
3. Role of Residual Li^+ and Charge Neutrality: The "partial deprotonation" of organic ammonium cations (e.g. DGA^+) converts them into neutral molecules. To maintain the charge neutrality of the framework, the loss of H^+ must be compensated.
1) Please quantify the residual Li/Pb atomic ratio using quantitative techniques.
2) Discuss the coordination environment of Li^+ within the glass. Is Li^+ acting as a structural stabilizer or a mobile interstitial defect?
3) Given the high mobility of Li^+ in perovskite lattices, provide stability data for the dark current over time under continuous bias to evaluate ion-migration-induced baseline drift.
4. Point Defects and Processing Temperature
The authors claim that the flux reduces the processing temperature; however, the range of 170-270oC is still considered high for halide perovskites. Current literature suggests that halide vacancies (V_Br, V_I) and organic cation vacancies form readily above 150oC. Please discuss the impact of these thermally induced point defects on the μτ product. Are there self-passivation mechanisms within the glassy state that mitigate the negative effects of these defects?
5. Selection Rules and Dimensionality Constraints
The flux-mediated strategy appears highly effective for 1D and 2D systems but its applicability to 3D perovskites (e.g., $MAPbI_3$) or high-n 2D perovskites remains unclear.
1) Please discuss the "selection rules" for the organic ligands. Is the reaction strictly dependent on the pKa or other metrics of the ammonium group or the steric hindrance of the cation?
2) Explain why the rigid 3D corner-sharing framework might resist this deprotonation-induced vitrification compared to more flexible 1D/2D structures. In addition, the authors should add more discussions on the limitations of the current strategy.
6. Photoluminescence (PL) Spectroscopy as a Diagnostic Tool
PL spectra are highly sensitive to the defect density and electronic landscape of semiconductors. Please provide a comparative PL analysis of the crystalline perovskites, the glass with flux, and (if possible) the glass without flux after the same heat treatment. A shift in the emission peak or a change in the PLQY would provide deeper insights into the "damage-

free" nature of the deprotonation process and the role of LiOH in passivating or inducing trap states.

Reviewer #2

(Remarks to the Author)
Please find attached document with my comments and suggestions.

Reviewer #3

(Remarks to the Author)
This article reports on the fabrication of organic–inorganic hybrid perovskite glasses via a flux-assisted strategy, which lowers the melting point through a partial deprotonation mechanism. This strategy enables the preparation of high-quality perovskite glasses, and demonstrates broad universality across different material systems. Overall, this work holds significant implications for the synthetic chemistry of perovskite glass and recyclable optoelectronics. The manuscript is well prepared, and the results are comprehensive. It can be considered for publication after the following issues are resolved.

1. This study reduces the melting point through flux doping, which, unlike the organic component directed modification strategy with its inherent limitations, demonstrates superior convenience and universality. Although the two strategies differ, both lead to a change in the melting temperature of the material. Do they share a similar mechanism of action? The authors are requested to provide further explanation.
2. Two non-melting systems, $(BA)_2PbI_4$ and $(DGA)PbI_4$, achieved low temperature melting behavior through this strategy, and their corresponding glass transition temperatures were detected, which is impressive. However, the manuscript does not provide direct evidence of the two glasses. Please include corresponding optical or scanning electron microscopy images.
3. During the mixing and grinding process, the flux LiOH may undergo a partial deprotonation reaction with DPB crystals, causing changes in the crystal structure. Please provide XRD patterns of DPB at different doping ratios and explain the results.
4. I am also concerned about the influence of the flux on the semiconductor properties of perovskite materials. Will lithium ions migrate during the operation of the device, thereby affecting the dark current and the long-term stability of the detector?
5. The X-ray detector based on a-DPB-flux demonstrates excellent detection sensitivity and exhibits stable dark current under high electric fields. It is recommended to quantify the dark current drift value in Figure 5g. Is this excellent stability related to the disordered structure of the glass material? Please provide supporting evidence and elaborate.
6. When reporting the device's sensitivity and detection limit, the average X-ray energy used in the experiment should also be considered and stated, as both sensitivity and detection limit clearly depend on the X-ray energy.
7. I found that the authors used the RAYSAFE X2 for X-ray calibration. Actually, this dosimeter is suitable for X-ray calibration over a photon energy range of 40–150 keV. Using it to calibrate the 40 kVp X-ray beam employed in this work would introduce significant uncertainty. The authors are recommended to recalibrate the X-ray dose rate using a proper dosimeter, such as a gas-ionization chamber, to ensure data reliability.
8. There are some errors in the description. In line 223 of the main text, "whereby approximately 75%~85% of the hydroxide ions participate in protonation" should be corrected to "whereby approximately 75%~85% of the hydroxide ions participate in deprotonation." Please make this correction.

Version 1:

Reviewer comments:

Reviewer #1

(Remarks to the Author)
The authors have addressed all my concerns. I recommend publication in its current form.

Reviewer #2

(Remarks to the Author)
The comments have been satisfactorily addressed by the authors including necessary modifications to the manuscript. I congratulate the authors on the great results and recommend publication.

Reviewer #3

(Remarks to the Author)
I have read the author's responses to my questions and to the queries raised by the other two reviewers, and the author has made thorough revisions. I have no further questions. This work is highly systematic and solid, and I support its publication.

Open Access This Peer Review File is licensed under a Creative Commons Attribution 4.0 International License, which permits use, sharing, adaptation, distribution and reproduction in any medium or format, as long as you give appropriate credit to the original author(s) and the source, provide a link to the Creative Commons license, and indicate if changes were made.
In cases where reviewers are anonymous, credit should be given to 'Anonymous Referee' and the source.
The images or other third party material in this Peer Review File are included in the article's Creative Commons license, unless indicated otherwise in a credit line to the material. If material is not included in the article's Creative Commons license and your intended use is not permitted by statutory regulation or exceeds the permitted use, you will need to obtain permission directly from the copyright holder.
To view a copy of this license, visit https://creativecommons.org/licenses/by/4.0/

Reviewer#1 (Remarks to the Author):

This manuscript reports a novel "deprotonation-mediated" strategy to achieve the glassy state of various organic-inorganic hybrid perovskites (OIHPs). By introducing alkali metal hydroxide fluxes (e.g., LiOH), the authors successfully expanded the processing window from negative to positive values for several "non-meltable" perovskite systems, such as $(BA)_2PbI_4$. The resulting glassy semiconductors exhibit high X-ray sensitivity and excellent integration compatibility with commercial TFT backplanes. However, several critical scientific questions regarding material isotropy, defect chemistry, and the role of residual ions must be addressed before recommending publication.

Response: We sincerely thank the reviewer for the positive assessment of our work and for identifying several important scientific issues that should be clarified. We have carefully considered each comment and have revised the manuscript and Supporting Information accordingly. In particular, we have added more experimental results and discussions concerning macroscopic uniformity, electrical hysteresis, residual Li^+ species, defect formation, and the applicability/limitations of the flux-mediated strategy. Detailed point-by-point responses are provided below.

Comment 1: While the authors provide XRD patterns and TEM images to confirm the amorphous nature of the glass, these techniques only probe the local or microscopic structure. For X-ray imaging applications, macroscopic isotropy is vital to ensure uniform charge transport across large areas.

Response: We appreciate the reviewer's insightful comment. We agree that microscopic evidence of amorphization alone is not sufficient to demonstrate the macroscopic uniformity required for X-ray imaging applications. To address this important point, we have added a statistical evaluation of the electrical uniformity of a large-area a-DPB-flux device in the revised Supporting Information (Figure R1). Specifically, a 4 cm diameter glass plate was fabricated and a 5 × 5 array of Ag electrodes was deposited on its surface. The resistivity values measured from all 25 pixels show very low standard deviation, indicating excellent device-scale uniformity. Above macroscopic electrical results support that the melt-processed glass possesses homogeneous transport properties within the entire device. We have added this discussion to the revised manuscript (page 12, line 345-348) as follows:

To evaluate its potential for large-area imaging, we fabricated a large-area (4 cm in diameter) a-DPB-flux device and deposited a 5 × 5 array of silver electrodes on its

surface (Figure S39). The resistivities measured at the 25 pixels exhibited excellent uniformity, which is beneficial for large-area flat-panel X-ray imaging.

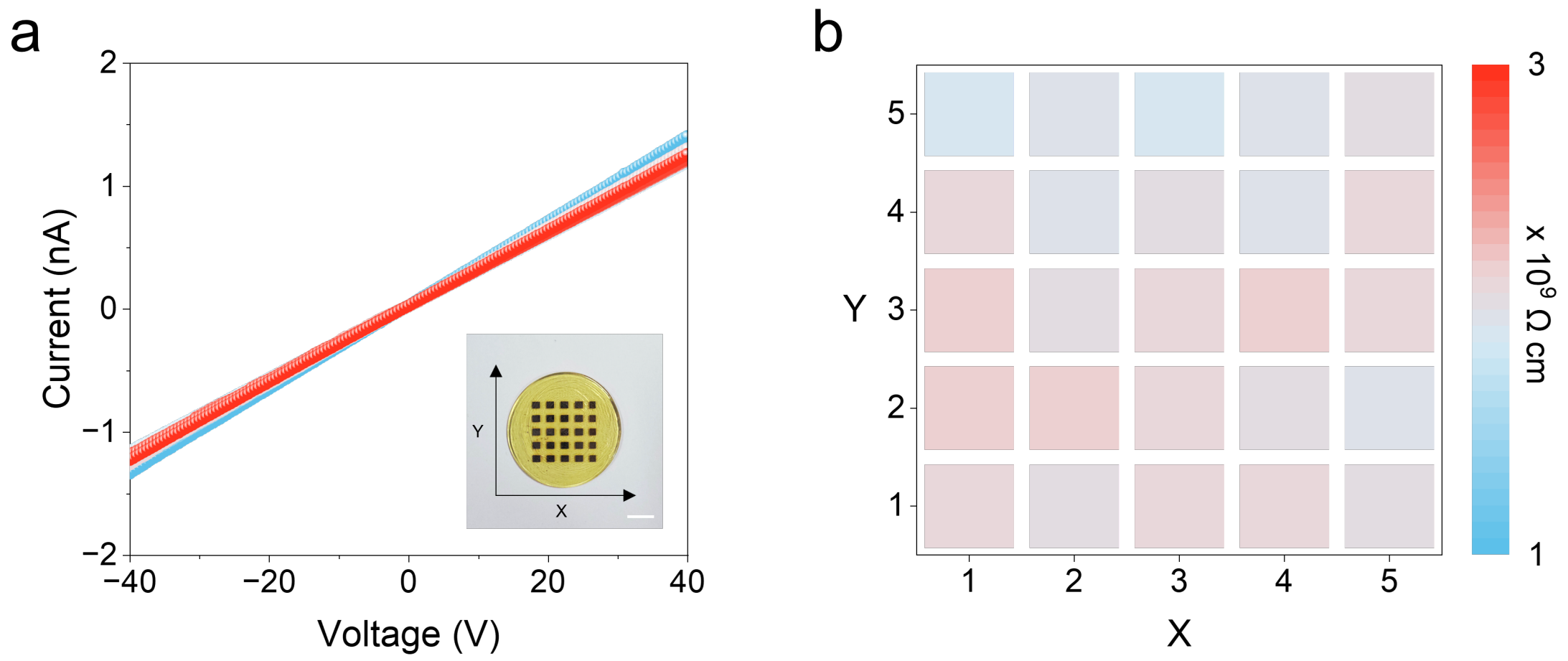

Figure R1. (a) Dark I-V curves of large-area a-DPB-flux device with a 5 × 5 array of metal electrodes. The inset shows the photograph of the device (Scale bar: 1 cm). (b) Statistical plot of resistivity for 25 individual pixels of the Ag/a-DPB-flux/Ag detector.

Comment 2: Dark current analysis and electrical hysteresis: The reported X-ray sensitivity is impressive, but the report lacks detailed analysis of the dark current density and electrical hysteresis. Please provide cyclic I-V curves (forward and reverse scans) under high electric fields (up to 500 V/mm), and discuss whether the trap states in the glassy matrix or the migration of residual ions lead to detectable hysteresis, which would be detrimental to real-time imaging resolution.

Response: We are grateful for the insightful suggestions. We agree that dark current and electrical hysteresis are critical parameters for real-time X-ray imaging, because that charge trapping or ion migration may lead to baseline drift and delayed signal readout.

Following the reviewer's advice, we have added cyclic dark I-V measurements of the Ag/a-DPB-flux/Ag device using forward and reverse voltage scans up to 500 V mm^{-1} (Figure R2a). We have added the figure to the revised Supporting Information as Figure S37. The dark-current density at 500 V mm^{-1} is 217.45 nA cm^{-2}, consistent with the high resistivity of 2.25 × 10^9 Ω cm reported in the manuscript. Importantly, the forward and reverse scans nearly overlap over the full field range, indicating the negligible effect of charge trapping or ion migration on device performance at the timescale. We further quantified the baseline drift under continuous 500 V mm^{-1} bias and obtained a drift rate of only 1.38 × 10^{-7} nA cm^{-1} s^{-1} V^{-1} (Figure R2b). The present data indicate that they do

not cause obvious hysteresis in the a-DPB-flux device.

In addition, the T_g of a-DPB-flux (52.5 °C) is above room temperature, suggesting that the glassy matrix is kinetically arrested during device operation. The stable dark current and X-ray response current further support this conclusion. We have incorporated this discussion into the revised manuscript (page 11, line 332-337) as follows:

The stability measurements in Figure 5g show the dark current drift of only 1.38 × 10^{-7} nA cm^{-1} s^{-1} V^{-1} under an electric field of 500 V mm^{-1}, as well as a stable photocurrent under continuous X-ray irradiation at 5.32 μGy_{air} s^{-1}. The overlapping of I-V curves measured by opposite scans of the a-DPB-flux device confirms its tolerance to electric field and X-ray radiation (Figure S37).

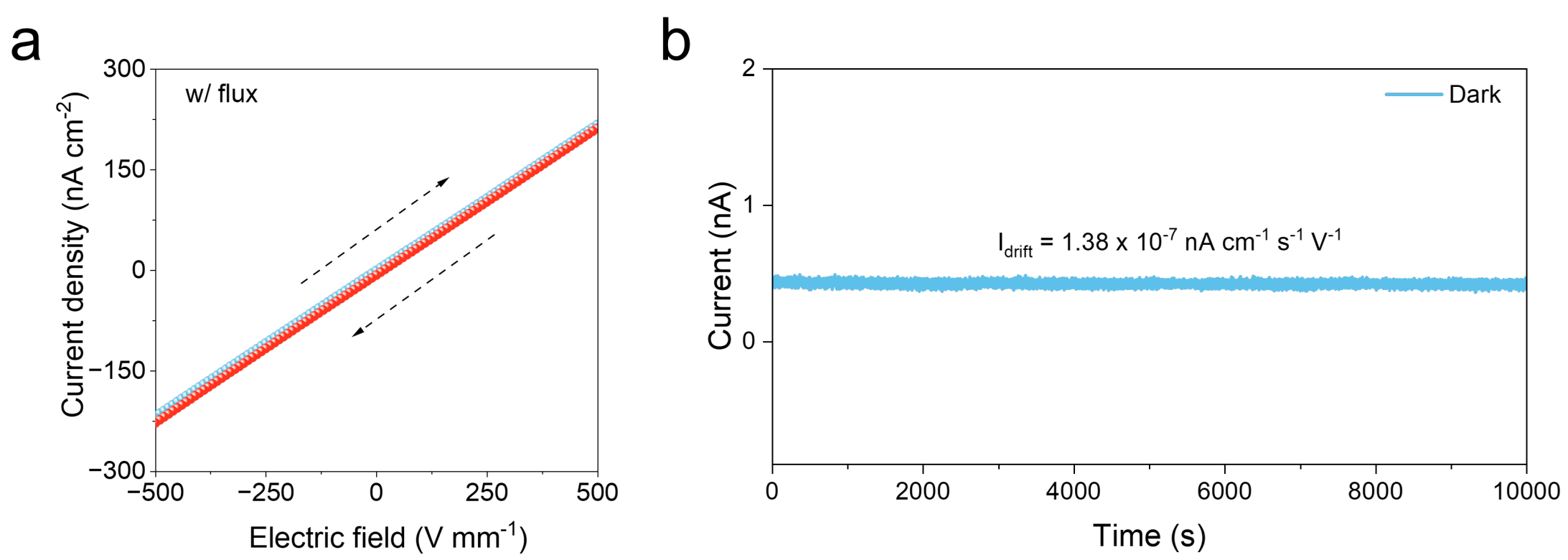

Figure R2. (a) Cyclic dark I-V curves (10 V s^{-1}) and (b) dark current stability assessment of the Ag/a-DPB-flux/Ag device under an electric field of 500 V mm^{-1}.

Comment 3: Role of Residual Li^+ and Charge Neutrality: The "partial deprotonation" of organic ammonium cations (e.g. DGA^+) converts them into neutral molecules. To maintain the charge neutrality of the framework, the loss of H^+ must be compensated.
1) Please quantify the residual Li/Pb atomic ratio using quantitative techniques.

Response: We really thank the reviewer's valuable comments and suggestions. We quantitatively determined the Li/Pb ratio by inductively coupled plasma optical emission spectrometry (ICP-OES) and obtained an atomic Li/Pb ratio of 0.28:29, which is close to the nominal value for a composition of 1.1 wt% LiOH (Figure R3).

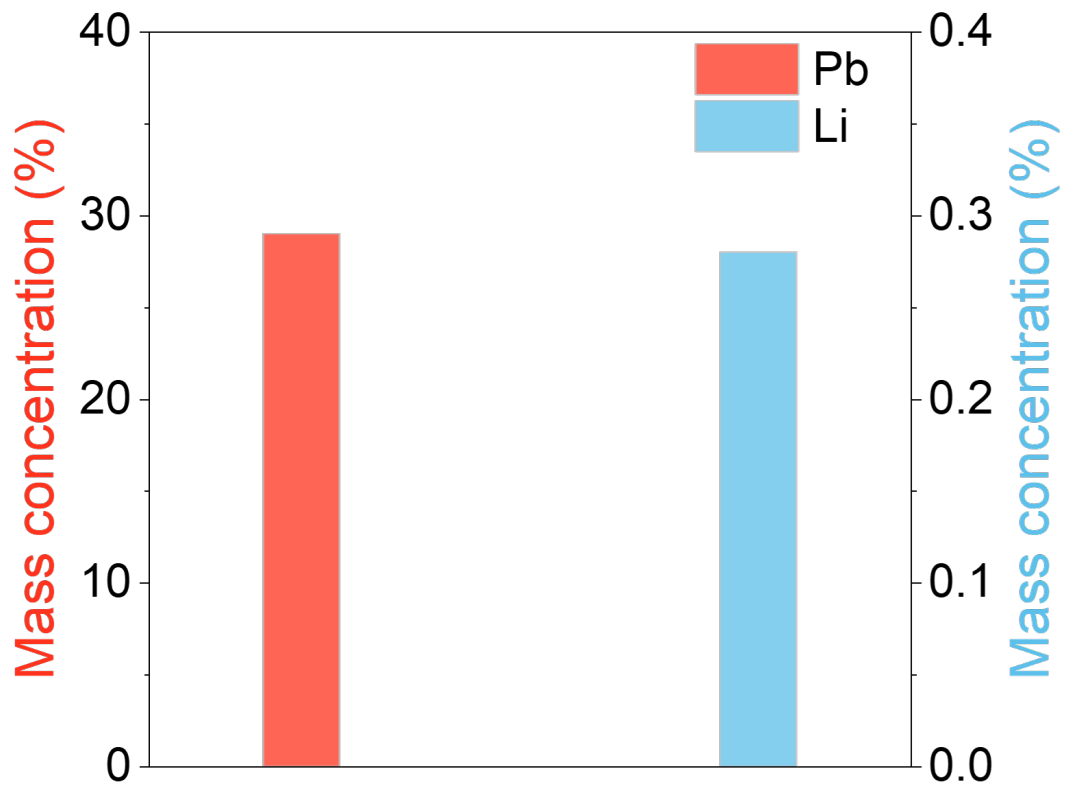

Figure R3. ICP-OES results of the Li/Pb ratio in a-DPB-flux.

2) Discuss the coordination environment of Li^+ within the glass. Is Li^+ acting as a structural stabilizer or a mobile interstitial defect?

Response: We really thank the reviewer's valuable comments and suggestions. Based on the DFT differential charge-density analysis, we propose that Li^+ is not a mobile interstitial defect in the glass (Figure R4a). Instead, Li^+ interacts with Br^- and deprotonated imino N sites at the deprotonated organic/halide interface. Such local coordination provides charge compensation for the partially deprotonated organic moieties and helps stabilize the disordered framework. Therefore, within the optimized flux concentration used in this work, Li^+ likely acts as a local charge-compensating/structural-stabilizing species rather than a highly mobile ionic defect. The forward and reverse scans almost overlap across the entire field range, indicating that charge trapping or ion migration has negligible impact on device performance over the measurement time scale (Figure R4b). We also quantified the baseline drift under a continuous bias of 500 V mm^{-1}, obtaining a drift rate of only 1.38×10^{-7} nA cm^{-1} s^{-1} V^{-1} (Figure R4c). These data indicate that Li^+ does not exhibit significant migration behavior in the a-DPB-flux device. We have clarified this interpretation in the revised manuscript (page 10, line 287-291) as follows:

Meanwhile, the Li^+ ions from LiOH flux that can provide charge compensation for the partially deprotonated organic moieties, closely interact with Br^- and deprotonated imino N sites at the deprotonated organic/halide interface in all models (Figure S32). Such a coordination environment would restrict the movement of Li^+.

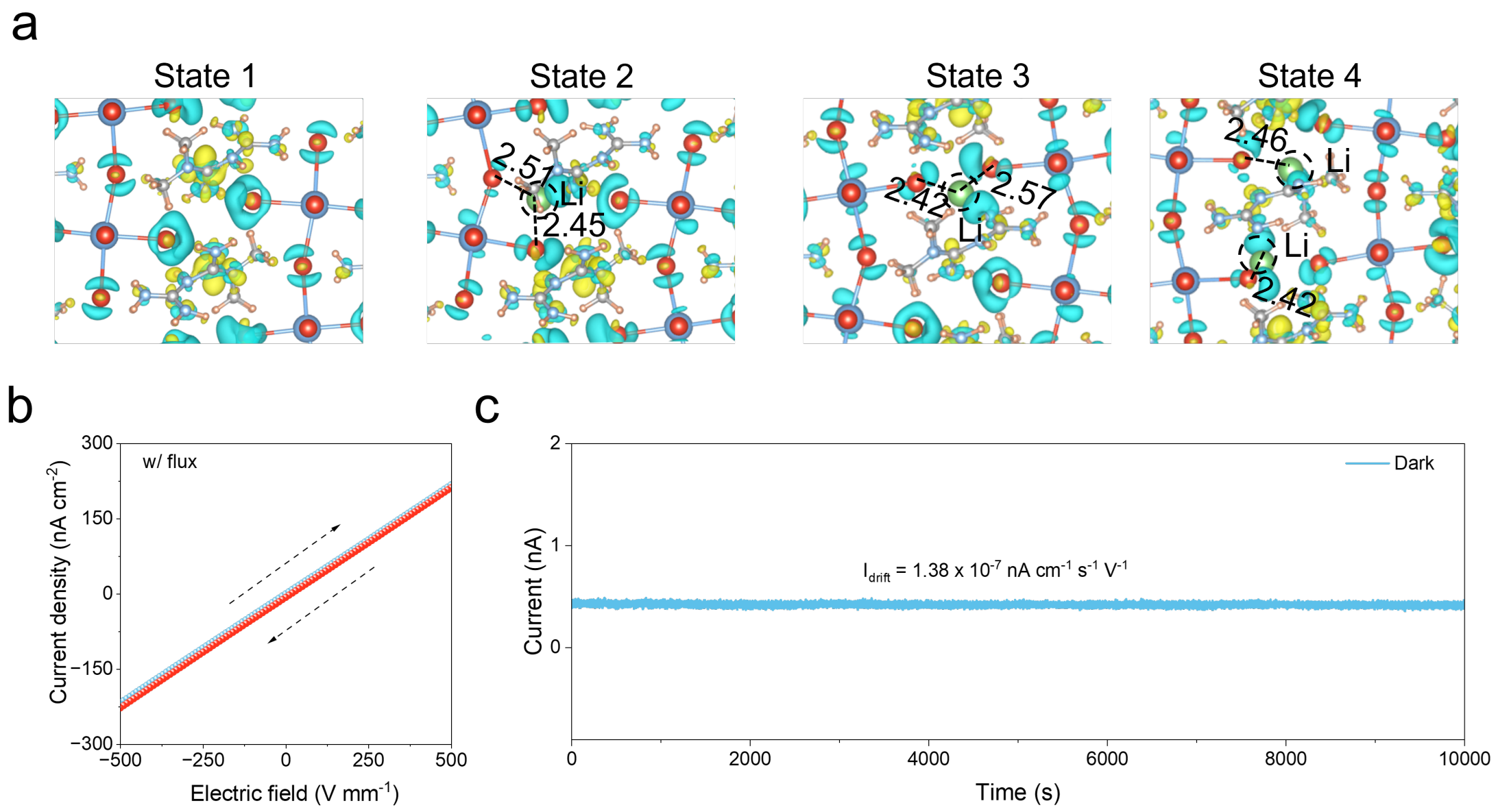

Figure R4. (a) Differential charge density in four states. (b) Cyclic dark I-V curves (10 V s^{-1}) and (c) dark current stability assessment of the Ag/a-DPB-flux/Ag device under an electric field of 500 V mm^{-1}.

3) Given the high mobility of Li^+ in perovskite lattices, provide stability data for the dark current over time under continuous bias to evaluate ion-migration-induced baseline drift.

Response: We thank the reviewer for this important suggestion. To directly evaluate the baseline drift potentially induced by ion migration, we added continuous bias dark current measurements under an electric field of 500 V mm^{-1} for a duration of 10,000 s. (Figure R5). The device exhibits a very small current drift of 1.38×10^{-7} nA cm^{-1} s^{-1} V^{-1} during this extended test. This result, together with the nearly hysteresis-free cyclic I-V curves, indicates that residual Li^+ ions are effectively immobilized in the glassy matrix under the operating condition. The inset of Figure 5g in the manuscript has been replaced with a revised figure (page 19).

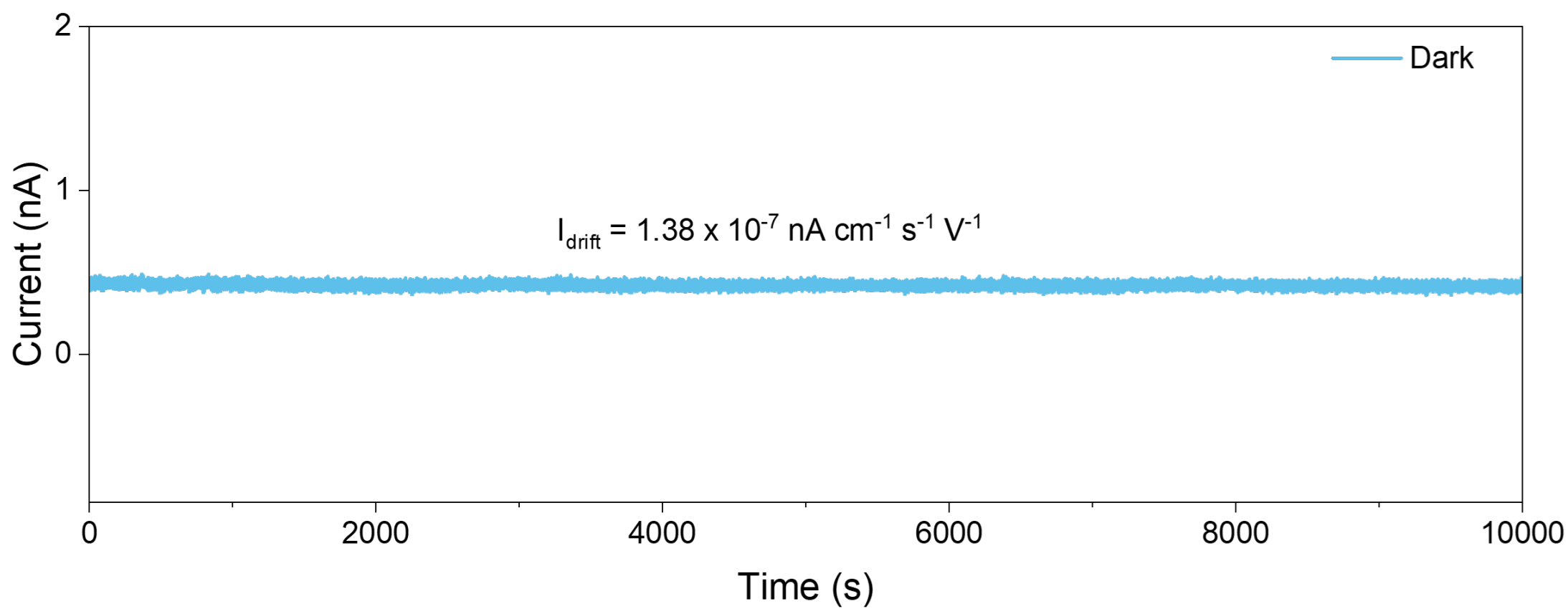

Figure R5. Dark current stability of Ag/a-DPB-flux/Ag devices under 500 V mm^{-1} continuous bias.

Comment 4: Point Defects and Processing Temperature

The authors claim that the flux reduces the processing temperature; however, the range of 170-270 °C is still considered high for halide perovskites. Current literature suggests that halide vacancies (V_Br, V_I) and organic cation vacancies form readily above 150 °C. Please discuss the impact of these thermally induced point defects on the $\mu\tau$ product. Are there self-passivation mechanisms within the glassy state that mitigate the negative effects of these defects?

Response: We are grateful for the insightful suggestions. We agree that thermally induced point defects may form in halide perovskites at elevated temperatures and can influence carrier transport. To clarify this issue, we have added $\mu\tau$ product measurements for DPB, DPB-(230 °C), and flux-free a-DPB (Figure R6), and compared them with a-DPB-flux. The $\mu\tau$ product decreases from 3.15 × 10^{-5} cm^2 V^{-1} for pristine DPB to 1.83 × 10^{-5} cm^2 V^{-1} after heat treatment at 230 °C (DPB did not melt at this temperature), confirming that thermal exposure can indeed introduce transport-limiting defects. Flux-free a-DPB shows a much lower $\mu\tau$ product of 6.57 × 10^{-6} cm^2 V^{-1}, which we attribute to more severe thermal decomposition and the formation of organic vacancies and other defect species during direct melting. In contrast, a-DPB-flux maintains a $\mu\tau$ product of 1.42 × 10^{-5} cm^2 V^{-1}. This improvement does not mean that the flux completely eliminates heat-induced defects; rather, the flux lowers the melting temperature, shortens the required thermal exposure, and broadens the melt-processing window, thereby reducing the extent of decomposition.

Besides, we conclude that the negative impact of residual point defects is mitigated by several factors: (i) reduced processing temperature and residence time, (ii) the defect-tolerant electronic structure of the Pb-halide framework, and (iii) the absence of grain-boundary migration pathways in the glass[1-4]. The high resistivity, stable dark current, and reproducible sensitivity after repeated processing cycles further indicate that the relatively low-temperature processing does not introduce high density of trap states.

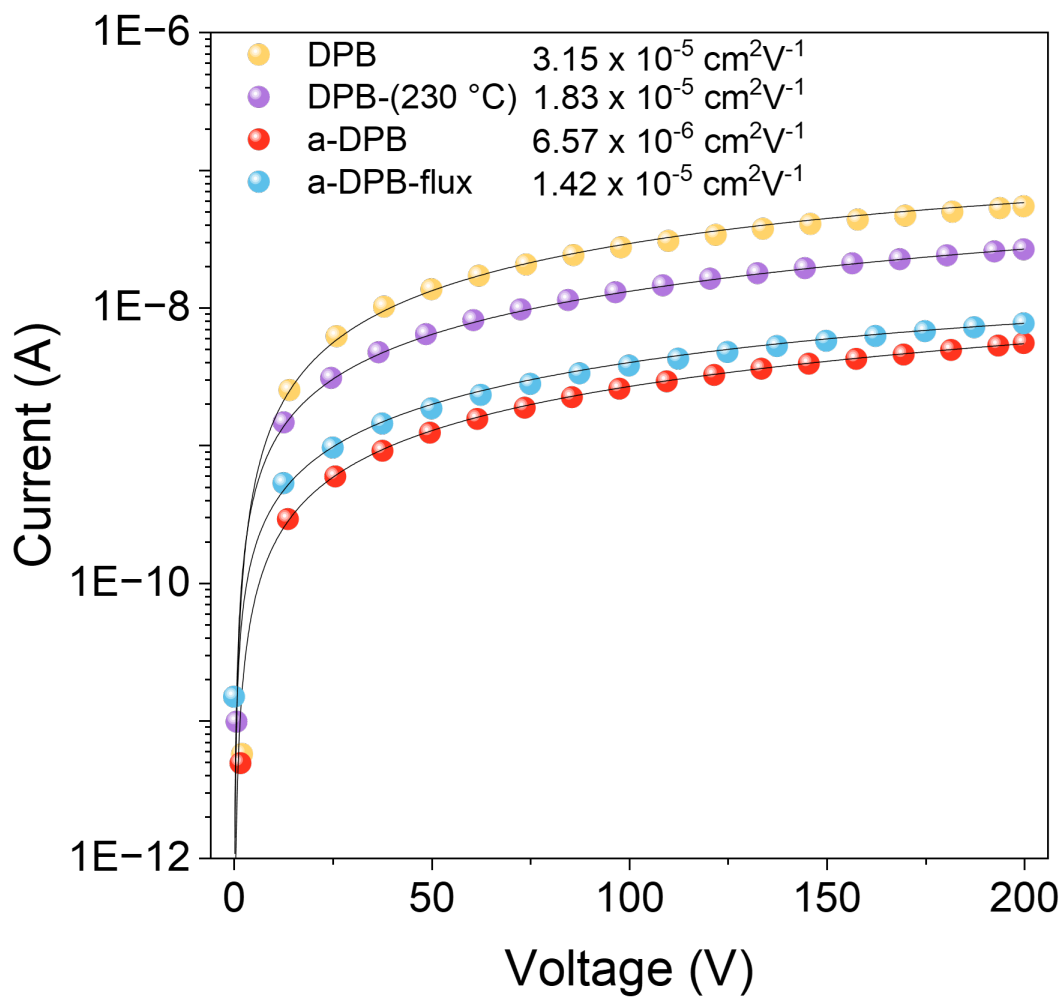

Figure R6. Photoconductivity characterization of DPB, DPB-(230 °C), a-DPB and a-DPB-flux devices.

Comment 5: Selection Rules and Dimensionality Constraints

The flux-mediated strategy appears highly effective for 1D and 2D systems but its applicability to 3D perovskites (e.g., $MAPbI_3$) or high-n 2D perovskites remains unclear.

1) Please discuss the "selection rules" for the organic ligands. Is the reaction strictly dependent on the pKa or other metrics of the ammonium group or the steric hindrance of the cation?

Response: We really thank the reviewer's valuable comments and suggestions. We agree that the effectiveness of the flux cannot be described by single factors, like pKa, steric hindrance of the cation and other factors. Instead, it is governed by a combination of molecular and structural factors, including the acidity and accessibility of N–H protons, the density and geometry of organic-inorganic hydrogen bonds, the steric accessibility of the organic cation, the connectivity and flexibility of the inorganic framework, and the ability of the lattice to accommodate local charge/coordination changes without catastrophic decomposition. Related studies by Mitzi et al also indicate that meltability and glass formation in hybrid halides depend on multiple coupled structural factors[4]. Given the flux mainly weakens in the interactions between spacers and inorganic frameworks, we infer that our strategy should be more effective in perovskites with accessible ammonium/iminium protons and strong organic-inorganic interfacial interactions.

2) Explain why the rigid 3D corner-sharing framework might resist this deprotonation-induced vitrification compared to more flexible 1D/2D structures. In addition, the authors should add more discussions on the limitations of the current strategy.

Response: We appreciate the reviewer's suggestion. Low-dimensional 1D/2D hybrid

metal halides contain relatively soft organic-inorganic interfaces and discrete inorganic motifs. In these systems, the temperature difference between melting point and degradation temperature is relatively small. Therefore, partial deprotonation can weaken interfacial interactions and reduce the melting point to be lower than degradation temperature.

By contrast, 3D $APbX_3$ perovskites such as $FAPbI_3$ possess a continuous corner-sharing PbX_6 network and small organic cations confined within cuboctahedra cavities. Its degradation temperature is mostly less than 200 °C, while the melting point is higher than 350 °C. Although deprotonation can weaken the chemical interaction, the reduction of melting point by over 150 °C is still a big challenge. Similarly, high-n 2D perovskites contain a smaller fraction of organic spacer and more rigid inorganic slabs, which reduces the leverage of interfacial deprotonation. We have therefore moderated our claim of universality and explicitly state that the present strategy is most suitable for low-dimensional hybrid metal halides with accessible ammonium/iminium protons and substantial organic-inorganic interfacial interactions. Further optimization will be needed for high-n 2D or 3D perovskite systems in future studies.

Comment 6: Photoluminescence (PL) Spectroscopy as a Diagnostic Tool
PL spectra are highly sensitive to the defect density and electronic landscape of semiconductors. Please provide a comparative PL analysis of the crystalline perovskites, the glass with flux, and (if possible) the glass without flux after the same heat treatment. A shift in the emission peak or a change in the PLQY would provide deeper insights into the "damage-free" nature of the deprotonation process and the role of LiOH in passivating or inducing trap states.

Response: We thank the reviewer for this valuable suggestion. We agree that PL spectroscopy is a sensitive diagnostic tool for evaluating the defect landscape and electronic-structure changes associated with flux-mediated vitrification. Accordingly, we have added comparative steady-state PL spectra of DPB and EPB, including crystalline samples with and without flux as well as glass samples prepared under comparable thermal conditions (Figure R7). For the crystalline samples, LiOH addition causes a moderate decrease in PL intensity for both DPB and EPB, while the overall emission profiles remain largely preserved. This suggests that partial deprotonation and the accompanying local structural perturbation slightly modify the electronic environment of the ordered lattice and may introduce limited non-radiative recombination pathways. This observation is consistent with the role of LiOH in weakening organic-inorganic interactions and lowering the melting temperature.

The glass samples exhibit a different trend in PL emission. For DPB, flux-free a-DPB shows an obvious change in the PL profile and weaker emission compared with a-DPB-flux. We attribute this behavior to the higher processing temperature required for flux-free vitrification, which more readily induces thermal decomposition and defect formation. In contrast, LiOH assisted vitrification lowers the melting temperature and reduces thermal exposure, thereby suppressing decomposition-related defect states and preserving a more favorable radiative recombination pathway in the glassy phase. For EPB glasses, both samples show very similar PL peak positions and spectral shapes, indicating that flux addition does not introduce additional electronic states after vitrification. One possible reason is that pure EPB do not degrade obviously during the melt-processing. Overall, the PL results support that LiOH-assisted deprotonation would not obviously damage the material during glass formation; rather, its main benefit is to reduce the thermal budget and suppress thermally induced degradation.

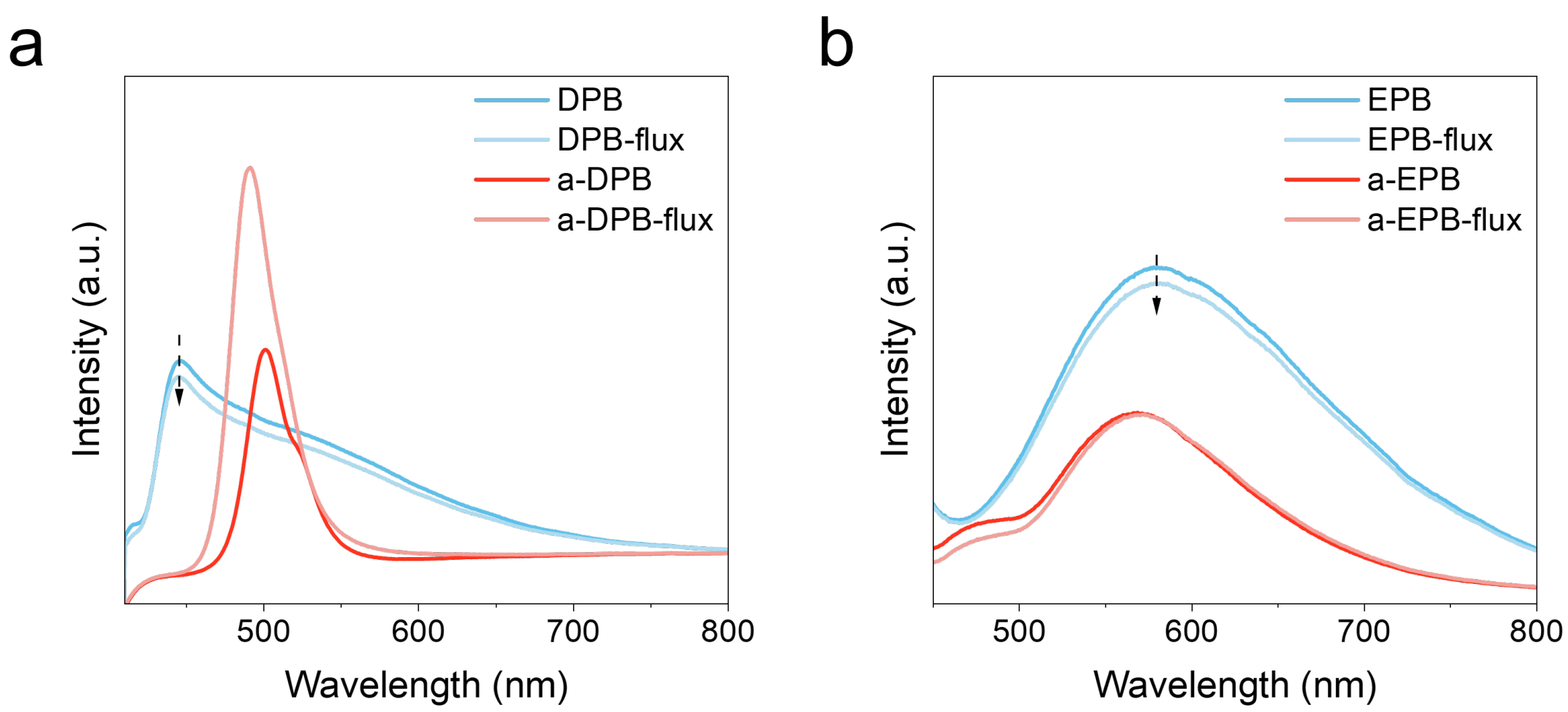

Figure R7. PL spectra of (a) DPB and (b) EPB samples.

Reviewer 2# (Remarks to the Author):

The manuscript reports a systematic reduction in melting points across a series of hybrid metal halides spanning multiple dimensionalities and structural motifs through the introduction of a flux additive. While flux-mediated melting-point reduction has been historically demonstrated for other classes of materials, its application to hybrid metal halides is, to the best of my knowledge, presented here for the first time. The authors support their proposed mechanism with comprehensive characterization and further demonstrate the practical relevance of this approach through radiation detection and imaging/screening applications.

Although reduced melting behavior in 2D hybrid perovskites has previously been reported for Ruddlesden-Popper (RP) phases, this work extends the concept to Dion–Jacobson (DJ) phases and further enables melting in compositions that were not previously known to exhibit melting. The ability to tune the thermal properties of hybrid metal halides in this manner is promising for the development of next-generation glassy optoelectronic devices. I commend the authors for the strong experimental results and the generally compelling discussion. I recommend the manuscript for publication after the following issues are addressed. Specific Comments and Suggestions.

Response: We sincerely thank the reviewer for the positive evaluation of our work and for the constructive and detailed suggestions. We have carefully revised the manuscript in response to each point, including clarifying the thermodynamic description, standardizing flux ratios, adding supporting experiments, moderating overstatements, and expanding the discussion of mechanism, device performance, and processing limitations. We believe that these revisions have improved both the accuracy and readability of the manuscript. Detailed responses are provided below.

Comment 1: In the first paragraph of the Results section and in the caption of Figure 1b, the statement “thermal energy is required to cross the barrier for glass transition” is scientifically inaccurate in this context. The discussion should refer to melting rather than glass formation. Please revise this here and elsewhere in the manuscript where applicable.

Response: We really appreciate the reviewer’s positive comments and valuable suggestions. We agree that the relevant energy barrier in this context should refer to melting or the crystal to liquid transition, rather than glass transition. We have therefore replaced the original phrase with “thermal energy required for melting” and “the energy barrier to reach the molten state” in the Results section, the Figure 1b caption, and other relevant locations throughout the manuscript. The revised text has been highlighted.

Comment 2: Molar ratios and weight ratios are used interchangeably throughout the manuscript, which may be confusing to readers. Please include a table clearly correlating molar ratios with the corresponding weight ratios for ease of reference.

Response: We appreciate the reviewer's helpful suggestion. We agree that using molar ratios and weight ratios in different sections could cause confusion. In the original manuscript, Figure 1c used a molar ratio for the comparative screening of 24 fluxes, whereas the optimized compositions in the later sections were described by weight ratio. To improve clarity, we have converted the molar ratios into the corresponding weight ratios and added a summary table in the Supporting Information (Table R1). We have also revised the relevant text in the main manuscript (page 4, line 96-97) and Supporting Information (page 3) to make the ratio definitions consistent and easier to follow.

Table R1. The mass ratio (wt%) of the flux for all samples in Figure 1c.

	OH^-	CO_3^{2-}	NO_3^-	SO_4^{2-}	F^-	Cl^-	Br^-	I^-
Li^+	1.1	3.38	3.15	5.03	1.19	1.94	3.97	6.12
Na^+	1.83	4.85	3.89	6.49	1.92	2.67	4.71	6.86
K^+	2.57	6.32	4.62	7.97	2.66	3.41	5.44	7.59

Comment 3: During melting of flux-added hybrids, water effusion is observed. Does this process also contribute to degradation of the hybrid materials? Please clarify and, if possible, discuss supporting evidence.

Response: We thank the reviewer for the valuable comment. We agree that water formation and evolution during acid base neutralization should be discussed explicitly. At the optimized flux concentration, the amount of generated water is stoichiometrically small and can be released during melt processing. As a quantitative example, 50 mg of DPB containing 0.55 mg LiOH is expected to generate only approximately 0.37 mg of water, which can volatilize at temperatures above 100 °C. To evaluate whether this amount of water could induce degradation, we performed a control experiment under semi-closed conditions (Figure R8). DPB was exposed to a much larger amount of water vapor generated from 40 mg water (approximately 100 times the calculated amount from LiOH neutralization) and held at 120 °C for 30 min, followed by cooling to room temperature. A parallel DPB/LiOH sample was treated under the same conditions. Powder XRD patterns show no significant peak shift or new

decomposition peaks in either sample, suggesting that the limited water evolution in the optimized flux-assisted process does not strongly degrade the hybrid perovskite materials.

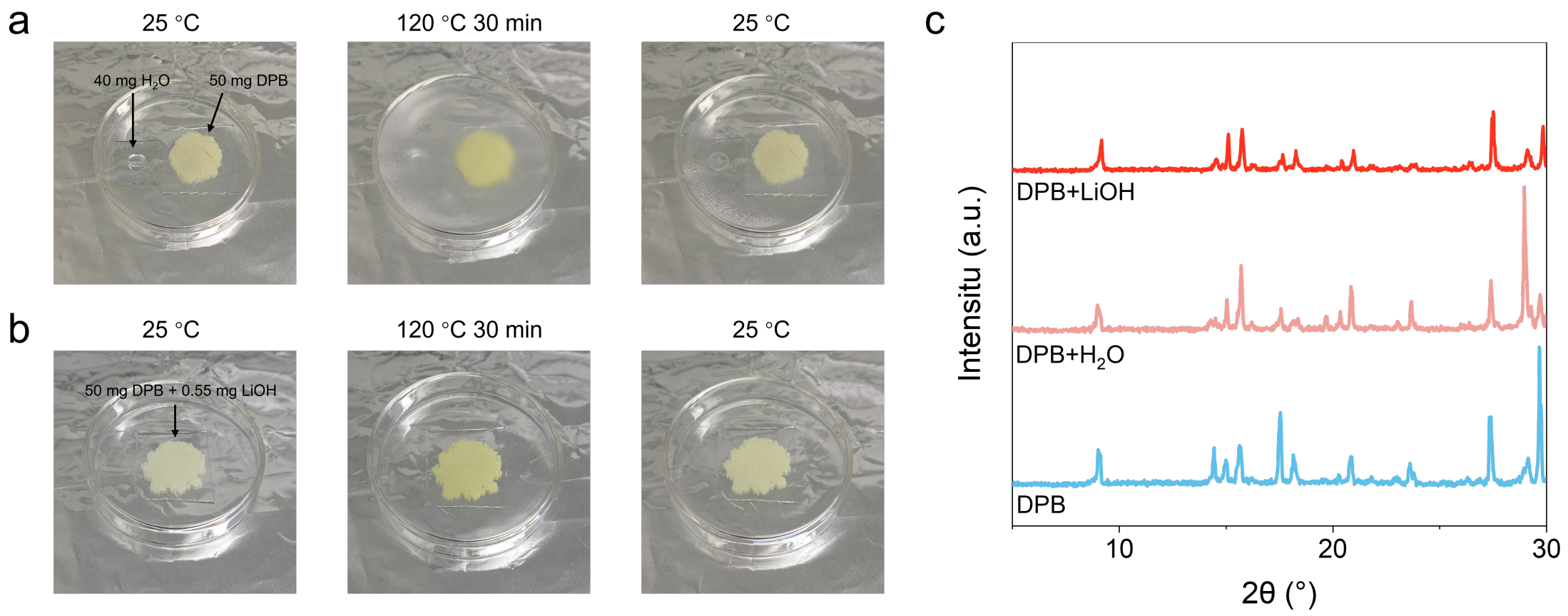

Figure R8. Photos of (a) 50 mg DPB with 40 mg water, and (b) 50 mg DPB with 0.55 mg LiOH, heated at 120 °C for 30 min under semi-closed conditions and then cooled to room temperature. (c) Powder XRD patterns of DPB powders cooled from the two conditions and an untreated DPB sample.

Comment 4: If the flux facilitates constituent mobility, as reflected by a reduction in T_g, what are the implications for charge transport properties in crystalline vs glassy phases, both with and without flux addition? A brief discussion would strengthen the manuscript.

Response: We appreciate the reviewer's insightful suggestion. We have conducted photoconductivity measurements of DPB, DPB-flux and a-DPB (Figure R9). We have added the figure to the revised Supporting Information as Figure S35. In the crystalline phase, charge transport can be more anisotropic because of crystal orientation and layered inorganic connectivity, and the pristine crystal therefore shows a relatively high $\mu\tau$ product of 3.15×10^{-5} cm^2 V^{-1}. After LiOH addition, the $\mu\tau$ product decreases to 2.36×10^{-5} cm^2 V^{-1}, suggesting that flux may introduce limited trap states in the ordered lattice. However, this effect remains modest, likely because the room-temperature grinding process only allows limited deprotonation before heating.

For the glassy samples, the situation is different. Although LiOH slightly lowers the T_g of DPB from 54 °C to 52.5 °C, the T_g remains above room temperature, so long-range ionic motion is kinetically suppressed during device operation. Flux-free a-DPB shows a lower $\mu\tau$ product of 6.57×10^{-6} cm^2 V^{-1} because it requires a higher processing temperature and therefore suffers more severe thermal decomposition. In contrast, the

optimized flux content indirectly improves charge transport in a-DPB-flux by reducing the processing temperature, suppressing decomposition-indued defects, and eliminating grain-boundary pathways. We have incorporated this balanced discussion into the revised manuscript (page 11, line 312-318) as follows:

By fitting photoconductivity data with the modified Hecht equation, we find that the addition of LiOH slightly reduces μτ product of the crystals, suggestive of the formation of trap states (Figure S35). In the glassy phase, a-DPB-flux achieves a μτ of 1.42×10^{-5} cm^2 V^{-1} *(Figure 5b), whereas a-DPB without flux shows a drastically lower μτ of* 6.57×10^{-6} cm^2 V^{-1} *due to thermal decomposition. Thus, the utilization of flux lowers the processing temperature to suppresses the thermal decomposition, and thereby benefits charge transport in a-DPB-flux.*

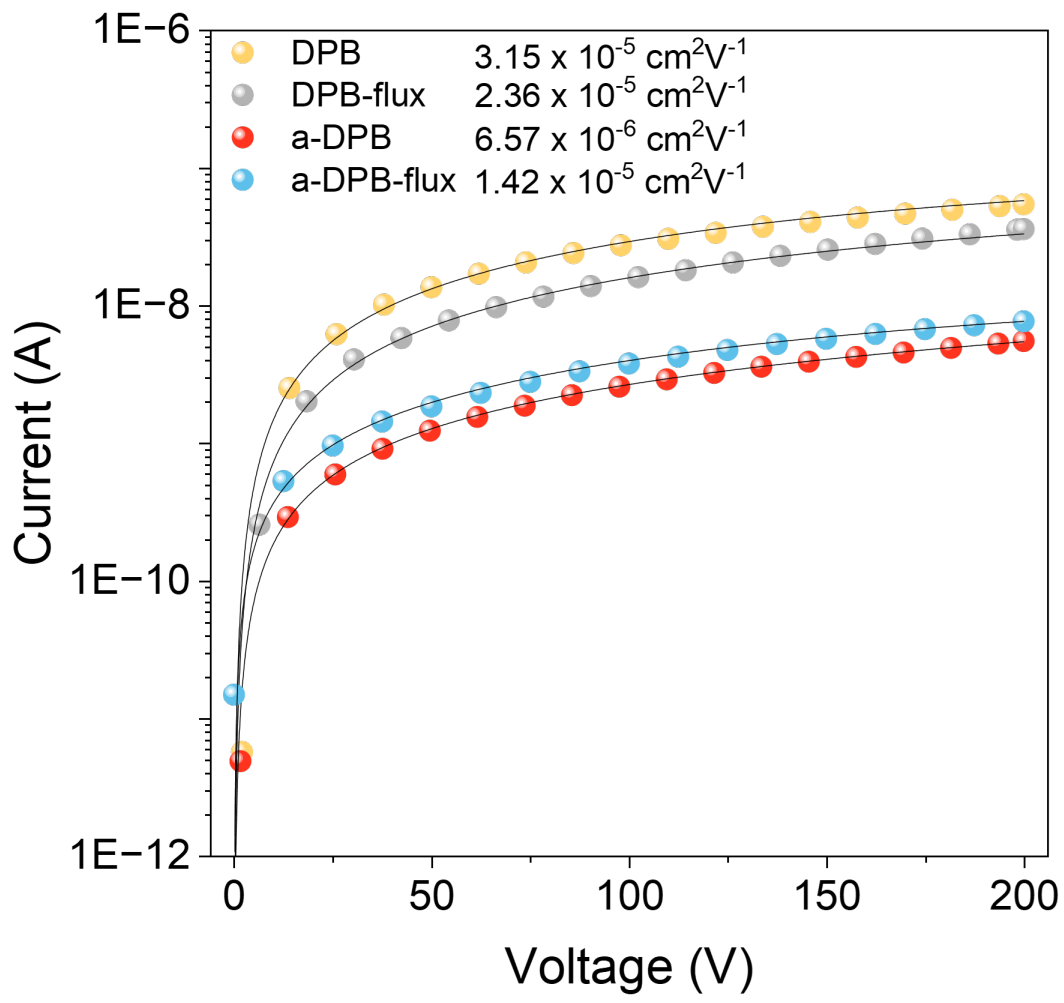

Figure R9. Photoconductivity characterization of DPB, DPB-flux, a-DPB and a-DPB-flux device.

Comment 5: The reported transmittance values of 73–87%, claimed as the highest ever reported for metal halide glasses, should be supported with appropriate references or moderated accordingly. Additionally, please clarify whether film or monolith thickness was accounted for when making this comparison.

Response: We thank the reviewer for the valuable comments. After referring a substantial amount of relevant literature, we found that most reports did not give detailed film thickness for the transmittance measurements. We have therefore adjusted the description in the text to avoid misleading readers.

We have therefore adjusted the wording in the text to avoid misleading readers. We have also clarified that the spectrum in Figure 2k was collected from a 0.5 mm thick glass sheet, whereas the photograph in Figure S17 shows a 2 mm thick bulk pieces. We

have incorporated this balanced discussion into the revised manuscript (page 6, line 160-162) as follows:

Critically, the usage of LiOH flux enhanced the transmittance of all glasses (approximately 500 μm thick, except a-DBI with narrow bandgap) to approximately 73%–87% at the wavelength region of 500 to 800 nm.

Comment 6: Please provide a reference supporting the assignment of the amine proton peak in the ^{1}H NMR data shown in Figure S25.

Response: We are grateful for the valuable suggestions. We have added the relevant references (refs 36-38 in the revised manuscript) and assigned the hydrogens to the ^{1}H NMR peaks according to the literature (Figure R10/Figure S25).

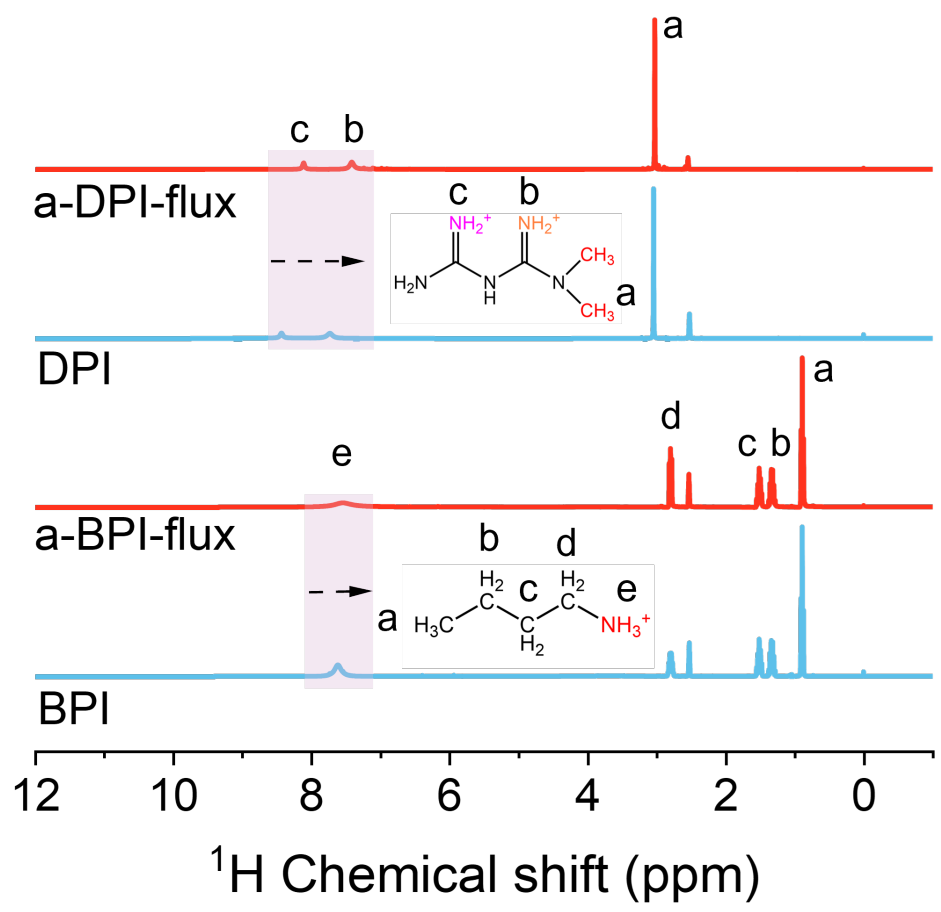

Figure R10. ^{1}H NMR spectra of crystalline and glassy BPI and DPI.

Comment 7: The manuscript states that minimized decomposition leads to very similar bandgaps in crystalline and glass phases (Figure S26). However, prior studies on hybrid halides typically report a pronounced difference in absorption onset between these phases (Refer Bennett et. al and Mitzi et. al.). Please discuss why such a contrast is not observed here.

Response: We thank the reviewer for the careful checking of our work. Figure S26 shows that BPI crystal and BPI glass exhibit similar optical bandgaps. We believe this relatively small difference arises from the flux-assisted process, which lowers the processing temperature and minimizes organic-component loss, thereby preserving the local Pb–I coordination motif that largely determines the absorption onset. In this case, vitrification mainly removes long-range ordering while retaining the local electronic building blocks. The reproducible melting and crystallization peaks observed over repeated heating/cooling cycles further support the stability of the Pb–I framework

during the reversible transition. By contrast, the larger absorption-onset shifts reported in earlier studies may originate from more extensive structural reorganization, partial decomposition, changes in coordination dimensionality, indirect bandgap absorption or stronger excitonic/packing effects during high-temperature melt quenching process[4-7]. We have revised the relevant sentence to avoid suggesting that similar bandgaps are universal for all hybrid-halide glass systems. We have incorporated this balanced discussion into the revised manuscript (page 7, line 198-202) as follows:

The minimization of BPI decomposition results in very similar bandgap of the crystalline and glass phases (Figure S26), which likely originates from the lower processing temperature and minimized loss of organic components that preserve the local Pb–I coordination motif.

Comment 8: The argument that flux addition disrupts the organic–inorganic interface, lowering the enthalpy of melting/fusion, is well articulated and supported by characterization. However, the additional claim that deprotonated organic cations provide increased molecular flexibility and entropy gain requires further validation and should be decoupled from the enthalpy argument. If direct evidence is not available, I recommend omitting this interpretation.

Response: We thank the reviewer for the valuable comments. Currently, it is hard to quantitatively analyze the molecular flexibility or entropy gain of the system. Therefore, we have removed the corresponding statement from the main text. The mechanistic discussion now focuses on the directly supported decrease in ΔH_{fus} and the weakening/disruption of organic-inorganic interfacial interactions induced by partial deprotonation.

Comment 9: For X-ray detection, the reported maximum sensitivity of 38,772.9 μC Gy_{air} cm^{-2} at 500 V/mm is described as top-tier for amorphous semiconductors. Please substantiate this claim by comparison with state-of-the-art materials (crystalline as well) and devices.

Response: We thank the reviewer for this important suggestion. To substantiate the performance comparison more rigorously, we have added a comparison table (Table R2) that, together with Figure 5e in the main text, summarizes the sensitivity, electric field, and minimum detection limit of representative amorphous semiconductors, single crystalline and polycrystalline perovskite materials of different dimensionalities, a-Se, a-Ga_2O_3, and other related X-ray detectors. We have included this table in the revised Supporting Information Table S6 (page 8). The a-DPB-flux device achieves a

sensitivity of 35674.1 μC Gy_{air}^{-1} cm^{-2} at 500 V mm^{-1} (This sensitivity was calculated after recalibrating the X-ray source, as requested by other reviewers), which is relatively high among amorphous semiconductor detectors under similar bias conditions.

Table R2. Summary of key performance parameters of representative perovskite-based X-ray detectors.

Type	Dimension	Electric field (V cm^{-1})	Sensitivity (μC Gy^{-1} cm^{-2})	LoD (nGy s^{-1})	Ref.
$(NH_4)_3Bi_2I_9$	2D	22 (parallel)	8200	210	(8)
$CsPbBr_3$	3D	4000	42000	136	(9)
$MAPbBr_3$	3D	4670	21000	100	(10)
$FAPbI_3$	3D	1000	216000	2	(11)
$Cs_2AgBiBr_6$	3D	250	105	59.7	(12)
$MAPbI_3$+$PEA_2MA_8Pb_9I_{28}$	3D	333	19500	480	(13)
$PEA_2MA_8Pb_9I_{28}$	2D	6,000	10860	69	(14)
$(3AP)PbCl_4$	2D	2500	792	1540	(15)
$(F\text{-}PEA)_2PbI_4$	2D	1333	3402	23	(16)
$(4\text{-}AP)CsPb_2I_8$	2D	500	1890	529	(17)
$CsPb_2Br_5$	2D	6250	8865.6	12.7	(18)
$(BPEA)_2PbI_4$	2D	800	1003	366	(19)
$(4\text{-}AP)_3AgBi_3Br_{20}$	1D	500 (parallel)	1567	38.1	(20)
$(C_4H_{14}N_2S_2)_6Pb_5I_{22}$ $4H_2O$	1D	500	142000	11.7	(21)
$Cs_3Cu_2I_5$	0D	125	3433	33.17	(22)
$(Gua)_3Bi_2I_9$	0D	8333	18.23	237.54	(23)
α-Se	amorphous	$0.5\text{-}1\times10^5$	~20-50	~5500	(24-25)
CdZnTe	/	2500	2400	50000	(26)
$MTP_3Bi_2I_9$	glass	4000	1600	19.69	(3)
$(4\text{-}EAMP)_2Pb_3Br_{10}$	glass	1000	8581.7	135	(27)
a-DPB-flux	glass	5000	35674.1	93.6	This work

Comment 10: DSC data show crystallization upon cooling for BPI, whereas monoliths form glasses. Please specify the cooling rates used in DSC measurements and during monolith processing to clearly distinguish between characterization conditions and

form-factor-dependent processing effects.

Response: We thank the reviewer for the valuable comment. We have now specified the cooling conditions in the revised Methods (page 23, line 566-568) and Supporting Information (page 24). The DSC measurements were performed using a heating rate and cooling rate of 10 °C min^{-1}. Under this relatively slow and controlled cooling condition, BPI-flux can crystallize during the cooling scan. In contrast, the monolithic glass pieces were prepared by melting the material in a vacuum-sealed quartz tube followed by rapid quenching in liquid nitrogen. This rapid quenching bypasses crystallization and kinetically traps diverse species into the glassy state. We have revised the relevant sections to clearly distinguish the DSC characterization conditions from the bulk-glass processing conditions.

Comment 11: A shift in the C=N stretching mode in FTIR is attributed to flux addition in the glassy state and correlated to the corresponding NMR peak splitting (Figures 3C and S29), which instead compares crystalline vs glass states. To validate this correlation, FTIR spectra comparing crystalline vs glass states should also be provided and discussed.

Response: We thank the reviewer for the valuable comment. We have added FTIR spectra of DPB, DPB-flux, a-DPB, and a-DPB-flux for direct comparison (Figure R11). These data allow us to separate two effects: (i) the crystal-to-glass transition, which produces spectral changes associated with structural disorder, and (ii) LiOH induced deprotonation, which modifies the C=N and N–H related vibrational modes through electron-density redistribution.

In the crystalline samples, LiOH-induced deprotonation mainly decreases the relative intensities of the C=N and N–H vibrations compared with C–N and NH_2, without causing a pronounced stretching shift, likely because that the deprotonation reaction during room-temperature grinding is diffusion-limited. In the glassy samples, the C=N related feature is shifted, consistent with stronger structural reorganization and charge redistribution after thermal vitrification. We have revised the caption and discussion of Supporting Information Figure S29 (page 25) to avoid over-correlating features that arise from different structural states.

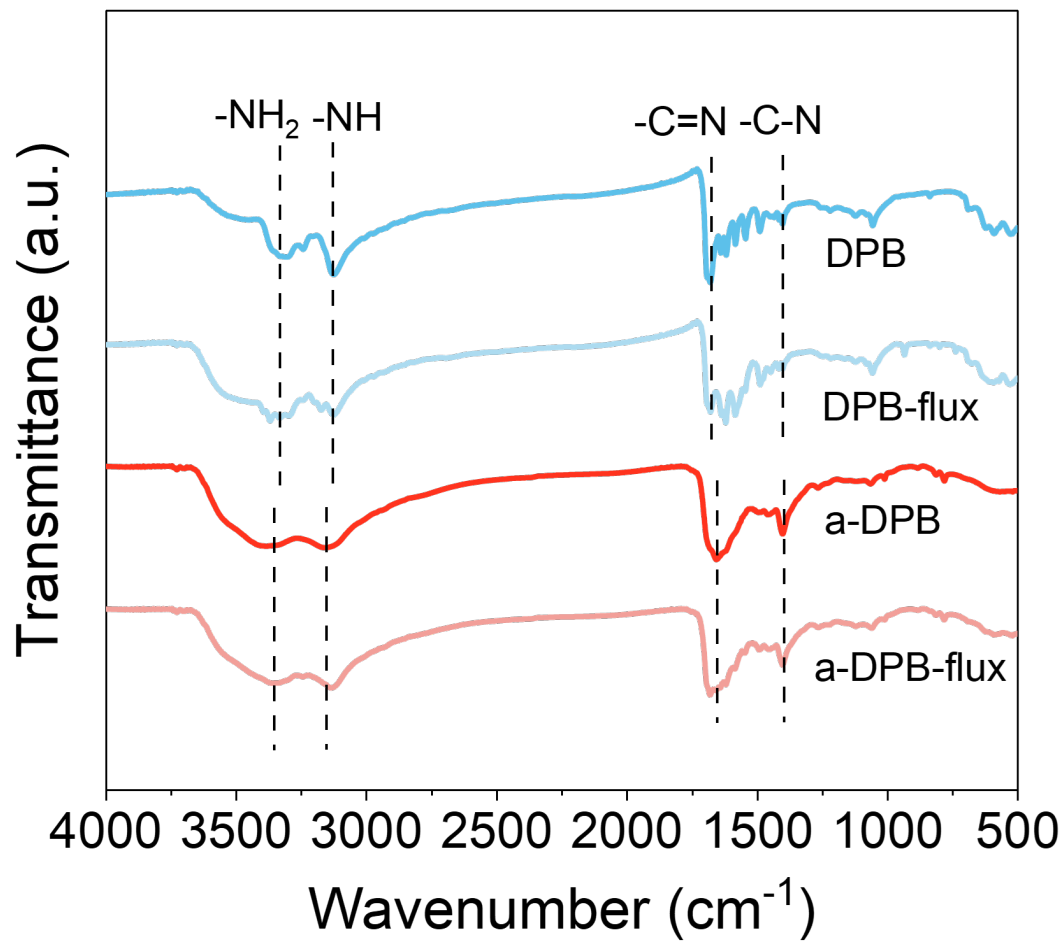

Figure R11. Fourier transform infrared (FTIR) spectra of doped and undoped DPB crystals and glass.

Comment 12: In Figure 1e, for 3.6 wt% LiOH, the T_m decreases, but the T_d-T_m window appears to narrow rather than widen. Please clarify the origin of this behavior.

Response: We thank the reviewer for the careful checking of our work. We found that the impact of LiOH content on T_d-T_m window is not strictly monotonic. At low-to-moderate LiOH concentrations, partial deprotonation effectively lowers T_m and expands the practical melt-processing window. However, at excessive LiOH loading, the increased amount of residual hydroxide/water and the higher density of deprotonated sites can enhance the ion mobility, and promote the degradation of the compounds. This behavior is the reason why we selected 1.1 wt% LiOH as the optimized composition, balancing sufficient T_m depression with acceptable T_g retention and material stability. This is also similar to the additives in perovskite devices, like solar cells, where too much additives can degradation the performance and lifetime of devices.

Comment 13: In Figure 2i, some compositions show a larger reduction in melting point upon LiOH addition than others. Does this suggest that each composition requires individual optimization of flux concentration due to differences in bond density or bonding types? Please comment.

Response: We thank the reviewer for the careful checking of our work. We agree that the degree of T_m depression is composition-dependent. The response to LiOH flux depends on several factors, including organic-cation acidity/accessibility, hydrogen-bond density, framework dimensionality, metal halide bond strength, and packing/steric constraints. Therefore, the flux concentration used in this work should be regarded as a general demonstration of the applicability of the strategy across multiple low-

dimensional systems, rather than a universal optimum for every composition. For practical device fabrication, each material system should be individually optimized to balance T_m depression, T_g retention, chemical stability, optical quality, and electronic performance.

Comment 14: SNPB and RNPB glasses are described as transparent with absorption edges around 357 nm in the literature, yet their photographs in the submitted manuscript appear yellowish/green, suggesting possible degradation (Figures 2j–k and S17). Please provide organic-to-inorganic compositional analysis to confirm composition preservation. Additionally, the kinks/valleys in the transmittance spectra suggest possible phase inhomogeneity. Please comment on phase purity.

Response: We thank the reviewer for the careful checking of our work. We agree that the color and spectral features of the RPB/SPB glasses require clarification. The undoped SPB and RPB samples have relatively narrow processing windows and high melt viscosities, and thus high temperature is required to ensure sufficient melt flow. This leads to organic-component degradation and yields dark-green products.

To examine composition preservation, we conducted elemental analysis of SPB glass (Figure R12). The C, N and H contents in the undoped glass are lower than those in both the flux-containing glass and the crystal, whereas the flux-containing glass shows values much closer to the crystalline composition. These results indicate that the greenish color of the undoped glass is mainly associated with some organic loss during high-temperature processing. For the flux-containing sample, the yellowish color in the photograph is likely influenced by sample thickness, bubbles or lighting conditions. A thin glass film with flux shows very good transparency (Figure R12).

We also note that there is weak absorption tail in the 400–500 nm range in previous studies (*Adv. Mater.* **33**, 2005868 (2021) Figure 3d), which may be related to light scatting or absorption from certain color centers. Give the large thickness of our glass materials, this effect would be more apparent. However, the current study still confirms the remarkably improved quality in these glasses with fluxes.

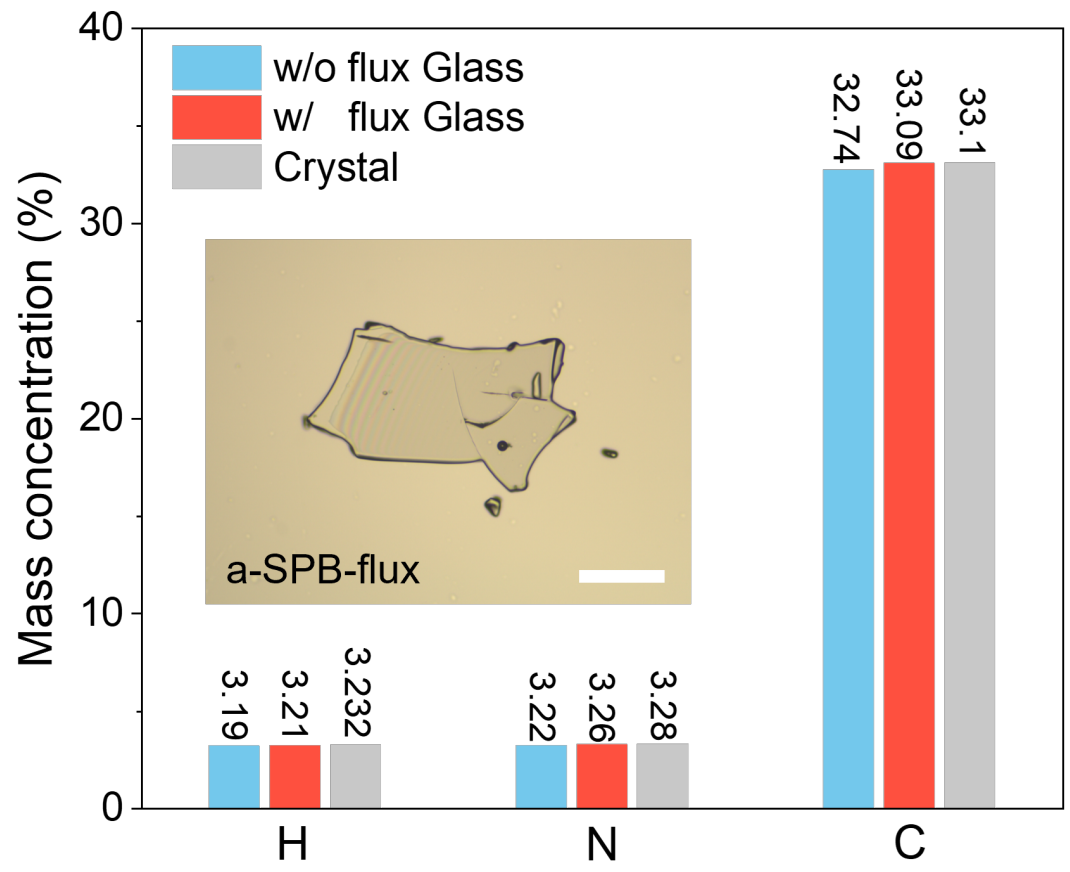

Figure R12. Elemental analysis of doped and undoped SPB glasses and undoped SPB crystals. Inset: optical microscopy image of a thin a-SPB-flux glass film (Scale bar: 100 μm).

Comment 15: In Figure 4b, the Pb–Br peak intensity/area correlating to coordination number decreases upon transitioning from crystalline to glassy states at ~2.5 Å. However, the peak intensity near ~1.5 Å increases in the opposite trend. Please explain the origin of this feature.

Response: We appreciate the reviewer's careful attention to the EXAFS analysis. Our coordination-number discussion is based on the first-shell Pb–Br feature at approximately 2.5–2.7 Å and the corresponding wavelet-transform signatures. The lower R feature near approximately 1.5 Å should not be assigned to a new Pb-centered coordination shell. Rather, it mainly arises from Fourier-transform/phase-shift effects, first-shell broadening, and background/windowing contributions, which become more visible when static disorder increases in the glassy state[28,29].

Besides, Raman spectra show no Pb–O vibrational modes, and the XANES edge confirms that Pb remains in the Pb^{2+} oxidation state. These results rule out appreciable Pb–O coordination and support our assignment of the dominant first-shell feature to Pb–Br coordination.

Comment 16: Please clarify whether DSC measurements were performed using open or hermetically sealed pans. If sealed, how does OH^{-} evolve or recombine during heating and cooling, and how might this influence crystallization behavior?

Response: We thank the reviewer for the careful checking of our work. We have now specified the DSC measurement conditions in the methods. The DSC measurements were performed using aluminum crucibles with a small opening under nitrogen flow. Under these conditions, water generated by deprotonation can partially effuse during

heating, which does not affect the forward neutralization/deprotonation process.

The detailed test conditions and discussion of this part have been added to the revised manuscript (page 24, line 577-580) and Supporting Information (page 34, line 274-279) as follows:

Differential scanning calorimetry (DSC) measurements were performed on a Netzsch DSC 404 F3 instrument under identical temperature conditions and gas flow parameters, with an aluminum crucible equipped with ventilation holes.

The reaction between oxoacid anions from the fluxes and hydrogen ions (H^+) from the organic cations is an acid-base neutralization reaction, reforming the original oxoacid. However, at elevated temperatures, these oxoacids degrade into volatile products. In an open environment, the removal of these products favors the forward direction of the reaction. The deprotonation induced by the acid-base neutralization reaction is most critical during the heating/melting process, whereas recombination upon cooling is limited by the rapid structural arrest of the glass and the local coordination of Li^+ with deprotonated organic/halide sites.

Comment 17: A shift in the melt-crystallization onset is observed during cyclic cooling of BPI. Please comment on the possible physical origin of this behavior.

Response: We appreciate the reviewer's careful observation. The initial shift should be related to many factors, including structural relaxation, redistribution/consumption of residual hydroxide, release of trace water, and gradual homogenization of Li^+ coordination during the early cycles. These changes can modify the effective nucleation barrier and shift the crystallization onset temperature. After the initial cycles, the crystallization and melting features become more reproducible, indicating that the BPI-flux system reaches a stable reversible crystal/melt transformation regime. We have added this explanation to the caption of Supporting Information Figure S28 (page24, line 172-180) as follows:

The initial shift in crystallization onset originates from several possible mechanisms, including the structural relaxation of the first melt, redistribution/consumption of residual hydroxide, release of trace water, and homogenization of Li^+ coordination during the early cycles. These changes alter the effective nucleation barrier and shift the crystallization onset. After the initial cycles, the crystallization/melting peaks become reproducible, indicating that the BPI-flux melt reaches a stable and reversible crystal/melt transformation regime. The consistency of each cycle in the melting and

crystallization peaks suggests excellent thermal stability of the BPI melt.

Additional Minor Suggestion

Please include page numbers in the manuscript footer or header to facilitate navigation for readers and reviewers.

• Legend colors are inconsistent between Figures S3 and S4. Please revise for consistency throughout the Supplementary Information.

• In the Introduction, references 11–14 should be arranged in chronological order based on the time of discovery.

• In the final paragraph of the Introduction, the narrative flow is disrupted by an abrupt transition from BA-based to DGA-based systems. Please revise for smoother logical continuity.

• Figure callouts do not appear in numerical/alphabetical order in the text. Please correct this throughout the manuscript.

• The word "increasement" is non-standard English. Please replace it with an appropriate alternative, such as "increase" or "enhancement." Overall, this is a strong and timely contribution, and I believe addressing the points above will further improve the clarity and rigor of the manuscript.

Response: We thank the reviewer for these helpful suggestions. We have carefully checked the manuscript and Supporting Information and made the requested corrections. Specifically, we have added page numbers to facilitate navigation, unified the legend colors throughout the Supporting Information, arranged the relevant Introduction references in chronological order, revised the final Introduction paragraph to provide a smoother transition from BA-based to DGA-based systems, corrected the order of figure callouts throughout the manuscript, and replaced the non-standard word "increasement" with "increase" or "enhancement" where appropriate. We again appreciate these suggestions, which have improved the clarity and presentation quality of the manuscript.

Reviewer #3 (Remarks to the Author):

This article reports on the fabrication of organic–inorganic hybrid perovskite glasses via a flux-assisted strategy, which lowers the melting point through a partial deprotonation mechanism. This strategy enables the preparation of high-quality perovskite glasses, and demonstrates broad universality across different material systems. Overall, this work holds significant implications for the synthetic chemistry of perovskite glass and recyclable optoelectronics. The manuscript is well prepared, and the results are comprehensive. It can be considered for publication after the following issues are resolved.

Response: We sincerely thank the reviewer for the positive assessment of our work and for the constructive comments. We have carefully revised the manuscript to address each point, including adding direct optical evidence for the newly obtained BPI and DPI glasses, clarifying the structural influence of LiOH grinding, evaluating possible Li^+ migration and dark-current drift, and updating the X-ray calibration and related device parameters. A detailed point-by-point response is provided below.

Comment 1: This study reduces the melting point through flux doping, which, unlike the organic component directed modification strategy with its inherent limitations, demonstrates superior convenience and universality. Although the two strategies differ, both lead to a change in the melting temperature of the material. Do they share a similar mechanism of action? The authors are requested to provide further explanation.

Response: We really appreciate the reviewer's recognition and positive comments on our work. We agree that the relationship between flux-mediated melting-point reduction and organic-component-directed modification should be clarified. Both strategies can lower the melting temperature by weakening organic-inorganic interfacial interactions and reducing the enthalpic penalty for the crystal-to-liquid transition. However, they implement this principle in different ways. Organic-component-directed modification changes the molecular structure, size, flexibility, and hydrogen-bonding pattern during material design, and therefore requires the synthesis or selection of new organic cations. In contrast, our flux-mediated strategy modifies the interfacial chemistry post-synthetically through partial deprotonation and local Li^+ coordination, thereby destabilizing the crystalline framework and activating flow units without redesigning the organic cation. Thus, the two approaches share some broad thermodynamic principles, but differ in chemical implementation, tunability, and practical universality.

Comment 2: Two non-melting systems, $(BA)_2PbI_4$ and $(DGA)PbI_4$, achieved low

temperature melting behavior through this strategy, and their corresponding glass transition temperatures were detected, which is impressive. However, the manuscript does not provide direct evidence of the two glasses. Please include corresponding optical or scanning electron microscopy images.

Response: We thank the reviewer for this helpful suggestion. We agree that direct optical evidence is valuable for demonstrating the glassy products obtained from the previously non-melting BPI and DPI systems. We have therefore added optical microscopy images of a-BPI-flux and a-DPI-flux (Figure R13). The bulk glass pieces were prepared by melting the materials in vacuum-sealed quartz tubes followed by rapid quenching in liquid nitrogen. The optical images show transparent, compact glass pieces without visible crystalline domains. These data complement the existing HR-TEM/SAED/EDS mapping and broad XRD patterns, providing more direct evidence for the vitrification of the BPI and DPI products. We have included the new images and discussion in the revised Supporting Information Figure S23 and Figure S24.

a b

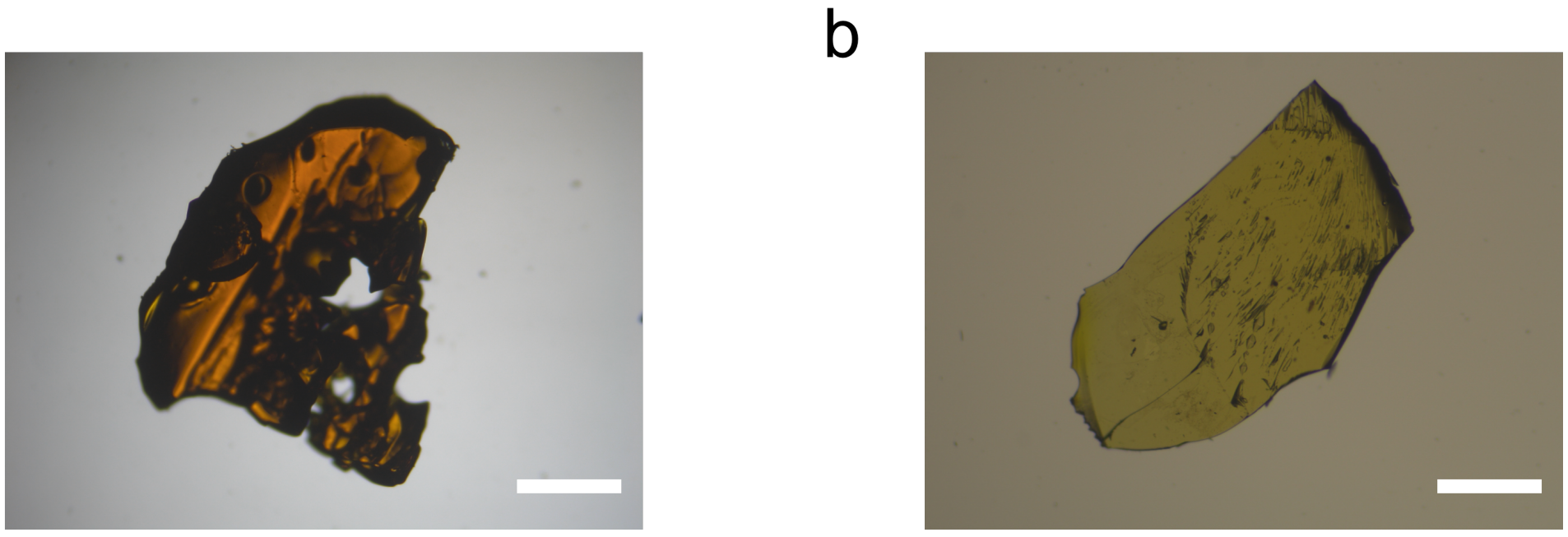

Figure R13. Optical microscopy images of (a) a-BPI-flux and (b) a-DPI-flux (Scale bar: 100 μm)

Comment 3: During the mixing and grinding process, the flux LiOH may undergo a partial deprotonation reaction with DPB crystals, causing changes in the crystal structure. Please provide XRD patterns of DPB at different doping ratios and explain the results.

Response: We thank the reviewer for this valuable suggestion. To examine whether LiOH causes structural changes during the initial mixing/grinding step, we have added powder XRD patterns of DPB with different LiOH doping ratios (Figure R14). At low-to-moderate LiOH loadings, the major DPB diffraction peaks are largely retained, with only minor intensity variations, indicating that room-temperature grinding induces only limited interfacial deprotonation and does not collapse the crystalline framework. At higher LiOH loadings, the diffraction intensity decreases more evidently, consistent

with stronger lattice distortion. These results support our proposed mechanism: the flux slightly destabilizes the crystal before heating, whereas the major structural reorganization occurs during thermal melting, where enhanced ion/molecule mobility enables more effective deprotonation and Li^+ coordination to activate the flow units. We have included the new XRD spectra and discussion in the revised Supporting Information Figure S33.

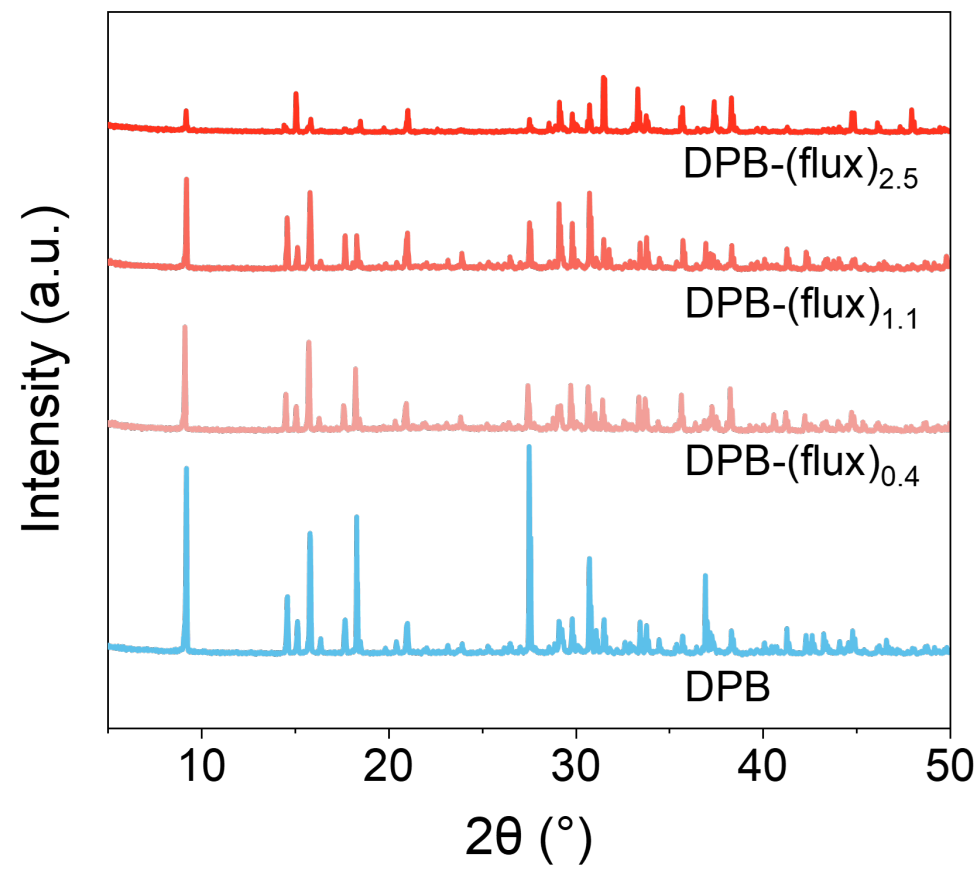

Figure R14. XRD patterns of DPB samples with varying LiOH doping ratios.

Comment 4: I am also concerned about the influence of the flux on the semiconductor properties of perovskite materials. Will lithium ions migrate during the operation of the device, thereby affecting the dark current and the long-term stability of the detector?

Response: We appreciate this valuable comment. To address your issue, we have introduced more tests and discussions. Quantitative Li/Pb analysis and DFT results indicate that trace Li^+ is locally coordinated at the deprotonated organic/halide interface, rather than acting as a highly mobile interstitial species (Figure R15a). The cyclic I-V curves under high electric fields exhibit weak hysteresis, and continuous bias measurements show a dark current drift of only 1.38×10^{-7} nA cm^{-1} s^{-1} V^{-1} (Figure R15b and c). Furthermore, the detector maintains its resistivity, $\mu\tau$ product, and sensitivity after multiple recycling cycles. These results demonstrate that Li^+ migration does not significantly affect dark current or long-term detector stability under the tested operating conditions.

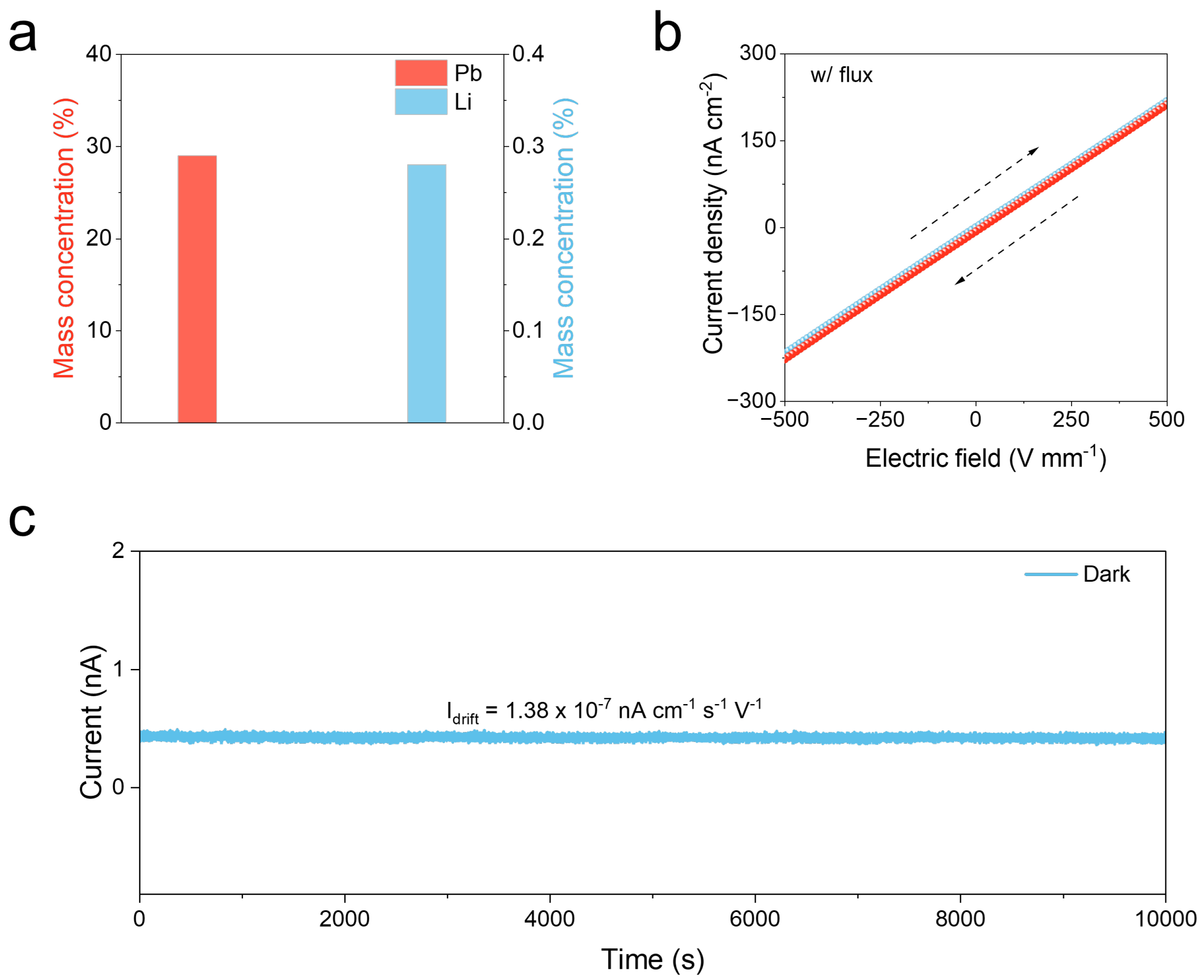

Figure R15. (a) ICP-OES results of the Li/Pb ratio in a-DPB-flux. (b) Cyclic dark I-V curves (10 V s^{-1}) of the Ag/a-DPB-flux/Ag device. (c) Dark current stability of Ag/a-DPB-flux/Ag devices under 500 V mm^{-1}.

Comment 5: The X-ray detector based on a-DPB-flux demonstrates excellent detection sensitivity and exhibits stable dark current under high electric fields. It is recommended to quantify the dark current drift value in Figure 5g. Is this excellent stability related to the disordered structure of the glass material? Please provide supporting evidence and elaborate.

Response: We thank the reviewer for this constructive suggestion. We have quantified the dark-current drift in Figure 5g and added cyclic I-V data measured under high electric fields (Figure R16). At 500 V mm^{-1}, the dark-current drift is only 1.38×10^{-7} nA cm^{-1} s^{-1} V^{-1}, and this value has been included in both the figure caption and the main-text discussion. We attribute the excellent stability partly to the glassy structure. The absence of grain boundaries removes fast ion/defect migration pathways[1-4], while the amorphous network and local Li^{+} coordination create a kinetically arrested matrix at room temperature. This interpretation is supported by the high resistivity, nearly hysteresis-free cyclic I-V behavior, stable dark current under continuous bias, stable

photocurrent under X-ray irradiation, and reproducible performance after repeated processing cycles. We have added these data and the corresponding discussion to the revised manuscript.

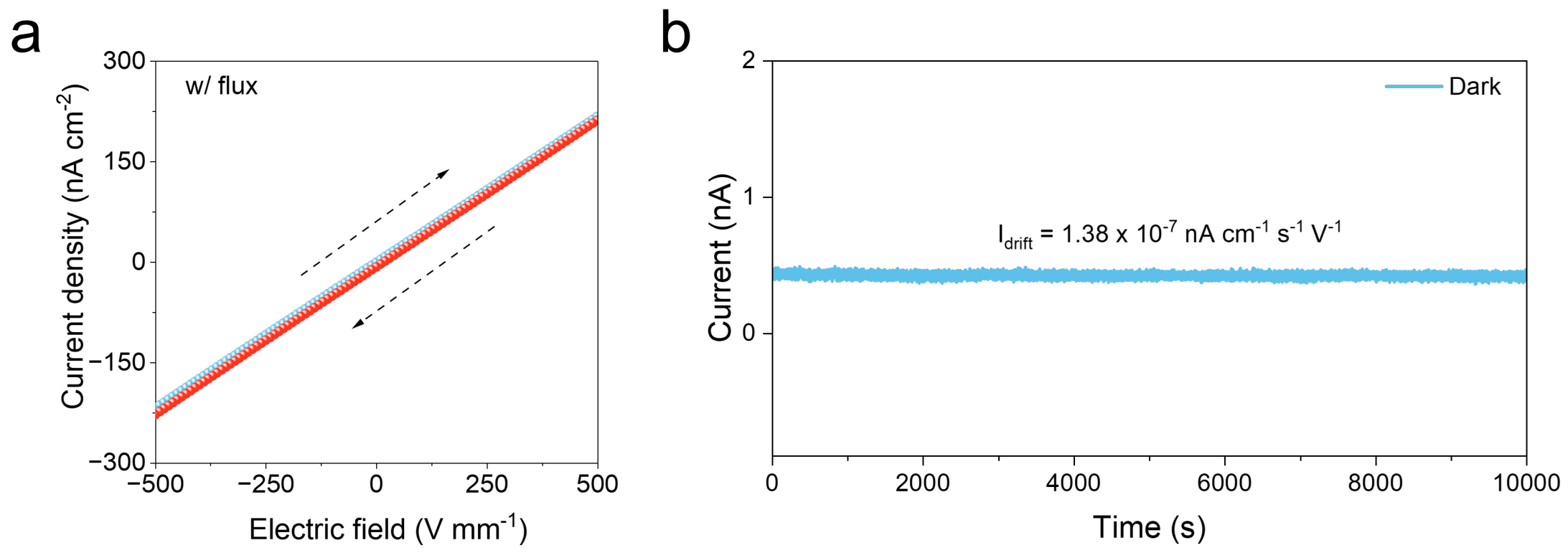

Figure R16. (a) Cyclic dark I-V curves (10 V s^{-1}) and (b) dark current stability assessment of the Ag/a-DPB-flux/Ag device under an electric field of 500 V mm^{-1}.

Comment 6: When reporting the device's sensitivity and detection limit, the average X-ray energy used in the experiment should also be considered and stated, as both sensitivity and detection limit clearly depend on the X-ray energy.

Response: We thank the reviewer for the comments. In this work, we employed a copper-target X-ray source operated at a tube voltage of 40 kVp with the peak energy at 8.05 keV. We placed a 1-mm-thick aluminum foil filter in the beam path throughout all measurements, and the dose rate was controlled by adjusting the tube current. For the attenuation calculations presented in Figure 5a of the main text, we used a photon energy of 40 keV as a representative diagnostic energy, which provides a conservative estimate of the material's attenuation capability. We greatly appreciate the reviewer's reminder, as this may reflect a long-overlooked yet crucial parameter for fair benchmarking within the community.

Comment 7: I found that the authors used the RAYSAFE X2 for X-ray calibration. Actually, this dosimeter is suitable for X-ray calibration over a photon energy range of 40–150 keV. Using it to calibrate the 40 kVp X-ray beam employed in this work would introduce significant uncertainty. The authors are recommended to recalibrate the X-ray dose rate using a proper dosimeter, such as a gas-ionization chamber, to ensure data reliability.

Response: We thank the reviewer for the valuable comment. We agree that the use of an appropriate dosimeter is essential for reliable sensitivity and detection-limit values, particularly for a 40 kVp X-ray beam. Following the reviewer’s recommendation, we

recalibrated the X-ray dose rate using a gas ionization chamber (PTW 34013), which is suitable for low-energy diagnostic X-ray calibration in the 15–70 kV range. Based on the recalibrated dose rate, we recalculated the relevant X-ray detector parameters and updated the corresponding values in the manuscript. The Methods section (page25, line 612-614), Figure 5, and the Supporting Information have been revised accordingly to ensure the reliability and traceability of the reported device performance.

Comment 8: There are some errors in the description. In line 223 of the main text, "whereby approximately 75%~85% of the hydroxide ions participate in protonation" should be corrected to "whereby approximately 75%~85% of the hydroxide ions participate in deprotonation." Please make this correction.

Response: We thank the reviewer's careful checking of our manuscript, and apologize for this error. We have corrected "protonation" to "deprotonation" in the revised manuscript.

References

1 Shao Y., *et al*. Grain boundary dominated ion migration in polycrystalline organic–inorganic halide perovskite films. *Energy Environ. Sci.* **9**, 1752-1759 (2016)

2 Ni Z., *et al*. High grain boundary recombination velocity in polycrystalline metal halide perovskites. *Sci. Adv*. **8**, eabq8345 (2022)

3 Xu Y., *et al*. Tailoring multi-phenyl ring cation for stable scalable hybrid bismuth iodide amorphous film: enabling record sensitivity and high-performance X-ray array imaging. *Adv. Mater*. **36**, 2406128 (2024).

4 Singh A., *et al*. Emergence of melt and glass states of halide perovskite semiconductors. *Nat. Rev. Mater.* **10**, 211-227 (2025).

5 Ye C., *et al*. Glass formation in hybrid organic-inorganic perovskites. *Angew. Chem. Int. Ed.* **62**, e202302406 (2023).

6 Singh A., *et al*. Local structure in crystalline, glass and melt states of a hybrid metal halide perovskite. *J. Am. Chem. Soc.* **146**, 25656-25668 (2024).

7 Ye C., *et al*. Structural dynamics of melting and glass formation in a two-dimensional hybrid perovskite. *Nat. Commun.* **16**, 7696 (2025).

8 Zhuang R., *et al*. Highly sensitive X-ray detector made of layered perovskite-like $(NH_4)_3Bi_2I_9$ single crystal with anisotropic response. *Nat. Photonics* **13**, 602-608 (2019).

9 Wang J., *et al*. Nonstoichiometry promoted solventless recrystallization of a thick and compact $CsPbBr_3$ film for real-time dynamic X-ray imaging. *Adv. Sci.* **11**, 2407314 (2024).

10 Wei W., *et al*. Monolithic integration of hybrid perovskite single crystals with heterogenous substrate for highly sensitive X-ray imaging. *Nat. Photonics* **11**, 315-321 (2017).

11 Chu D., *et al*. Stable and ultrasensitive X-ray detectors based on oriented single-crystal perovskite rods. *Adv. Mater.* **37**, 2500101 (2025).

12 Pan W., *et al*. $Cs_2AgBiBr_6$ single-crystal X-ray detectors with a low detection limit. *Nat. Photonics* **11**, 726-732 (2017).

13 Xu X., *et al*. Sequential growth of 2D/3D double-layer perovskite films with

superior X-ray detection performance. *Adv. Sci*. **8**, 2102730 (2021).

14 He X., *et al*. Quasi-2D perovskite thick film for X-ray detection with low detection limit. *Adv. Funct. Mater.* **32**, 2109458 (2021).

15 Ma C., *et al*. Centimeter-sized 2D perovskitoid single crystals for efficient X-ray photoresponsivity. *Chem. Mater.* **34**, 1699-1709 (2022).

16 Li H., *et al*. Sensitive and stable 2D perovskite single-crystal X-ray detectors enabled by a supramolecular anchor. *Adv. Mater.* **32**, 2003790 (2020).

17 Dai H., *et al*. Building high-density polar hybrid perovskites via intercalation of Cs^+ and aromatic diamine for passive X-ray detection. *ACS Appl. Mater. Interfaces* **16**, 42372-42379 (2024).

18 Feng X., *et al*. Ligand-assisted growth of 2D perovskite single crystal for highly sensitive X-ray detectors. *Adv. Funct. Mater.* **34**, 2402166 (2024).

19 Zhong H., *et al*. Multiple interlayer interactions enable highly stable X-ray detection in 2D hybrid perovskites. *JACS Au* **4**, 2393-2402 (2024).

20 Wu W., *et al*. Unprecedented mixed low-dimensional metal halide double perovskite for stable and efficient X-ray detection. *Laser Photonics Rev.* **19**, e00276 (2025).

21 Liu D., *et al*. Quasi-one-dimensional perovskite single crystals enabling decoupled ionic-electronic transport for sensitive and stable X-ray detection. *Angew. Chem. Int. Ed.* **65**, e24500 (2026).

22 Wu W., *et al*. Spontaneous cooling enables high-quality perovskite wafers for high-sensitivity X-ray detectors with a low-detection limit. *Adv. Sci*. **11**, 2410303 (2024).

23 Xu Y., *et al*. Evaporation crystallization of zero-dimensional guanidinium bismuth iodide perovskite single crystal for X-ray detection. *Inorg. Chem. Front.* **9**, 494-500 (2022).

24 Kasap S., *et al*. Amorphous selenium and its alloys from early xeroradiography to high resolution X-ray image detectors and ultrasensitive imaging tubes. *Phys. Status Solidi B* **246**, 1794-1805 (2009).

25 Kasap S. O., *et al*. X-ray sensitivity of photoconductors: application to stabilized a-Se. *J. Phys. D: Appl. Phys.* **33**, 2853-2865 (2000).

26 Tokuda S., *et al*. Preparation and characterization of polycrystalline CdZnTe films for large-area, high-sensitivity X-ray detectors. *J. Mater. Sci. Mater. Electron.* **15**, 1-8 (2004).

27 Zhu Y., *et al*. One-dimensional hybrid metal halide with polymer-like flexible inorganic chains and glass-forming ability. *J. Am. Chem. Soc*. **147**, 35627-35637 (2025).

28 Li B., *et al*. Emerging 0D hybrid metal halide luminescent glasses. *Adv. Mater*. **37**, 2415483 (2024).

29 He Z., *et al*. A universal strategy toward two-component organic-inorganic metal halide luminescent glasses and glass-crystal composites. *Sci*. *Adv*. **11**, eadu1982 (2025).

Reviewer Comments

The manuscript reports a systematic reduction in melting points across a series of hybrid metal halides spanning multiple dimensionalities and structural motifs through the introduction of a flux additive. While flux-mediated melting-point reduction has been historically demonstrated for other classes of materials, its application to hybrid metal halides is, to the best of my knowledge, presented here for the first time. The authors support their proposed mechanism with comprehensive characterization and further demonstrate the practical relevance of this approach through radiation detection and imaging/screening applications.

Although reduced melting behavior in 2D hybrid perovskites has previously been reported for Ruddlesden–Popper (RP) phases, this work extends the concept to Dion–Jacobson (DJ) phases and further enables melting in compositions that were not previously known to exhibit melting. The ability to tune the thermal properties of hybrid metal halides in this manner is promising for the development of next-generation glassy optoelectronic devices.

I commend the authors for the strong experimental results and the generally compelling discussion. I recommend the manuscript for publication after the following issues are addressed.

Specific Comments and Suggestions

1. In the first paragraph of the Results section and in the caption of Figure 1B, the statement "thermal energy is required to cross the barrier for glass transition" is scientifically inaccurate in this context. The discussion should refer to *melting* rather than glass formation. Please revise this here and elsewhere in the manuscript where applicable.

2. Molar ratios and weight ratios are used interchangeably throughout the manuscript, which may be confusing to readers. Please include a table clearly correlating molar ratios with the corresponding weight ratios for ease of reference.

3. During melting of flux-added hybrids, water effusion is observed. Does this process also contribute to degradation of the hybrid materials? Please clarify and, if possible, discuss supporting evidence.

4. If the flux facilitates constituent mobility, as reflected by a reduction in T_g, what are the implications for charge transport properties in crystalline vs glassy phases, both with and without flux addition? A brief discussion would strengthen the manuscript.

5. The reported transmittance values of 73–87%, claimed as the highest ever reported for metal halide glasses, should be supported with appropriate references

or moderated accordingly. Additionally, please clarify whether film or monolith thickness was accounted for when making this comparison.

6. Please provide a reference supporting the assignment of the amine proton peak in the ^{1}H NMR data shown in Figure S25.
7. The manuscript states that minimized decomposition leads to very similar bandgaps in crystalline and glass phases (Figure S26). However, prior studies on hybrid halides typically report a pronounced difference in absorption onset between these phases (Refer Bennett et. al and Mitzi et. al.). Please discuss why such a contrast is not observed here.
8. The argument that flux addition disrupts the organic–inorganic interface, lowering the enthalpy of melting/fusion, is well articulated and supported by characterization. However, the additional claim that *deprotonated organic cations provide increased molecular flexibility and entropy gain* requires further validation and should be decoupled from the enthalpy argument. If direct evidence is not available, I recommend omitting this interpretation.
9. For X-ray detection, the reported maximum sensitivity of 38,772.9 μC Gy_{air}^{-1} at 500 V/mm is described as top-tier for amorphous semiconductors. Please substantiate this claim by comparison with state-of-the-art materials (crystalline as well) and devices.
10. DSC data show crystallization upon cooling for BPI, whereas monoliths form glasses. Please specify the cooling rates used in DSC measurements and during monolith processing to clearly distinguish between characterization conditions and form-factor-dependent processing effects.
11. A shift in the C=N stretching mode in FTIR is attributed to flux addition in the glassy state and correlated to the corresponding NMR peak splitting (Figures 3C and S29), which instead compares crystalline vs glass states. To validate this correlation, FTIR spectra comparing crystalline vs glass states should also be provided and discussed.
12. In Figure 1e, for 3.6 wt% LiOH, the T_m decreases, but the $T_d - T_m$ window appears to narrow rather than widen. Please clarify the origin of this behavior.
13. In Figure 2i, some compositions show a larger reduction in melting point upon LiOH addition than others. Does this suggest that each composition requires individual optimization of flux concentration due to differences in bond density or bonding types? Please comment.
14. SNPB and RNPB glasses are described as transparent with absorption edges around 357 nm in the literature, yet their photographs in the submitted manuscript

appear yellowish/green, suggesting possible degradation (Figures 2j–k and S17). Please provide organic-to-inorganic compositional analysis to confirm composition preservation. Additionally, the kinks/valleys in the transmittance spectra suggest possible phase inhomogeneity. Please comment on phase purity.

15. In Figure 4b, the Pb–Br peak intensity/area correlating to coordination number decreases upon transitioning from crystalline to glassy states at ~2.5 Å. However, the peak intensity near ~1.5 Å increases in the opposite trend. Please explain the origin of this feature.

16. Please clarify whether DSC measurements were performed using open or hermetically sealed pans. If sealed, how does OH^- evolve or recombine during heating and cooling, and how might this influence crystallization behavior?

17. A shift in the melt-crystallization onset is observed during cyclic cooling of BPI. Please comment on the possible physical origin of this behavior.

Additional Minor Suggestion

- Please include page numbers in the manuscript footer or header to facilitate navigation for readers and reviewers.
- Legend colors are inconsistent between Figures S3 and S4. Please revise for consistency throughout the Supplementary Information.
- In the Introduction, references 11–14 should be arranged in chronological order based on the time of discovery.
- In the final paragraph of the Introduction, the narrative flow is disrupted by an abrupt transition from BA-based to DGA-based systems. Please revise for smoother logical continuity.
- Figure callouts do not appear in numerical/alphabetical order in the text. Please correct this throughout the manuscript.
- The word *"increasement"* is non-standard English. Please replace it with an appropriate alternative, such as *"increase"* or *"enhancement."*

Overall, this is a strong and timely contribution, and I believe addressing the points above will further improve the clarity and rigor of the manuscript.
